# Genetic architecture of congenital hypogonadotropic hypogonadism: insights from analysis of a Portuguese cohort

**DOI:** 10.1093/hropen/hoae053

**Published:** 2024-09-11

**Authors:** Josianne Nunes Carriço, Catarina Inês Gonçalves, Asma Al-Naama, Najeeb Syed, José Maria Aragüés, Margarida Bastos, Fernando Fonseca, Teresa Borges, Bernardo Dias Pereira, Duarte Pignatelli, Davide Carvalho, Filipe Cunha, Ana Saavedra, Elisabete Rodrigues, Joana Saraiva, Luisa Ruas, Nuno Vicente, João Martin Martins, Adriana De Sousa Lages, Maria João Oliveira, Cíntia Castro-Correia, Miguel Melo, Raquel Gomes Martins, Joana Couto, Carolina Moreno, Diana Martins, Patrícia Oliveira, Teresa Martins, Sofia Almeida Martins, Olinda Marques, Carla Meireles, António Garrão, Cláudia Nogueira, Carla Baptista, Susana Gama-de-Sousa, Cláudia Amaral, Mariana Martinho, Catarina Limbert, Luisa Barros, Inês Henriques Vieira, Teresa Sabino, Luís R Saraiva, Manuel Carlos Lemos

**Affiliations:** CICS-UBI, Health Sciences Research Centre, University of Beira Interior, Covilhã, Portugal; CICS-UBI, Health Sciences Research Centre, University of Beira Interior, Covilhã, Portugal; Sidra Medicine, Doha, Qatar; Sidra Medicine, Doha, Qatar; Serviço de Endocrinologia, Diabetes e Metabolismo, Hospital de Santa Maria, Centro Hospitalar Universitário Lisboa Norte, Lisboa, Portugal; Serviço de Endocrinologia, Diabetes e Metabolismo, Centro Hospitalar Universitário de Coimbra, Coimbra, Portugal; Serviço de Endocrinologia, Hospital de Curry Cabral, Centro Hospitalar Universitário Lisboa Central, Lisboa, Portugal; Unidade de Endocrinologia Pediátrica, Serviço de Pediatria, Centro Materno Infantil do Norte, Centro Hospitalar Universitário de Santo António, Porto, Portugal; Serviço de Endocrinologia e Diabetes, Hospital Garcia de Orta, Almada, Portugal; Serviço de Endocrinologia, Diabetes e Metabolismo, Centro Hospitalar Universitário de São João, Porto, Portugal; Serviço de Endocrinologia, Diabetes e Metabolismo, Centro Hospitalar Universitário de São João, Porto, Portugal; Serviço de Endocrinologia, Diabetes e Metabolismo, Centro Hospitalar Universitário de São João, Porto, Portugal; Serviço de Endocrinologia, Diabetes e Metabolismo, Centro Hospitalar Universitário de São João, Porto, Portugal; Serviço de Endocrinologia, Diabetes e Metabolismo, Centro Hospitalar Universitário de São João, Porto, Portugal; Serviço de Endocrinologia, Diabetes e Metabolismo, Centro Hospitalar Universitário de Coimbra, Coimbra, Portugal; Serviço de Endocrinologia, Diabetes e Metabolismo, Centro Hospitalar Universitário de Coimbra, Coimbra, Portugal; Serviço de Endocrinologia, Diabetes e Metabolismo, Centro Hospitalar Universitário de Coimbra, Coimbra, Portugal; Serviço de Endocrinologia, Diabetes e Metabolismo, Hospital de Santa Maria, Centro Hospitalar Universitário Lisboa Norte, Lisboa, Portugal; Serviço de Endocrinologia, Diabetes e Metabolismo, Centro Hospitalar Universitário de Coimbra, Coimbra, Portugal; Unidade de Endocrinologia Pediátrica, Serviço de Pediatria, Centro Materno Infantil do Norte, Centro Hospitalar Universitário de Santo António, Porto, Portugal; Unidade de Endocrinologia e Diabetologia Pediátrica, Departamento de Pediatria, Centro Hospitalar Universitário de São João, Porto, Portugal; Serviço de Endocrinologia, Diabetes e Metabolismo, Centro Hospitalar Universitário de Coimbra, Coimbra, Portugal; Serviço de Endocrinologia, Instituto Português de Oncologia do Porto, Porto, Portugal; Serviço de Endocrinologia, Instituto Português de Oncologia do Porto, Porto, Portugal; Serviço de Endocrinologia, Diabetes e Metabolismo, Centro Hospitalar Universitário de Coimbra, Coimbra, Portugal; Serviço de Endocrinologia, Diabetes e Metabolismo, Centro Hospitalar Universitário de Coimbra, Coimbra, Portugal; Serviço de Endocrinologia, Diabetes e Metabolismo, Centro Hospitalar Universitário de Coimbra, Coimbra, Portugal; Serviço de Endocrinologia, Instituto Português de Oncologia de Coimbra, Coimbra, Portugal; Unidade de Endocrinologia Pediátrica, Serviço de Pediatria, Hospital de Braga, Braga, Portugal; Serviço de Endocrinologia, Hospital de Braga, Braga, Portugal; Serviço de Pediatria, Hospital da Senhora da Oliveira Guimarães, Guimarães, Portugal; Departamento de Endocrinologia, Hospital da Luz Lisboa, Lisboa, Portugal; Serviço de Endocrinologia, Diabetes e Metabolismo, Centro Hospitalar Universitário de São João, Porto, Portugal; Serviço de Endocrinologia, Diabetes e Metabolismo, Centro Hospitalar Universitário de Coimbra, Coimbra, Portugal; Serviço de Pediatria, Centro Hospitalar do Médio Ave, Unidade de V. N. Famalicão, Vila Nova de Famalicão, Portugal; Serviço de Endocrinologia, Centro Hospitalar Universitário de Santo António, Porto, Portugal; Serviço de Endocrinologia, Centro Hospitalar do Tâmega e Sousa, Guilhufe, Portugal; Unidade de Endocrinologia Pediátrica, Hospital Dona Estefânia, Centro Hospitalar Universitário de Lisboa Central, Lisboa, Portugal; Serviço de Endocrinologia, Diabetes e Metabolismo, Centro Hospitalar Universitário de Coimbra, Coimbra, Portugal; Serviço de Endocrinologia, Diabetes e Metabolismo, Centro Hospitalar Universitário de Coimbra, Coimbra, Portugal; Serviço de Endocrinologia, Hospital de Curry Cabral, Centro Hospitalar Universitário Lisboa Central, Lisboa, Portugal; Sidra Medicine, Doha, Qatar; College of Health and Life Sciences, Hamad Bin Khalifa University, Doha, Qatar; Department of Comparative Medicine, Yale University School of Medicine, New Haven, CT, USA; CICS-UBI, Health Sciences Research Centre, University of Beira Interior, Covilhã, Portugal

**Keywords:** congenital hypogonadotropic hypogonadism, Kallmann syndrome, whole-exome sequencing, genetics, mutation

## Abstract

**STUDY QUESTION:**

What is the contribution of genetic defects in Portuguese patients with congenital hypogonadotropic hypogonadism (CHH)?

**SUMMARY ANSWER:**

Approximately one-third of patients with CHH were found to have a genetic cause for their disorder, with causal pathogenic and likely pathogenic germline variants distributed among 10 different genes; cases of oligogenic inheritance were also included.

**WHAT IS KNOWN ALREADY:**

CHH is a rare and genetically heterogeneous disorder characterized by deficient production, secretion, or action of GnRH, LH, and FSH, resulting in delayed or absent puberty, and infertility.

**STUDY DESIGN, SIZE, DURATION:**

Genetic screening was performed on a cohort of 81 Portuguese patients with CHH (36 with Kallmann syndrome and 45 with normosmic hypogonadotropic hypogonadism) and 263 unaffected controls.

**PARTICIPANTS/MATERIALS, SETTING, METHODS:**

The genetic analysis was performed by whole-exome sequencing followed by the analysis of a virtual panel of 169 CHH-associated genes. The main outcome measures were non-synonymous rare sequence variants (population allele frequency <0.01) classified as pathogenic, likely pathogenic, and variants of uncertain significance (VUS).

**MAIN RESULTS AND THE ROLE OF CHANCE:**

A genetic cause was identified in 29.6% of patients. Causal pathogenic and likely pathogenic variants were distributed among 10 of the analysed genes. The most frequently implicated genes were *GNRHR*, *FGFR1*, *ANOS1*, and *CHD7*. Oligogenicity for pathogenic and likely pathogenic variants was observed in 6.2% of patients. VUS and oligogenicity for VUS variants were observed in 85.2% and 54.3% of patients, respectively, but were not significantly different from that observed in controls.

**LARGE SCALE DATA:**

N/A.

**LIMITATIONS, REASONS FOR CAUTION:**

The identification of a large number of VUS presents challenges in interpretation and these may require reclassification as more evidence becomes available. Non-coding and copy number variants were not studied. Functional studies of the variants were not undertaken.

**WIDER IMPLICATIONS OF THE FINDINGS:**

This study highlights the genetic heterogeneity of CHH and identified several novel variants that expand the mutational spectrum of the disorder. A significant proportion of patients remained without a genetic diagnosis, suggesting the involvement of additional genetic, epigenetic, or environmental factors. The high frequency of VUS underscores the importance of cautious variant interpretation. These findings contribute to the understanding of the genetic architecture of CHH and emphasize the need for further studies to elucidate the underlying mechanisms and identify additional causes of CHH.

**STUDY FUNDING/COMPETING INTEREST(S):**

This research was funded by the Portuguese Foundation for Science and Technology (grant numbers PTDC/SAU-GMG/098419/2008, UIDB/00709/2020, CEECINST/00016/2021/CP2828/CT0002, and 2020.04924.BD) and by Sidra Medicine—a member of the Qatar Foundation (grant number SDR400038). The authors declare no competing interests.

WHAT DOES THIS MEAN FOR PATIENTS? Congenital hypogonadotropic hypogonadism (CHH) is a rare disorder characterized by delayed or absent puberty and infertility. Many cases remain unexplained despite extensive medical investigations. In this study, we aimed to determine what proportion of cases have a genetic origin. We performed advanced genetic studies on 81 Portuguese patients who had no explanation for their disorder. We found genetic mutations in approximately one-third of the patients, including several new mutations unknown to the scientific community. These results increase our understanding of the genetic basis of CHH and may contribute to improved diagnosis and treatment of patients.

## Introduction

Congenital hypogonadotropic hypogonadism (CHH) is a rare disorder characterized by deficient production, secretion, or action of GnRH. Affected individuals have low or inappropriately normal levels of gonadotropins (LH and FSH) and low levels of sex steroids, resulting in delayed or absent puberty and infertility (Boehm *et al.*, 2015; Young *et al.*, 2019).

CHH exhibits clinical heterogeneity and can be challenging to differentiate from constitutional delay of growth and puberty (Vezzoli *et al.*, 2023). CHH encompasses Kallmann syndrome (KS), characterized by GnRH deficiency combined with a full or partial defective sense of smell (anosmia or hyposmia, respectively), as well as CHH without olfactory defects (normosmic hypogonadotropic hypogonadism, nHH). Additional non-reproductive features may be present in CHH patients, such as renal agenesis, midline facial and brain defects, hearing impairment, and dental and skeletal defects (Boehm *et al.*, 2015; Young *et al.*, 2019).

The genetic basis of CHH is highly heterogeneous, involving multiple genes that encode essential components in the differentiation, migration, and function of GnRH neurons, as well as the regulation of gonadotropin synthesis and secretion (Grinspon 2021; Louden *et al.*, 2021). To date, ∼169 genes have been implicated in both isolated and syndromic forms of CHH (Supplementary Table S1). However, for many of these genes, the evidence linking them to CHH is not robust and requires further confirmation. While most cases are sporadic, familial forms also occur and can follow various inheritance patterns, including X-linked, autosomal-recessive, and autosomal-dominant patterns (Grinspon, 2021; Louden *et al.*, 2021). Nevertheless, the genetics of CHH are often complex due to factors such as incomplete penetrance, variable expressivity, and oligogenic inheritance (i.e. variants in two or more genes) (Butz *et al.*, 2021). Despite advances in the field, more than 50% of CHH patients still lack a confirmed genetic aetiology (Patil *et al.*, 2022a,b). Thus, understanding the genetic basis of CHH is essential for unravelling the underlying mechanisms of the disorder and improving diagnosis, genetic counselling, and potential therapeutic interventions.

The aim of this study was to identify and validate the genetic defects associated with CHH in a cohort of Portuguese patients.

## Materials and methods

### Subjects

The study included a total of 81 Portuguese patients diagnosed with idiopathic CHH and recruited from various clinical endocrine centres in Portugal. Attending physicians at these locations were responsible for the recruitment and collection of clinical data and blood samples of the patients. Inclusion criteria encompassed individuals with low or inappropriately normal serum levels of FSH, LH, and low serum levels of sex steroids, who had not entered puberty spontaneously by the age of 18 years. Patients with a history of acquired hypopituitarism, anatomical lesions in the hypothalamic–pituitary tract, and multiple pituitary hormone deficiencies were excluded from the study. The assessment of olfactory function was conducted either through olfaction testing or self-reporting, depending on the clinical centre. Among the patients, 36 had KS (32 males and 4 females) and 45 had nHH (38 males and 7 females). Eight patients had a family history of CHH, but only the index cases were included. A subset of this cohort had been previously studied by conventional Sanger sequencing of the *FGFR1* (Goncalves *et al.*, 2015; Fadiga *et al.*, 2022), *ANOS1* (*KAL1*) (Goncalves *et al.*, 2017b), *GNRHR* (Goncalves *et al.*, 2017a), and *CHD7* (Goncalves *et al.*, 2019) genes. The control population comprised 263 Portuguese individuals (50 healthy blood donors and 213 patients with unrelated disorders). Written informed consent was obtained from all subjects, and the study was approved by the Ethics Committee of the Faculty of Health Sciences, University of Beira Interior, Portugal (Ref: CE-FCS-2012-012 and CE-FCS-2011-003), and by the Institutional Review Board (IRB) for the protection of human subjects in Sidra Medicine, Qatar (IRB Ref: 1570003).

### Genetic analysis

Genomic DNA was extracted from peripheral blood leucocytes using previously described methods (Miller *et al.*, 1988). Whole-exome sequencing (WES) was performed on DNA samples from patients and controls, as previously described (Fadiga *et al.*, 2022). Genetic variants were filtered based on the following cumulative criteria: (i) location within the 169 genes associated with CHH reported in the literature (authors’ own compiled list, Supplementary Table S1); (ii) location in the canonical or commonly used coding transcript of these genes, as defined in the Human Genome Mutation Database (HGMD) (Stenson *et al.*, 2017); (iii) non-synonymous or located within two intronic nucleotides adjacent to coding exons; (iv) absence or rarity (allele frequency <0.01) in the Genome Aggregation Database (gnomAD, v2.1.1) and 1000 Genomes Project (Karczewski *et al.*, 2020); and (v) absence or rarity (allele frequency <0.01) in an in-house database of 263 Portuguese control individuals. The filtered variants were classified according to the American College of Medical Genetics and Genomics (ACMG) criteria (Richards *et al.*, 2015) and ClinGen recommendations (Pejaver *et al.*, 2022) as pathogenic (P), likely pathogenic (LP), variants of uncertain significance (VUS), likely benign (LB), or benign (B), using the web-based variant interpretation tool, Franklin (Genoox Ltd, https://franklin.genoox.com, accessed on 28 March 2024). P and LP variants were confirmed by conventional Sanger sequencing using a CEQ DTCS sequencing kit (Beckman Coulter, Fullerton, CA, USA) and an automated capillary DNA sequencer (GenomeLab TM GeXP, Genetic Analysis System, Beckman Coulter, Fullerton, CA, USA). The results of the WES analysis were combined with the results of Sanger sequencing that had been performed for the *FGFR1* (Goncalves *et al.*, 2015; Fadiga *et al.*, 2022), *ANOS1* (*KAL1*) (Goncalves *et al.*, 2017b), *GNRHR* (Goncalves *et al.*, 2017a), and *CHD7* (Goncalves *et al.*, 2019) genes. P and LP variants were considered to be causal if their zygosity was in agreement with the mode of inheritance of the gene (i.e., heterozygosity for autosomal dominant genes, homozygosity or compound heterozygosity for autosomal recessive genes, and hemizygosity for X-linked recessive genes) (Supplementary Table S1). Oligogenicity was defined as the presence of variants in two or more genes.

### Statistical analysis

The frequency of individuals with P variants, LP variants, VUS, and oligogenic inheritance was compared between patients and controls using Fisher’s exact test (GraphPad Prism, Version 7.04 for Windows, GraphPad Software, San Diego, CA, USA). The frequency of individuals with at least one VUS in each gene was compared between patients and controls using Fisher’s exact test and a modified Bonferroni-corrected *P*-value for multiple comparisons (by multiplying the *P*-value by 169, the number of genes). A *P*-value below 0.05 was considered statistically significant.

## Results

### Rare sequence variants identified in the 169 genes associated with CHH

Rare sequence variants (population allele frequency <0.01) were identified in 80 (98.8%) patients and 260 (98.9%) controls.

In the CHH patient cohort, a total of 323 rare sequence variants (279 unique variants) were identified. These variants included 15 P (12 unique), 28 LP (20 unique), 147 VUS (139 unique), 58 LB (49 unique), and 75 B (59 unique) variants (Supplementary Tables S2 and S3).

In the control population, a total of 1028 rare sequence variants (790 unique variants) were identified. These variants included 9 P (7 unique), 11 LP (8 unique), 544 VUS (488 unique), 167 LB (119 unique), and 297 B (168 unique) variants (Supplementary Tables S4 and S5).

### P and LP variants

**Figure 1.**
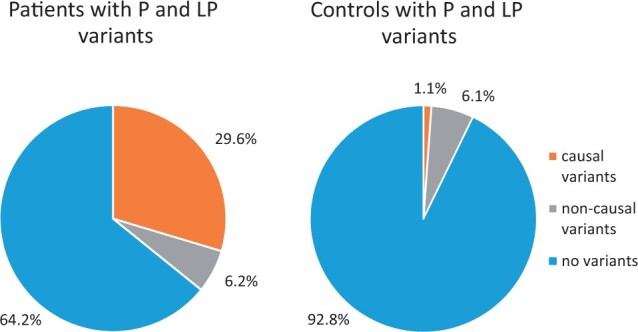
**Proportion of patients (n = 81) and controls (n = 263) with pathogenic and likely pathogenic variants in the analysed genes.** Causal pathogenic (P) and likely pathogenic (LP) variants (that are sufficient to explain the disease) were present in 29.6% of patients (left panel) and in only 1.1% of controls (right panel) (Fisher’s exact test for difference, *P* < 0.0001). Non-causal P and LP variants (that alone are not sufficient to explain the disease) were present in 6.2% and 6.1% of patients and controls, respectively (non-significant difference).

**Figure 2.**
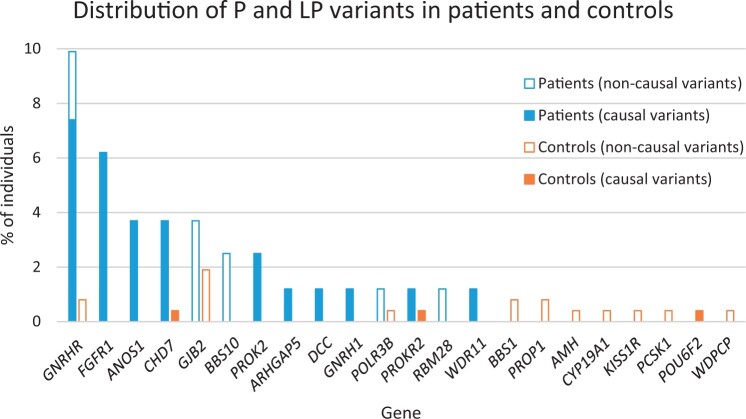
**Relative contribution of each gene with pathogenic and likely pathogenic variants in patients and controls.** Bars represent the frequency (%) of individuals with pathogenic (P) and likely pathogenic (LP) variants in each gene. Causal and non-causal P and LP variants are represented by solid and open bars, respectively.

Causal P and LP variants were identified in 24 (29.6%) of 81 patients (Fig. 1). These included 14 patients with causal variants already reported by the authors (Goncalves *et al.*, 2015, 2017a,b, 2019; Fadiga *et al.*, 2022) and 10 patients with causal variants described here for the first time (Supplementary Table S3). Causal variants were distributed across 10 genes: *GNRHR* (six patients, 7.4%), *FGFR1* (five patients, 6.2%), *ANOS1* and *CHD7* (three patients, 3.7%, each), *PROK2* (two patients, 2.5%), *ARHGAP5*, *DCC*, *GNRH1*, *PROKR2*, and *WDR11* (one patient, 1.2%, each) (Fig. 2). These causal variants were present in the homozygous (*GNRHR*, *PROK2*, and *GNRH1*), compound heterozygous (*GNRHR*), hemizygous (*ANOS1*), and heterozygous (*FGFR1*, *CHD7*, *ARHGAP5*, *DCC*, *PROKR2*, and *WDR11*) states.

In contrast, causal P and LP variants were identified in only 3 (1.1%) of the 263 controls (Fisher’s exact test for difference, *P* < 0.0001) (Fig. 1 and Supplementary Table S5). These were heterozygous variants located in the *CHD7*, *POU6F2*, and *PROKR2* genes (Fig. 2).

There were 5 (6.2%) additional patients and 16 (6.1%) additional controls who had heterozygous P and LP variants in autosomal recessive genes, which alone were not expected to cause disease and therefore were considered non-causal variants (Figs 1 and 2).

### Variants of uncertain significance

**Figure 3.**
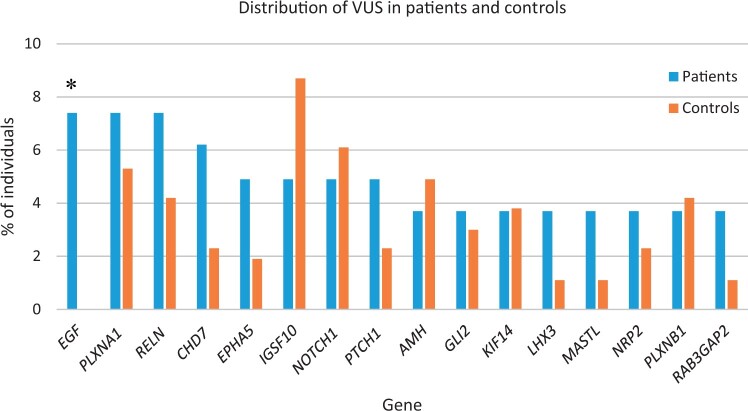
**Relative contribution of each gene with variants of uncertain significance in patients and controls.** Bars represent the frequency (%) of individuals with variants of uncertain significance (VUS) in each gene. Only the most commonly affected genes are represented. (The full list of VUS variants are in Supplementary Tables S2 and S4). Patients presented a higher frequency of VUS in the *EGF* gene (Fisher’s exact test, *P* = 0.0001; Bonferroni corrected *P* = 0.0169) compared to controls (asterisk).

VUS were identified in 69 (85.2%) patients and in 239 (90.9%) controls (difference not statistically significant). The most commonly affected genes in patients were *EGF*, *PLXNA1*, and *RELN* (7.4% of patients, each), *CHD7* (6.2%), *EPHA5*, *IGSF10*, *NOTCH1*, and *PTCH1* (4.9%, each), *AMH*, *GLI2*, *KIF14*, *LHX3*, *MASTL*, *NRP2*, *PLXNB1*, and *RAB3GAP2* (3.7%, each) (Fig. 3). The controls exhibited similar frequencies of VUS in these genes, except for *EGF*. Patients had a higher frequency of VUS in the *EGF* gene compared to controls (7.4% vs 0%, Fisher’s exact test, *P* = 0.0001; Bonferroni corrected *P* = 0.0169) (Fig. 3).

Five patients (6.2%) were compound heterozygotes for VUS (*CCDC141*, *EGF*, *IGSF10*, *MTOR*, *IFT172*, and *RELN* genes, the last two in the same patient), and three patients (3.7%) were hemizygous (one with an *ANOS1* variant, one with a *PHF6* variant, and one with a *PLXNA3* variant). The remaining VUS in patients were identified in the heterozygous state (Supplementary Table S3). In controls, one individual (0.4%) was homozygous for a VUS variant (*RELN* gene), eight (3.0%) were compound heterozygotes (*CCDC141*, *CHD7*, *HESX1*, *NOS1*, *NOTCH1*, *PLXNB1*, *SEMA7A*, and *WDR4* genes), and three (1.1%) were hemizygous (*PLXNA3* and *POLA1* genes). The remaining VUS in the controls were identified in a heterozygous state (Supplementary Table S5).

### Oligogenicity

Considering all P and LP variants (causal and non-causal), five patients (6.2%) had variants in two genes (two patients with variants in *GNRHR* and *GJB2*, one with variants in *GNRHR* and *RBM28*, one with variants in *ANOS1* and *POLR3B*, and one with variants in *BBS10* and *WDR11*). In contrast, only one control (0.4%) had LP variants in two genes (*AMH* and *PROKR2*) (Fisher’s exact test, *P* = 0.0032). For VUS variants, the frequency of oligogenicity was 54.3% in patients and 61.6% in controls (difference not statistically significant). Among patients, 25 (30.9%) had VUS variants in two genes, 10 (12.3%) had variants in three genes, and 9 (11.1%) had variants in four genes. Among controls, 85 (32.3%) had VUS variants in two genes, 45 (17.1%) had variants in three genes, 20 (7.6%) had variants in four genes, 5 (1.9%) had variants in five genes, 5 (1.9%) had variants in six genes, and 2 (0.8%) had variants in seven genes (difference not statistically significant).

### Frequency of variants according to phenotype

The frequency of causal P and LP variants in patients with KS and nHH was 38.9% (14 out of 36) and 22.2% (10 out of 45), respectively. The frequency of VUS variants in patients with KS and nHH was 80.6% (29 out of 36) and 88.9% (40 out of 45), respectively. These differences were not statistically significant.

Among the most commonly implicated genes, causal P and LP variants in *GNRHR* were exclusively associated with nHH and those in *ANOS1* were exclusively associated with KS (Supplementary Table S3).

Causal P and LP variants were identified in 5 out of 13 patients with a history of cryptorchidism (three in *FGFR1*, one in *ARHGAP5*, and one in *GNRH1*), in 2 out of 8 patients with hearing impairment (*CHD7* and *FGFR1* genes), and in 2 out of 2 patients with renal agenesis (*ANOS1* gene) (Supplementary Table S3). No statistically significant differences were observed between the frequencies of causal variants in patients with and without these phenotypes.

Causal P and LP variants were identified in seven out of eight patients with a family history of CHH (Supplementary Table S3).

## Discussion

Our study identified a genetic cause in 29.6% of Portuguese patients with CHH, which is consistent with the reported prevalence in other CHH cohorts. Previous systematic reviews estimated the worldwide prevalence of causative genetic variants to be ∼31% in KS (Patil *et al.*, 2022b) and 23% in nHH (Patil *et al.*, 2022a). However, there is considerable variability in prevalence data across individual studies that may be related to factors such as geographical/ethnic differences in the genetic background, proportion of familial cases, severity of the reproductive phenotype, and ascertainment bias (Patil *et al.*, 2022a,b). Notably, the size of the gene panel analysed in each study does not significantly correlate with the rate of genetic diagnosis, as the majority of P variants are confined to a limited number of CHH genes, beyond which the contribution of P variants is very small (Patil *et al.*, 2022a,b). Indeed, a recent survey of 26 health care providers from 13 European countries showed that the median number of genes analysed in customized CHH panels was only 36 (Persani *et al.*, 2022). For example, the UK NHS gene panel for CHH is currently 22 genes only (https://nhsgms-panelapp.genomicsengland.co.uk/panels/650/v3.0) and its use would have diagnosed almost all of our patients with causal variants, except two with *ARHGAP5* and *DCC* variants.

It is important to distinguish true causative variants from VUS that may be erroneously categorized as disease-causing. The widespread use of next-generation sequencing and the growing number of candidate genes for CHH have led to the identification of numerous VUS, for which there is insufficient evidence of their involvement in the disease. These often represent coincidental findings and, over time, many are reclassified as B variants (Burke *et al.*, 2022). However, several studies of CHH have included VUS as causative variants, thus overestimating the frequency of genetically caused CHH (Patil *et al.*, 2022a,b). In our study, we applied the stringent ACMG criteria to classify variants as P or LP (Richards *et al.*, 2015), ensuring a high level of confidence in establishing the link between the genetic variant and the disorder.

To understand the relative contribution of each gene in our CHH cohort, we combined the causal variants in 14 patients that had already been reported by us (Goncalves *et al.*, 2015, 2017a,b, 2019; Fadiga *et al.*, 2022) with those in 10 patients described here for the first time. Altogether, in our CHH cohort, we identified causal P and LP variants in 10 of the 169 analysed genes. The most frequently involved genes were *GNRHR*, *FGFR1*, *ANOS1*, and *CHD7*, consistent with the current knowledge on the main genetic causes of CHH (Grinspon 2021; Louden *et al.*, 2021). Other less frequently involved genes were *ARHGAP5*, *DCC*, *GNRH1*, *PROK2*, *PROKR2*, and *WDR11*. The *GNRH1* and *GNRHR* genes encode GnRH and its receptor, respectively, and P variants in these can lead to lack of secretion or action of GnRH (de Roux *et al.*, 1997; Chan *et al.*, 2009). The *FGFR1* (encoding fibroblast growth factor receptor-1), *ANOS1* (anosmin-1), *PROK2* (prokineticin-2), *PROKR2* (prokineticin receptor-2), *DCC* (netrin receptor), *ARHGAP5* (Rho GTPase-activating protein 5), and *WDR11* (WD repeat-containing protein 11) genes are involved in the development and migration of the GnRH neurons (Oleari *et al.*, 2021; Lippincott *et al.*, 2022). P variants in *CHD7* (chromodomain helicase DNA-binding protein-7) are associated with CHARGE syndrome (Coloboma of the eye, Heart defects, Atresia of the choanae, Retardation of growth and development, Genital hypoplasia and Ear abnormalities) (Marcos *et al.*, 2014). However, missense variants in this gene have been found in isolated CHH with minor or no other syndromic manifestations (Marcos *et al.*, 2014).

We identified additional P and LP variants in the *BBS10* (Bardet-Biedl syndrome-10), *GJB2* (gap junction protein, beta-2), *GNRHR*, *POLR3B* (polymerase III, RNA, subunit B), and *RBM28* (RNA-binding motif protein-28) genes. However, these genes are autosomal recessive, and our patients with these variants were all heterozygous. Therefore, these were considered non-causal variants and likely reflect the prevalence of healthy carriers in the general population, as suggested by the similarity between the frequency of non-causal P and LP variants in patients and controls (6.2% and 6.1%, respectively).

Despite the large number of analysed genes, causal P and LP variants were identified in only a small subset of these (10 out of 169 genes). The apparent lack of causal variants in the majority of these genes may have several explanations. First, the selection of genes for the virtual panel was based on their previous association with at least one patient with CHH in the scientific literature (Supplementary Table S1). This means that we included genes that had been implicated in single cases of patients without confirmatory studies. It is possible that such reported associations were spurious and that these genes do not truly contribute to CHH. Therefore, our panel may have over-represented the number of genes involved in this disorder. Second, we included genes that are associated with syndromes in which CHH occurs in the context of more complex clinical features that were not present in our patients. These genes were analysed due to the possibility of isolated CHH occurring in the context of variable expressivity of the syndrome, as in the case of mild variants in *CHD7* causing CHH in the absence of other features of CHARGE syndrome (Marcos *et al.*, 2014). However, it is possible that variants in some of these genes do not cause isolated CHH as the sole phenotype. Lastly, the contribution of some genes to CHH could be so rare that it would require a larger number of patients to find causative variants in these genes.

Despite our extensive analysis, approximately two-thirds of our patients remained without a genetic diagnosis. These mutation-negative patients could be attributed to several factors, including phenocopies where environmental or epigenetic factors play a role (Chung and Tsai, 2023), unidentified causative genes that were not included in the gene panel, or more complex genetic alterations that are not easily detected through WES, such as copy number variants or variants in noncoding regions of the genome. However, although additional CHH genes continue to be reported, their contribution to the overall genetic burden of CHH is becoming increasingly marginal. In addition, although copy number variants have been reported in CHH, these represent <2% of cases (Izumi *et al.*, 2014; Stamou *et al.*, 2022).

P and LP variants were also identified in our unaffected controls. However, these were mostly heterozygous variants in genes that have an autosomal recessive mode of inheritance or for which the mode of inheritance is still unclear. Therefore, these variants likely reflect the normal frequency of healthy carriers in the general population. Nonetheless, we observed likely P variants in the autosomal dominant genes *CHD7*, *POU6F2*, and *PROKR2* in three controls, suggesting either false-positive predictions of pathogenicity or incomplete penetrance of the variants in these unaffected individuals.

Our study identified a large number of VUS for which there is insufficient evidence to establish a role in the disorder. The frequency of VUS in patients (85.2%) was similar to that in unaffected controls (90.9%). This suggests that the VUS identified in the patients are likely unrelated to the disorder and reflect the genetic background of the general population. Interestingly, patients exhibited a higher frequency of VUS in the *EGF* gene compared to controls, although the significance of this finding remains to be determined.

Oligogenicity has been documented in CHH and is defined by variants in two or more genes, each insufficient to cause the disorder, but that together have a cumulative and synergistic effect. Oligogenicity may also explain how heterozygous variants in autosomal recessive genes could contribute to the overall genetic burden of CHH. Previous studies reported oligogenic variants in up to 20% of CHH patients (Sykiotis *et al.*, 2010; Butz *et al.*, 2021). However, recent systematic reviews, after eliminating VUS variants, showed a much lower frequency of oligogenicity (<5%) (Patil *et al.*, 2022a,b). Thus, it is possible that oligogenicity in CHH has been overestimated in the past due to the inclusion of VUS variants in the analyses. Our study found only five patients (6.2%) with oligogenicity involving P variants, and the frequency of oligogenicity for VUS variants was similar between patients and controls (54.3% and 61.6%, respectively). These findings suggest that the contribution of oligogenicity to the disorder in our cohort is unlikely to be significant.

In terms of phenotype, our study found a similar prevalence of causal P and LP variants in both KS and nHH patients. This contrasts with some previous studies that reported a higher prevalence in KS (Patil *et al.*, 2022a,b). In addition, we did not find a higher prevalence of variants in more severe forms of CHH, such as those associated with cryptorchidism. Consistent with previous knowledge, causal P and LP variants in *ANOS1* were exclusively associated with KS, while those in *GNRHR* were exclusively associated with nHH. However, the limited number of patients with variants in each gene precluded any further genotype–phenotype correlations.

Our study has certain limitations. The identification of a large number of VUS presents challenges in interpretation and these may require reclassification as more evidence becomes available. Furthermore, we did not analyse synonymous variants nor those located further away from the exons, which may rarely impact exon splicing and cause disease (Wachs and Bohne, 2021). In addition, copy-number variants were not analysed, although their contribution to the molecular diagnosis of CHH is generally limited (Izumi *et al.*, 2014; Stamou *et al.*, 2022). Moreover, the lack of data from parents and other family members did not allow for segregation studies to track the inheritance of the variants in cases of oligogenicity. Lastly, functional studies to elucidate the consequences of the identified genetic variants on protein function were not conducted.

In conclusion, our study revealed causal P and LP variants in 29.6% of patients with CHH, distributed among 10 of the 169 analysed genes. Several novel variants were identified, and these expand the known mutational spectrum of the disorder. While a large number of VUS variants were identified, their frequency in patients did not differ significantly from that of controls. The genetic heterogeneity and complex inheritance observed in CHH highlight the challenges in fully understanding its genetic architecture. Nevertheless, our findings contribute to the growing understanding of CHH genetics and have implications for improved diagnosis and management of this rare disorder. Future studies incorporating larger cohorts, functional analyses, and investigation of noncoding regions of the genome may provide further insights into the genetic basis of CHH.

## Supplementary Material

hoae053_Supplementary_Data

## Data Availability

The data underlying this article are available in the article and the online supplementary material.
