# Supplementary material for: Genetic architecture of congenital hypogonadotropic hypogonadism: insights from analysis of a Portuguese cohort
Source: Hum Reprod Open. 2024 Sep 11;2024(3):hoae053. doi: 10.1093/hropen/hoae053 (PMC11415827; doi:10.1093/hropen/hoae053)
Supplement: hoae053_Supplementary_Data [file hoae053_supplementary_data.zip › Supplementary Table S2.docx]

**Supplementary Table S2.** Rare sequence variants identified in patients with CHH.

| **Gene** | **Reference sequence** | **Variant** | **Effect** | **Allele frequency in GnomAD** | **Allele frequency in Portuguese controls** | **ACMG classification** | **Patient id** | **Previous report** |
| --- | --- | --- | --- | --- | --- | --- | --- | --- |
| *AMH* | NM_000479.3 | c.23G>C (p.Ser8Thr) | Missense | 0.000143 | - | VUS | 5191 | - |
|  | NM_000479.3 | c.295A>T (p.Thr99Ser) | Missense | 0.000219 | - | VUS (PM2) | 5943 | PMID: 28505284 |
|  | NM_000479.3 | c.428C>T (p.Thr143Ile) | Missense | 0.005473 | 0.006 | B (BA1, BS2, BP4, BP6) | 5183, 6319 | PMID: 33202802 |
|  | NM_000479.3 | c.974A>G (p.Gln325Arg) | Missense | 0.005543 | 0.002 | B (BA1, BS2, BP6) | 5404 | PMID: 1483695 |
|  | NM_000479.3 | c.1054C>T (p.Pro352Ser) | Missense | 0.000476 | 0.004 | VUS | 7074 | PMID: 28505284 |
| *ANOS1* | NM_000216.4 | c.31A>G (p.Thr11Ala) | Missense | - | - | VUS (PM2, BP4) | 8302 | - |
|  | NM_000216.4 | c.542-1G>C | Splice-site | - | - | LP (PVS1, PM2) | 5102 | PMID: 28122887* |
|  | NM_000216.4 | c.571C>T (p.Arg191*) | Nonsense | - | - | P (PVS1, PM2, PM3, PP1) | 7074 | PMID: 11297579 PMID: 28122887* |
|  | NM_000216.4 | Gross deletion ~ 4.8Mb | Gross Deletion | - | - | P | 6776 | PMID: 28122887* |
| *ARHGAP35* | NM_004491.5 | c.2495G>A (p.Arg832Gln) | Missense | 0.000100 | - | VUS (PM2, PP2) | 5237 | - |
| *ARHGAP5* | NM_001030055.2 | c.805G>C (p.Val269Leu) | Missense | 0.000021 | - | VUS (PM2, PP2) | 5187 | - |
|  | NM_001030055.2 | c.2067dupA (p.Tyr690Ilefs*4) | Frameshift | - | - | LP (PVS1, PM2) | 8214 | - |
|  | NM_001030055.2 | c.4216G>A (p.Ala1406Thr) | Missense | 0.005966 | 0.004 | B (PP2, BS1, BS2) | 5182, 5183, 7081, 7013 | - |
|  | NM_001030055.2 | c.4430T>G (p.Val1477Gly) | Missense | 0.000081 | - | VUS (PM2, PP2, BP4) | 7386 | - |
| *AXL* | NM_021913.3 | c.1829G>T (p.Arg610Leu) | Missense | 0.000020 | - | VUS (PM2) | 8302 | - |
| *BBS10* | NM_024685.4 | c.273C>G (p.Cys91Trp) | Missense | 0.000028 | - | P (PS3, PM2, PM3, PP2) | 5187 | PMID: 16582908 |
|  | NM_024685.4 | c.1837T>C (p.Tyr613His) | Missense | 0.000044 | - | LP (PM1, PM2, PM5, PP3) | 5040 | PMID: 16582908 |
| *CCDC141* | NM_173648.4 | c.1394G>C (p.Gly465Ala) | Missense | 0.000188 | 0.002 | VUS (PM2, BP4) | 7335 | - |
|  | NM_173648.4 | c.1396delT (p.Tyr466Thrfs*33) | Frameshift | 0.000188 | 0.002 | VUS (PM2) | 7335 | - |
|  | NM_173648.4 | c.1521A>C (p.Gln507His) | Missense | 0.003767 | 0.006 | LB (PM2, BS2, BP4, BP6) | 7381, 7074, 7359 | - |
| *CCDC88C* | NM_001080414.4 | c.322G>A (p.Gly108Ser) | Missense | 0.000225 | - | VUS (PM2) | 6980 | - |
|  | NM_001080414.4 | c.925C>T (p.Arg309Cys) | Missense | 0.000054 | - | VUS (PM2) | 5185 | - |
|  | NM_001080414.4 | c.2393C>T (p.Ala798Val) | Missense | 0.000272 | - | LB (BS1, BP4, BP6) | 5135 | PMID: 29590070 |
|  | NM_001080414.4 | c.4327G>A (p.Ala1443Thr) | Missense | 0.001922 | 0.008 | B (BA1, BS1, BS2, BP4, BP6) | 2661 | PMID: 29499638 |
|  | NM_001080414.4 | c.5251G>A (p.Val1751Ile) | Missense | 0.001183 | - | LB (BS1, BS2, BP4, BP6) | 6178 | - |
|  | NM_001080414.4 | c.5927G>C (p.Gly1976Ala) | Missense | 0.000767 | 0.004 | LB (BS1, BP4) | 5183, 8500 | - |
|  | NM_001080414.4 | c.5980C>G (p.Arg1994Gly) | Missense | 0.006488 | 0.004 | B (BA1, BS2, BP4, BP6) | 7013 | - |
| *CCKAR* | NM_000730.3 | c.1093G>A (p.Val365Ile) | Missense | 0.002210 | - | B (BS1, BS2, BP6) | 6024 | PMID: 11773861 |
| *CCKBR* | NM_176875.2 | c.199A>G (p.Met67Val) | Missense | - | - | VUS (PM2, BP4) | 6024 | - |
| *CHD4* | NM_001273.3 | c.86C>G (p.Pro29Arg) | Missense | - | - | VUS (PM2, PP2) | 7030 | - |
|  | NM_001273.3 | c.1715G>A (p.Arg572Gln) | Missense | - | - | VUS (PM2, PP2, PP3) | 5194 | PMID: 29844320 |
| *CHD7* | NM_017780.3 | c.1018A>G (p.Met340Val) | Missense | 0.004606 | 0.002 | B (PP2, BA1, BS2, BP6) | 6178, 7390 | PMID: 18073582 |
|  | NM_017780.3 | c.1163G>A (p.Gly388Glu) | Missense | 0.000004 | - | VUS (PM2, PP2) | 5178 | PMID: 30733481* |
|  | NM_017780.3 | c.1808_1811delACAA (p.Asn603Thrfs*4) | Frameshift | - | - | P (PVS1, PS4, PM2) | 7381 | PMID: 19159393 |
|  | NM_017780.3 | c.2185A>G (p.Lys729Glu) | Missense | 0.000356 | 0.002 | LB (PP2, BS2, BP6) | 2661 | PMID: 33270637 |
|  | NM_017780.3 | c.2708A>C (p.His903Pro) | Missense | - | - | VUS (PM2, PP2, PP3) | 5139 | PMID: 30733481* |
|  | NM_017780.3 | c.3036G>C (p.Leu1012Phe) | Missense | - | - | VUS (PM2, PP2) | 7355 | - |
|  | NM_017780.3 | c.3245C>T (p.Thr1082Ile) | Missense | - | - | LP (PM2, PM5, PP2, PP3) | 7081 | PMID: 30733481* |
|  | NM_017780.3 | c.4354G>T (p.Val1452Leu) | Missense | - | - | VUS (PM2, PP2, PP3) | 5186 | PMID: 30733481* |
|  | NM_017780.3 | c.5561A>G (p.Asp1854Gly) | Missense | - | - | VUS (PM2, PP2) | 5136 | PMID: 30733481* |
|  | NM_017780.3 | c.6194G>A (p.Arg2065His) | Missense | - | - | P (PS4, PM2, PM5, PP2, PP3) | 5164 | PMID: 21158681 PMID: 30733481* |
|  | NM_017780.3 | c.7579A>C (p.Met2527Leu) | Missense | 0.002038 | 0.002 | LB (PP2, BS1, BS2, BP6) | 5102, 8474 | PMID: 25077900 |
| *CHL1* | NM_006614.2 | c.1504G>A (p.Glu502Lys) | Missense | 0.000620 | - | LB (PM2, BP4, BP6) | 5679 | - |
|  | NM_006614.2 | c.3153G>C (p.Glu1051Asp) | Missense | 0.000882 | 0.008 | LB (PM2, BP4, BP6) | 5102 | - |
| *CNTN2* | NM_005076.3 | c.505C>T (p.Leu169Phe) | Missense | 0.002543 | - | LB (PP2, BS1, BS2, BP4, BP6) | 5164 | - |
|  | NM_005076.3 | c.1975A>G (p.Asn659Asp) | Missense | 0.008071 | 0.004 | B (PP2, BA1, BS2, BP4, BP6) | 6178 | - |
| *CPE* | NM_001873.3 | c.884C>G (p.Pro295Arg) | Missense | - | - | VUS (PM2, BP4) | 8299 | - |
| *DCAF17* | NM_025000.3 | c.552_554delACA (p.Gln184del) | Inframe deletion | 0.000016 | - | VUS (PM2, PM4) | 8214 | - |
| *DCC* | NM_005215.3 | c.527A>G (p.Asn176Ser) | Missense | 0.000124 | 0.002 | VUS | 7367 | PMID: 24808016 |
|  | NM_005215.3 | c.1256A>G (p.Lys419Arg) | Missense | 0.002868 | 0.004 | LB (BS1, BP4, BP6) | 7625 | - |
|  | NM_005215.3 | c.1409G>A (p.Gly470Asp) | Missense | 0.002885 | 0.002 | B (BA1, BS2, BP6) | 7075 | PMID: 24808016 |
|  | NM_005215.3 | c.2708dupT (p.Ser904Lysfs*14) | Frameshift | - | - | LP (PVS1, PM2) | 6130 | - |
| *DLG2* | NM_001142699.1 | c.1346C>T (p.Pro449Leu) | Missense | 0.000735 | - | VUS (PM2) | 6889 | - |
|  | NM_001142699.1 | c.1541T>C (p.Leu514Pro) | Missense | 0.000946 | 0.004 | LB (PM2, BS2, BP6) | 6178 | - |
| *DMXL2* | NM_001174116.3 | c.23C>G (p.Thr8Ser) | Missense | 0.004346 | 0.002 | B (PP2, BA1, BS1, BS2, BP4, BP6) | 6263 | - |
|  | NM_001174116.3 | c.1330A>G (p.Met444Val) | Missense | 0.000004 | - | VUS (PM2, PP2, BP4) | 6130 | - |
|  | NM_001174116.3 | c.2069G>A (p.Ser690Asn) | Missense | - | - | VUS (PM2, PP2, BP4) | 7183 | - |
|  | NM_001174116.3 | c.4334T>C (p.Ile1445Thr) | Missense | 0.002499 | - | B (PP2, BA1, BS2, BP4, BP6) | 5195 | - |
|  | NM_001174116.3 | c.4442A>G (p.Asp1481Gly) | Missense | 0.009030 | 0.006 | B (PP2, BA1, BS2, BP6) | 5070 | - |
| *DUSP6* | NM_001946.2 | c.1037C>T (p.Thr346Met) | Missense | 0.000286 | 0.002 | VUS (PM2, BP6) | 5182 | PMID: 23643382 |
| *EDNRB* | NM_000115.4 | c.167A>C (p.Lys56Thr) | Missense | 0.000029 | 0.002 | VUS (PM2) | 6263 | PMID: 20009762 |
| *EGF* | NM_001963.5 | c.531G>C (p.Glu177Asp) | Missense | - | - | VUS (PM2) | 7030 | - |
|  | NM_001963.5 | c.1478T>C (p.Ile493Thr) | Missense | - | - | VUS (PM2, PP3) | 8297 | PMID: 19319977 |
|  | NM_001963.5 | c.1481G>A (p.Arg494Gln) | Missense | 0.000028 | - | VUS (PM2, PP3) | 5139 | - |
|  | NM_001963.5 | c.1789A>G (p.Ile597Val) | Missense | 0.001118 | 0.002 | B (BS2, BP6) | 2661 | - |
|  | NM_001963.5 | c.2378A>T (p.Glu793Val) | Missense | 0.000074 | - | VUS (PM2) | 5236 | - |
|  | NM_001963.5 | c.2827C>T (p.Arg943Cys) | Missense | 0.000057 | - | VUS (PM2, BP4) | 6024 | - |
|  | NM_001963.5 | c.2943T>A (p.Asp981Glu) | Missense | 0.000117 | - | VUS (PM2, BP4) | 6024 | - |
|  | NM_001963.5 | c.3068G>A (p.Arg1023His) | Missense | 0.000012 | - | VUS (PM2, BP4) | 6178 | - |
| *EGFR* | NM_005228.3 | c.1580G>A (p.Arg527Gln) | Missense | 0.000141 | - | LB (BS1, BS2, BP4, BP6) | 8347 | - |
| *EPHA5* | NM_004439.5 | c.1276G>A (p.Val426Ile) | Missense | 0.000804 | - | VUS (PM2) | 8255 | - |
|  | NM_004439.5 | c.1480C>T (p.Arg494Cys) | Missense | 0.000199 | 0.006 | VUS (PM2) | 2661, 5136 | PMID: 27582484 |
|  | NM_004439.5 | c.1690G>A (p.Ala564Thr) | Missense | 0.000052 | - | VUS (PM2, BS2) | 5190 | - |
| *FEZ1* | NM_005103.5 | c.960C>G (p.Ile320Met) | Missense | 0.000085 | - | VUS (PM2, BP4) | 8214 | - |
| *FEZF1* | NM_001024613.3 | c.253A>G (p.Ser85Gly) | Missense | 0.005837 | - | B (BS2, BP4, BP6) | 2661 | PMID: 26204995 |
|  | NM_001024613.3 | c.869G>C (p.Cys290Ser) | Missense | - | - | VUS (PM2, PP1, PP3) | 5404 | - |
|  | NM_001024613.3 | c.1397T>G (p.Leu466Arg) | Missense | 0.000061 | - | LB (PM2, PP1, BS2, BP4) | 5404 | - |
| *FGFR1* | NM_023110.2 | c.12G>T (p.Trp4Cys) | Missense | - | - | VUS (PM2, PP2) | 7081 | PMID: 26277103* |
|  | NM_023110.2 | c.95dupA (p.Pro33Alafs*17) | Frameshift | - | - | LP (PVS1, PM2) | 5184 | PMID: 26277103* |
|  | NM_023110.2 | c.242T>C (p. Ile81Thr) | Missense | - | - | VUS (PM2, PP2) | 7367 | PMID: 35457241* |
|  | NM_023110.2 | c.287C>G (p.Ser96Cys) | Missense | - | - | LP (PM1, PM2, PP2, PP3, PP5) | 5174 | PMID: 26277103* |
|  | NM_023110.2 | c.677_678delGCinsAA (p.Gly226Glu) | Indel | - | - | LP (PM1, PM2, PM5, PP2) | 8325 | - |
|  | NM_023110.2 | c.1961dupA (p.Tyr654*) | Frameshift | - | - | LP (PVS1, PM2) | 3329 | PMID: 26277103* |
|  | NM_023110.2 | c.2155A>G (p.Met719Val) | Missense | - | - | P (PS4, PM1, PM2, PM5, PP2, PP3) | 2520 | PMID: 26277103* |
| *FSHB* | NM_000510.2 | c.59G>T (p.Ser20Ile) | Missense | 0.002118 | 0.006 | LB (PM2, BS2, BP6) | 5182 | PMID: 32242295 |
|  | NM_000510.2 | c.177C>A (p.Asp59Glu) | Missense | - | - | VUS (PM2) | 7407 | - |
| *FSTL5* | NM_020116.5 | c.51G>C (p.Glu17Asp) | Missense | 0.002928 | 0.006 | LB (PM2, BS2, BP4) | 6980 | - |
| *GADL1* | NM_207359.3 | c.9C>A (p.Ser3Arg) | Missense | 0.000098 | - | VUS (PM2, BP4) | 5163 | - |
|  | NM_207359.3 | c.620C>T (p.Ser207Leu) | Missense | 0.000805 | 0.002 | LB (PM2, PP1, BS2, BP4) | 5404 | - |
|  | NM_207359.3 | c.1312G>A (p.Ala438Thr) | Missense | 0.004121 | 0.008 | B (BS1, BS2, BP4) | 5185 | - |
| *GHR* | NM_000163.4 | c.484G>A (p.Val162Ile) | Missense | 0.001392 | - | B (BA1, BS2, BP6) | 5404 | PMID: 9814495 |
|  | NM_000163.4 | c.686G>A (p.Arg229His) | Missense | 0.001213 | - | B (PM5, PP3, BS1, BS2, BP6) | 6776 | PMID: 7565946 |
|  | NM_000163.4 | c.1156C>T (p.Arg386Cys) | Missense | 0.000336 | 0.002 | VUS (BS4) | 5163 | PMID: 18303074 |
| *GJB2* | NM_004004.6 | c.101T>C (p.Met34Thr) | Missense | 0.008996 | 0.008 | LP ( PM1, PM2, PM5, PP1, PP2, PP3, PP5) | 8302 | PMID: 9139825 |
|  | NM_004004.6 | c.284T>C (p.Val95Ala) | Missense | 0.000004 | - | LP (PM1, PM2, PM5, PP2, PP3, PP5) | 7355 | - |
|  | NM_004004.6 | c.358_360delGAG (p.Glu120del) | Inframe deletion | 0.000071 | - | P (PS3, PM2, PM3, PM4) | 5404 | PMID: 29501291 |
|  | NM_004004.6 | 478G>A (p.Gly160Ser) | Missense | 0.000602 | 0.002 | LB (PP2, PP3, BS1, BP6) | 5102 | PMID: 12189487 |
| *GLI2* | NM_005270.4 | c.47A>G (p.Lys16Arg) | Missense | - | - | VUS (PM2) | 5186 | PMID: 23788652 |
|  | NM_005270.4 | c.968T>C (p.Phe323Ser) | Missense | 0.000024 | - | VUS (PM2) | 5182 | - |
|  | NM_005270.4 | c.2159G>A (p.Arg720His) | Missense | 0.000429 | 0.004 | LB (BS1, BP6) | 8214 | PMID: 25327282 |
|  | NM_005270.4 | c.2998C>T (p.Pro1000Ser) | Missense | - | - | VUS (PM2, BP4) | 5943 | - |
|  | NM_005270.4 | c.3348C>G (p.Asn1116Lys) | Missense | 0.000079 | - | LB (PM2, BP4, BP6) | 5196, 8297 | - |
|  | NM_005270.4 | c.3590G>A (p.Gly1197Asp) | Missense | 0.001640 | - | B (BA1, BS2, BP4, BP6) | 8347 | PMID: 22967285 |
| *GLI3* | NM_000168.5 | c.233C>T (p.Ser78Leu) | Missense | 0.000008 | - | VUS (PM2, BP6) | 6889 | - |
|  | NM_000168.5 | c.3208A>T (p.Thr1070Ser) | Missense | 0.000835 | - | B (BS1, BP4, BP6) | 7506 | - |
|  | NM_000168.5 | c.3664C>T (p.Pro1222Ser) | Missense | 0.002656 | 0.004 | B (BP4, BS1, BS2, BP6) | 7081 | - |
| *GNRH1* | NM_000825.3 | c.99delA (p.Leu34Cysfs*12) | Frameshift | - | - | LP (PVS1, PM2) | 8255 | PMID: 19567835 |
| *GNRHR* | NM_000406.2 | c.317A>G (p.Gln106Arg) | Missense | 0.002749 | 0.004 | LP (PM1, PP2, PM2, PP5) | 7355, 6980, 8299, 8302 | PMID: 9371856 |
|  | NM_000406.2 | c.401T>G (p.Val134Gly) | Missense | 0.000028 | - | P (PS3, PM1, PM2, PM3, PP2, PP3) | 5196, 7363 | PMID: 25016926 PMID: 28611058* |
|  | NM_000406.2 | c.410T>C (p.Leu137Pro) | Missense | - | - | LP (PM1, PM2, PM3, PP2) | 8500 | - |
|  | NM_000406.2 | c.415C>T (p.Arg139Cys) | Missense | 0.000012 | - | P (PM1, PM2, PM3, PM5, PP2, PP3) | 7363 | PMID: 17179725 PMID: 28611058* |
|  | NM_000406.2 | c.785G>A (p.Arg262Gln) | Missense | 0.001789 | - | P (PM1, PM2, PM3, PM5, PP2, PP3)) | 7407 , 8299 | PMID: 9371856 |
|  | NM_000406.2 | c.847T>C (p.Tyr283His) | Missense | 0.000008 | - | LP (PM1, PM2, PP1, PP2, PP3) | 6263 | PMID: 28611058* |
|  | NM_000406.2 | c.937_947delTTTTTAAACCC (p.Phe313Metfs*3) | Frameshift | - | - | LP (PVS1, PM2, PP1) | 7407, 6980 | PMID: 28611058* |
| *HJV* | NM_213653.3 | c.904G>A (p.Glu302Lys) | Missense | 0.000290 | - | VUS (PP2) | 7390 | PMID: 15254010 |
| *IFT172* | NM_015662.2 | c.806T>A (p.Ile269Asn) | Missense | - | - | VUS (PM2, BP4) | 7506 | - |
|  | NM_015662.2 | c.1678A>G (p.Met560Val) | Missense | 0.000178 | - | LB (BS1, BP4, BP6) | 6024 | - |
|  | NM_015662.2 | c.3196C>T (p.Arg1066Trp) | Missense | 0.000024 | - | VUS (PM2) | 7506 | - |
|  | NM_015662.2 | c.4130C>T (p.Ala1377Val) | Missense | 0.000106 | - | VUS (PM2, PP3) | 5140 | - |
| *IGSF1* | NM_001170961.1 | c.3119G>A (p.Arg1040His) | Missense | 0.000141 | - | LB (PM2, BS2, BP6) | 7628 | - |
|  | NM_001170961.1 | c.3243G>C (p.Met1081Ile) | Missense | 0.003153 | 0.007 | B (BA1, BS2, BP4, BP6) | 8500 | - |
| *IGSF10* | NM_178822.4 | c.353G>A (p.Arg118Gln) | Missense | 0.000878 | 0.002 | LB (PM2, BS2, BP4, BP6) | 7379 | PMID: 28324054 |
|  | NM_178822.4 | c.467G>T (p.Arg156Leu) | Missense | 0.000354 | 0.002 | VUS (PM2, PP5) | 7030 | PMID: 27137492 |
|  | NM_178822.4 | c.2610G>A (p.Met870Ile) | Missense | 0.000021 | - | VUS (PM2, BP4) | 8474 | - |
|  | NM_178822.4 | c.3574A>G (p.Ile1192Val) | Missense | 0.000814 | - | LB (PM2, BP4, BP6) | 7407 | - |
|  | NM_178822.4 | c.3712A>G (p.Thr1238Ala) | Missense | 0.006200 | 0.002 | B (PM2, BS2, BP4, BP6) | 5070 | - |
|  | NM_178822.4 | c.3797C>G (p.Thr1266Ser) | Missense | 0.001642 | 0.004 | LB (PM2, BP4, BP6) | 7013 | - |
|  | NM_178822.4 | c.3856A>G (p.Lys1286Glu) | Missense | 0.000693 | 0.002 | LB (PM2, BP4, BP6) | 8347 | - |
|  | NM_178822.4 | c.4187C>T (p.Ser1396Phe) | Missense | 0.000233 | 0.006 | VUS (PM2, BP4) | 6889, 7363 | PMID: 31726455 |
|  | NM_178822.4 | c.4564G>A (p.Val1522Ile) | Missense | 0.002844 | - | B (BS1, BS2, BP4, BP6) | 8347 | - |
|  | NM_178822.4 | c.5405A>T (p.Asp1802Val) | Missense | 0.002789 | - | B (PM2, BS2, BP6) | 7391 | - |
|  | NM_178822.4 | c.5983G>A (p.Val1995Ile) | Missense | 0.000772 | 0.002 | VUS (PM2, BS2) | 8474 | PMID: 31726455 |
|  | NM_178822.4 | c.7350_7353dupATCA (p.Leu2452Ilefs*7) | Frameshift | 0.001380 | - | LB (PM2, BS2, BP6) | 7081, 5915 | PMID: 27137492 |
| *IL17RD* | NM_017563.4 | c.392A>C (p.Lys131Thr) | Missense | 0.000856 | 0.002 | VUS (PM2) | 5174 | PMID: 23643382 |
|  | NM_017563.4 | c.1697C>T (p.Pro566Leu) | Missense | 0.002099 | - | B (BS1, BS2, BP6) | 5102 | - |
|  | NM_017563.4 | c.2158T>C (p.Cys720Arg) | Missense | - | - | VUS (PM2) | 5161 | - |
| *JAG1* | NM_000214.2 | c.5G>T (p.Arg2Leu) | Missense | 0.000045 | 0.002 | VUS (PM2, PP2, BP4) | 6319 | - |
| *KIF14* | NM_014875.2 | c.1087G>A (p.Val363Ile) | Missense | 0.002968 | 0.006 | B (BS1, BS2, BP6) | 5161 | - |
|  | NM_014875.2 | c.1685G>A (p.Gly562Asp) | Missense | 0.000004 | - | VUS (PM2) | 7407 | - |
|  | NM_014875.2 | c.2648G>A (p.Arg883His) | Missense | 0.000064 | 0.002 | VUS (PM2, BP4) | 5187, 6263 | - |
| *KLB* | NM_175737.3 | c.1015G>A (p.Asp339Asn) | Missense | 0.001570 | - | B (BS1, BS2, BP6) | 8347 | - |
|  | NM_175737.3 | c.3124G>A (p.Val1042Ile) | Missense | 0.003275 | 0.002 | LB (PM2, BS2, BP4, BP6) | 5192, 8325 | PMID: 32870266 |
| *LEP* | NM_000230.2 | c.397G>A (p.Gly133Ser) | Missense | - | - | VUS (PM2, BP4) | 8214 | - |
| *LHX3* | NM_014564.4 | c.38C>T (p.Ser13Leu) | Missense | 0.000017 | - | VUS (PM2) | 5140, 6130 | - |
|  | NM_014564.4 | c.964G>T (p.Val322Phe) | Missense | - | - | VUS (PM2, BP4) | 7386 | - |
| *MAGEL2* | NM_019066.5 | c.1286C>T (p.Pro429Leu) | Missense | 0.002873 | 0.006 | B (BS1, BS2, BP6) | 7612 | - |
|  | NM_019066.5 | c.2028G>T (p.Glu676Asp) | Missense | 0.000016 | 0.002 | VUS (PM2) | 7359 | - |
|  | NM_019066.5 | c.2074G>A (p.Val692Ile ) | Missense | 0.003543 | 0.002 | B (BA1, BS2, BP6) | 5102 | - |
|  | NM_019066.5 | c.2330C>T (p.Pro777Leu) | Missense | - | - | VUS (PM2) | 5183 | - |
|  | NM_019066.5 | c.3017C>G (p.Thr1006Ser) | Missense | 0.003454 | 0.002 | B (BA1, BS2, BP6) | 6024, 7095 | - |
| *MASTL* | NM_032844.5 | c.247G>T (p.Asp83Tyr) | Missense | 0.000020 | - | VUS (PM2) | 5189 | - |
|  | NM_032844.5 | c.1156G>A (p.Val386Ile) | Missense | 0.000008 | - | VUS (PM2, BP4) | 5136 | - |
|  | NM_032844.5 | c.1415A>G (p.Glu472Gly) | Missense | 0.000702 | - | LB (PM2, BS2, BP4, BP6) | 8347 | - |
|  | NM_032844.5 | c.1774A>G (p.Arg592Gly) | Missense | 0.000008 | - | VUS (PM2, BP4) | 8299 | - |
|  | NM_032844.5 | c.1828G>A (p.Val610Ile) | Missense | 0.009365 | 0.008 | B (BS2, BP4, BP6) | 8255 | - |
|  | NM_032844.5 | c.2620G>A (p.Val874Ile) | Missense | 0.004148 | - | B (BS2, BP4, BP6) | 5185 | - |
| *MC4R* | NM_005912.2 | c.751A>C (p.Ile251Leu) | Missense | 0.006936 | - | B (PP2, BS1, BS2, BP4, BP6) | 5191, 7374 | PMID: 12646666 |
| *MET* | NM_001127500.1 | c.3029C>T (p.Thr1010Ile) | Missense | 0.008249 | 0.004 | B (BA1, BS2, BP6) | 5140, 5190 | PMID: 21970370 |
| *MKKS* | NM_018848.3 | c.724G>T (p.Ala242Ser) | Missense | 0.005251 | 0.006 | B (PP3, BA1, BS2, BP6) | 5943, 7183 | PMID:10802661 |
|  | NM_018848.3 | c.1462G>A (p.Ala488Thr) | Missense | 0.003649 | - | B (BA1, BS2, BP4, BP6) | 7030 | PMID:20472660 |
| *MTOR* | NM_004958.3 | c.4128T>G (p.Asp1376Glu) | Missense | 0.000004 | - | VUS (PM2, PP2, BP6) | 5163 | PMID: 27830187 |
|  | NM_004958.3 | c.5350C>T (p.Arg1784Cys) | Missense | 0.000025 | - | VUS (PM2, PP2, BP6) | 5163 | - |
|  | NM_004958.3 | c.5501C>T (p.Thr1834Met) | Missense | 0.000358 | - | LB (PP2, BP4, BP6) | 5404 | - |
| *NDNF* | NM_024574.3 | c.1624C>A (p.Leu542Met) | Missense | 0.000012 | - | VUS (PM2, BP4) | 5136 | - |
| *NEUROG3* | NM_020999.4 | c.46G>C (p.Glu16Gln) | Missense | 0.000432 | 0.002 | LB (PM2, BS2, BP6) | 7030 | *-* |
| *NOS1* | NM_000620.4 | c.335C>T (p.Thr112Ile) | Missense | 0.000161 | - | VUS (PM2, PP2, BP4) | 7625 | - |
|  | NM_000620.4 | c.458C>T (p.Ser153Leu) | Missense | 0.002062 | 0.002 | B (PP2, BS1, BS2, BP4, BP6) | 8347 | - |
|  | NM_000620.4 | c.2591G>A (p.Gly864Asp) | Missense | 0.004400 | - | B (PM2, BS2, PP2, BP4, , BP6) | 7013 | - |
| *NOTCH1* | NM_017617.4 | c.64C>T (p.Pro22Ser) | Missense | 0.002126 | 0.006 | B (PP2, BA1, BS2, BP4, BP6) | 7625 | - |
|  | NM_017617.4 | c.4699G>A (p.Glu1567Lys) | Missense | - | - | VUS (PM2, PP2) | 5404 | - |
|  | NM_017617.4 | c.5011G>A (p.Val1671Ile) | Missense | 0.003530 | 0.006 | B (PP2, BA1, BS2, BP4, BP6) | 7625 | PMID: 29332214 |
|  | NM_017617.4 | c.5837G>A (p.Arg1946His) | Missense | 0.000024 | - | VUS (PM2, PP2, BP6) | 5174 | - |
|  | NM_017617.4 | c.6119A>G (p.Asn2040Ser) | Missense | 0.000004 | - | VUS (PM2, PP2) | 8325 | PMID: 29177441 |
|  | NM_017617.4 | c.6365C>T (p.Pro2122Leu) | Missense | 0.000008 | - | VUS (PM2, PP2, BP6) | 2520 | PMID: 28649221 |
|  | NM_017617.4 | c.6949G>A (p.Gly2317Ser) | Missense | 0.000020 | - | LB (PM2, PP2, BP4, BP6) | 6263 | - |
| *NRP2* | NM_201266.1 | c.1000C>T (p.Arg334Cys) | Missense | 0.001622 | - | VUS (PM2, BS2, PP3) | 8214 | PMID: 33212964 |
|  | NM_201266.1 | c.1909G>A (p.Asp637Asn) | Missense | - | - | VUS (PM2) | 5182 | - |
|  | NM_201266.1 | c.2552C>T (p.Ser851Leu) | Missense | 0.000056 | 0.002 | VUS (PM2, BP4) | 7081 | - |
| *NTN1* | NM_004822.2 | c.1378A>C (p.Thr460Pro) | Missense | 0.000336 | - | LB (PM2, BS2, BP4) | 7390 | - |
|  | NM_004822.2 | c.1466A>G (p.Lys489Arg) | Missense | 0.000145 | 0.002 | VUS (PM2) | 7374 | PMID: 28945198 |
|  | NM_004822.2 | c.1751C>G (p.Thr584Arg) | Missense | 0.000004 | - | VUS (PM2, PP1, BP4) | 5404 | - |
| *OTUD4* | NM_001102653.1 | c.331G>C (p.Val111Leu) | Missense | - | - | VUS (PM2) | 5070 | - |
| *PCSK1* | NM_000439.4 | c.337C>A (p.Leu113Ile) | Missense | - | - | VUS (PM2, BP4) | 5187 | - |
|  | NM_000439.4 | c.1918A>G (p.Thr640Ala) | Missense | 0.001306 | 0.006 | LB (BS1, BP4, BP6) | 5135 | PMID: 29726959 |
| *PDE3A* | NM_000921.4 | c.296A>C (p.Glu99Ala) | Missense | 0.003209 | 0.004 | LB (PM2, BS2, BP4, BP6) | 5192, 5070, 7183 | - |
|  | NM_000921.4 | c.578T>C (p.Val193Ala) | Missense | 0.003176 | 0.004 | LB (PM2, BS2, BP4, BP6) | 7081 | - |
|  | NM_000921.4 | c.1807G>A (p.Glu603Lys) | Missense | 0.000007 | - | VUS (PM2, BP4) | 7335 | - |
| *PHF6* | NM_032458.3 | c.1045A>G (p.Lys349Glu) | Missense | 0.000011 | - | VUS (PM2, BS2, PP2) | 7628 | - |
| *PLXNA1* | NM_032242.3 | c.841A>C (p.Lys281Gln) | Missense | - | - | VUS (PM2, PP2) | 7363 | - |
|  | NM_032242.3 | c.965G>A (p.Arg322His) | Missense | 0.000108 | - | VUS (PM2, PP2, BP4) | 7074 | - |
|  | NM_032242.3 | c.1628G>A (p.Arg543Gln) | Missense | 0.000007 | - | VUS (PM2, PP2) | 8302 | - |
|  | NM_032242.3 | c.2218C>G (p.Arg740Gly) | Missense | 0.000032 | - | VUS (PM2, PP2, BP4) | 6889 | - |
|  | NM_032242.3 | c.2564G>A (p.Arg855His) | Missense | 0.000145 | - | VUS (PM2, PP2, BP4) | 7013 | - |
|  | NM_032242.3 | c.5627C>T (p.Ala1876Val) | Missense | 0.000017 | - | VUS (PM2, PP2) | 6776 | - |
| *PLXNA3* | NM_017514.4 | c.124A>T (p.Thr42Ser) | Missense | 0.000020 | - | VUS (PM2, BS2) | 6178 | - |
|  | NM_017514.4 | c.1357A>T (p.Thr453Ser) | Missense | - | 0.002 | VUS (PM2, BP4) | 5182 | - |
|  | NM_017514.4 | c.1709C>T (p.Ala570Val) | Missense | 0.000046 | - | LB (PM2, BS2, BP4) | 5404 | - |
| *PLXNB1* | NM_002673.4 | c.655G>A (p.Val219Met) | Missense | 0.001526 | 0.006 | VUS (PM2, BS2, PP2) | 7407 | - |
|  | NM_002673.4 | c.1327T>G (p.Ser443Ala) | Missense | 0.000203 | - | VUS (PM2, PP2, BP4) | 5040 | - |
|  | NM_002673.4 | c.2987G>A (p.Arg996His) | Missense | 0.000026 | - | VUS (PM2, PP2, BP4) | 5237 | - |
| *POGZ* | NM_015100.4 | c.2789C>G (Pro930Arg) | Missense | 0.001292 | - | B (PP2, BA1, BS2, BP4, BP6) | 8214 | - |
|  | NM_015100.4 | c.364A>G (p.Thr122Ala) | Missense | - | - | VUS (PM2, PP2, BP4) | 8255 | - |
| *POLA1* | NM_016937.3 | c.1555A>G (p.Met519Val) | Missense | 0.000073 | 0.002 | B (PM2, BS2, BP4, BP6) | 5943 | - |
|  | NM_016937.3 | c.2267A>G (p.Lys756Arg) | Missense | 0.003633 | - | B (BA1, BS2, BP4, BP6) | 5140-het; 2594-homo | - |
|  | NM_016937.3 | c.3981C>A (p.Ser1327Arg) | Missense | 0.000018 | - | LB (PM2, BS2, BP4) | 5182 | - |
| *POLR3A* | NM_007055.3 | c.1177C>T (p.Pro393Ser) | Missense | 0.000004 | 0.002 | VUS (PM2, PP2, PP3) | 7335 | - |
| *POLR3B* | NM_018082.5 | c.1568T>A (p.Val523Glu) | Missense | 0.000290 | - | P (PM2, PM3, PP1, PP2, PP3) | 6776 | PMID: 22036172 |
| *POU6F2* | NM_007252.4 | c.587_589dupAGC (p.Gln196dup) | Inframe insertion | 0.001682 | - | VUS (PM2, PM4) | 5915 | *-* |
|  | NM_007252.4 | c.1340C>G (p.Ala447Gly) | Missense | 0.000054 | - | VUS (PM2) | 8347 | *-* |
| *PROK2* | NM_001126128.1 | c.163delA (p.Ile55*) | Frameshift | 0.000113 | - | P (PVS1, PS4, PM2) | 7075 | PMID: 17959774 |
|  | NM_001126128.1 | c.297dupT (p.Gly100Trpfs*22) | Frameshift | 0.000110 | - | LP (PVS1, PM2) | 8276 | PMID: 17054399 |
| *PROKR2* | NM_144773.3 | c.238C>T (p.Arg80Cys) | Missense | 0.000008 | - | VUS (PM1, PM2) | 5196 | PMID: 18682503 PMID: 28611058* |
|  | NM_144773.3 | c.253C>T (p.Arg85Cys) | Missense | 0.000601 | 0.002 | LP (PM1, PM2, PM5, PP5) | 7390 | PMID: 17054399 |
|  | NM_144773.3 | c.518T>G (p.Leu173Arg) | Missense | 0.002196 | 0.004 | LB (BS2, BP6) | 5070 | PMID: 17054399 |
|  | NM_144773.3 | c.528G>C (p.Leu176Phe) | Missense | 0.000004 | - | VUS (PM2, BP4) | 5070 | PMID: 30733481* |
| *PTCH1* | NM_000264.3 | c.1306G>A (p.Asp436Asn) | Missense | 0.000704 | 0.002 | LB (PP2, BS1, BP6) | 7381 | PMID: 16231297 |
|  | NM_000264.3 | c.2173C>T (p.Pro725Ser) | Missense | 0.000891 | 0.002 | B (PP2, BS1, BP6) | 7381, 7013 | PMID: 28733979 |
|  | NM_000264.3 | c.3487G>A (p.Gly1163Ser) | Missense | 0.000513 | 0.002 | VUS (PM5, PP2, PP3, BP6) | 6776, 7359 | PMID: 25260786 |
|  | NM_000264.3 | c.3883C>T (p.Pro1295Ser) | Missense | - | - | VUS (PM2, PP2) | 7031 | - |
|  | NM_000264.3 | c.4031C>T (p.Ala1344Val) | Missense | - | - | VUS (PM2, PP2) | 5183 | - |
|  | NM_000264.3 | c.4219G>A (p.Gly1407Ser) | Missense | 0.000110 | - | LB (PM2, PP2, BS2, BP6) | 5196 | PMID: 26590974 |
| *RAB3GAP1* | NM_012233.3 | c.244G>A (p.Glu82Lys) | Missense | 0.000004 | 0.002 | VUS (PM2, BP4) | 8299 | - |
|  | NM_012233.3 | c.1006C>T (p.Arg336Cys) | Missense | 0.004542 | 0.006 | B (BA1, BS2, BP6) | 7081, 8325 | PMID: 29924831 |
|  | NM_012233.3 | c.2800C>G (p.Pro934Ala) | Missense | 0.000909 | - | B (BA1, BS2, BP6) | 8276 | - |
| *RAB3GAP2* | NM_012414.3 | c.745C>T (p.Pro249Ser) | Missense | 0.000004 | - | VUS (PM2, PP1) | 6980 | - |
|  | NM_012414.3 | c.1346T>A (p.Phe449Tyr) | Missense | - | - | VUS (PM2, BP4) | 5194 | - |
|  | NM_012414.3 | c.2290C>T (p.Leu764Phe) | Missense | 0.000991 | - | LB (BS1, BP4) | 6889 | - |
|  | NM_012414.3 | c.3058T>A (p.Tyr1020Asn) | Missense | 0.000032 | - | VUS (PM2) | 7355 | - |
| *RBM28* | NM_018077.2 | c.280A>T (p.Asn94Tyr) | Missense | 0.000168 | - | LB (PM2, BP4, BP6) | 7381 | - |
|  | NM_018077.2 | c.1744_1745insT (p.Arg582Leufs*5) | Frameshift | - | - | LP (PVS1, PM2) | 8299 | - |
|  | NM_018077.2 | c.2077C>T (p.His693Tyr) | Missense | 0.002121 | 0.008 | LB (PM2, BS2, BP4, BP6) | 5185 | - |
| *RELN* | NM_005045.3 | c.877G>A (p.Asp293Asn) | Missense | 0.000425 | - | LB (PM2, PP2, BP4, BP6) | 5194 | PMID: 30619712 |
|  | NM_005045.3 | c.4279T>C (p.Cys1427Arg) | Missense | - | - | VUS (PM2, PP2, PP3) | 7506 | - |
|  | NM_005045.3 | c.4408G>A (p.Val1470Ile) | Missense | 0.002425 | 0.006 | B (PP2, BS1, BS2, BP4, BP6) | 7367 | - |
|  | NM_005045.3 | c.5468C>A (p.Ala1823Glu) | Missense | - | - | VUS (PM2, PP2) | 5163 | - |
|  | NM_005045.3 | c.6170T>G (p.Leu2057Arg) | Missense | 0.000081 | - | VUS (PM2, PP2) | 5186, 6263 | PMID: 26934580 |
|  | NM_005045.3 | c.6214G>A (p.Glu2072Lys) | Missense | 0.000046 | - | LB (PM2, PP2, BS2, BP6) | 7183 | PMID: 26934580 |
|  | NM_005045.3 | c.6749A>G (p.Tyr2250Cys) | Missense | - | - | VUS (PM2, PP2) | 7625 | - |
|  | NM_005045.3 | c.7634C>T (p.Ala2545Val) | Missense | 0.000216 | 0.004 | VUS (PP2, BP6) | 7349, 7506 | PMID: 29969175 |
| *ROBO3* | NM_022370.3 | c.1189C>A (p.Pro397Thr) | Missense | 0.001725 | - | B (BA1, BS2, BP6) | 5943 | - |
|  | NM_022370.3 | c.1655C>G (p.Pro552Arg) | Missense | 0.000036 | - | VUS (PM2) | 7612 | - |
|  | NM_022370.3 | c.2763G>C (p.Glu921Asp) | Missense | 0.001301 | 0.002 | B (BA1, BP6) | 5070 | PMID: 30098700 |
|  | NM_022370.3 | c.2899C>T (p.Pro967Ser) | Missense | 0.000128 | 0.002 | VUS (PM2) | 5679 | - |
|  | NM_022370.3 | c.3922G>A (p.Val1308Met) | Missense | 0.000051 | - | LB (PM2, BP4, BP6) | 7031 | - |
| *SEC14L3* | NM_001376914.1 | c.1015C>T (p.Arg339*) | Nonsense | 0.000322 | - | VUS (PM2) | 5268 | - |
| *SEMA3A* | NM_006080.2 | c.229A>G (p.Ile77Val) | Missense | 0.000046 | - | VUS (PM2, BP4) | 5679 | - |
|  | NM_006080.2 | c.458A>G (p.Asn153Ser) | Missense | 0.002292 | - | LB (PM2, BS2, BP4, BP6) | 3329 | PMID: 22927827 |
| *SEMA3E* | NM_012431.2 | c.2102G>T (p.Ser701Ile) | Missense | 0.005035 | - | B (BS1, BS2, BP4, BP6) | 5184 | - |
| *SEMA4D* | NM_006378.4 | c.2182C>T (p.Leu728Phe) | Missense | 0.002207 | - | B (BS1, BS2, BP6) | 8500 | - |
| *SEMA7A* | NM_003612.3 | c.290T>A (p.Leu97His) | Missense | 0.001274 | 0.002 | VUS (PM2, BP4) | 5196 | - |
|  | NM_003612.3 | c.1865G>A (p.Arg622His) | Missense | 0.000404 | 0.002 | VUS (PM2, BP4) | 5186 | - |
| *SIN3A* | NM_00145358.2 | c.2252A>G (p.Asn751Ser) | Missense | 0.000096 | - | VUS (PM2, PP2) | 7075 | - |
|  | NM_00145358.2 | c.2519A>G (p.Glu840Gly) | Missense | 0.000048 | - | VUS (PM2, PP2, BP4) | 5194 | - |
| *SLC29A3* | NM_018344.5 | c.59C>G (p.Thr20Arg) | Missense | 0.000016 | - | VUS (PM2, BP4) | 8474 | - |
|  | NM_018344.5 | c.128T>G (p.Leu43Arg) | Missense | 0.000697 | - | B (BA1, BS2, BP4, BP6) | 5404 | - |
|  | NM_018344.5 | c.946T>G (p.Phe316Val) | Missense | 0.000768 | - | LB (BS1, BS2) | 5161 | - |
| *SLIT2* | NM_004787.3 | c.1395C>A (p.Asn465Lys) | Missense | 0.000004 | - | VUS (PM2, PP2) | 5404 | - |
|  | NM_004787.3 | c.2372C>T (p.Thr791Met) | Missense | 0.000018 | - | VUS (PM2, PP2) | 6263 | - |
|  | NM_004787.3 | c.4049G>A (p.Ser1350Asn) | Missense | 0.003739 | 0.006 | B (PM2, PP2, BS2, BP6) | 2594 | - |
| *SOX10* | NM_006941.3 | c.191A>T (p.Asp64Val) | Missense | 0.000021 | - | VUS (PM2, PP1) | 3329 | PMID: 29419413 |
|  | NM_006941.3 | c.778G>A (p.Gly260Arg) | Missense | 0.000022 | - | VUS (PM2, PP3) | 8474 | - |
| *SOX11* | NM_003108.4 | c.650T>C (p.Val217Ala) | Missense | 0.000005 | - | VUS (PM2, PP2) | 8276 | - |
| *SPRED3* | NM_001042522.2 | c.360_363dup (p.Ser122Leufs*299) | Frameshift | 0.000411 | - | VUS (PM2) | 2520 | - |
| *SRA1* | NM_001035235.2 | c.236C>T (p.Pro79Leu) | Missense | - | - | VUS (PM2) | 8347 | - |
|  | NM_001035235.2 | c.413G>A (p.Gly138Glu) | Missense | 0.002195 | 0.004 | LB (PM2, BS2, BP4, BP6) | 5164 | - |
| *TACR3* | NM_001059.2 | c.1345G>A (p.Ala449Thr) | Missense | 0.005288 | 0.006 | B (BA1, BS2, BP4, BP6) | 5040 | PMID: 21209029 |
| *TBCE* | NM_003193.3 | c.214C>T (p.Pro72Ser) | Missense | 0.003571 | 0.004 | B (BA1, BS1, BS2, BP6) | 5161, 7625 | - |
|  | NM_003193.3 | c.830C>T (p.Pro277Leu) | Missense | 0.000002 | - | VUS (PM2) | 7625 | - |
| *TBX3* | NM_005996.3 | c.1122G>C (p.Lys374Asn) | Missense | - | - | VUS (PM2) | 7367 | - |
| *TFR2* | NM_003227.3 | c.840C>G (p.Phe280Leu) | Missense | 0.000397 | 0.002 | VUS | 5185, 6024 | PMID: 18762941 |
|  | NM_003227.3 | c.1127C>A (p.Ala376Asp) | Missense | 0.002704 | - | B (BS1, BS2, BP6) | 5190 | PMID: 22890139 |
| *TTC8* | NM_198309.3 | c.254A>G (p.Lys85Arg) | Missense | 0.005035 | - | B (BA1, BS2, BP6) | 8297 | - |
|  | NM_198309.3 | c.278G>T (p.Gly93Val) | Missense | 0.000020 | - | VUS (PM2) | 5189 | - |
| *WDPCP* | NM_015910.7 | c.1333G>C (p.Ala445Pro) | Missense | 0.005543 | - | B (BA1, BS2, BP4, BP6) | 5187 | PMID:26386247 |
|  | NM_015910.7 | c.2063A>G (p.Asn688Ser) | Missense | 0.009322 | - | B (BA1, BS2, BP4, BP6) | 5194, 7335 | - |
| *WDR11* | NM_018117.11 | c.797G>C (p.Arg266Pro) | Missense | 0.000012 | - | LP (PM2, PP3, PP5) | 5040 | - |
|  | NM_018117.11 | c.811C>T (p.Leu271Phe) | Missense | - | - | VUS (PM2) | 7095 | - |
| *WDR4* | NM_033661.4 | c.265C>T (p.Arg89Cys) | Missense | 0.000014 | - | VUS (PM2) | 8299 | - |
|  | NM_033661.4 | c.652T>C (p.Tyr218His) | Missense | 0.000063 | - | VUS (PM2) | 7039 | - |

CHH, Congenital Hypogonadotropic Hypogonadism; GnomAD, Genome Aggregation Database; ACMG, American College of Medical Genetics and Genomics. Variants were classified as Pathogenic (P), Likely Pathogenic (LP), Variants of Uncertain Significance (VUS), Likely Benign (LB), or Benign (B), based on the evidence for pathogenicity [very strong (PVS1), strong (PS1-4), moderate (PM1–6), or supporting (PP1–5)] or benign impact [stand-alone (BA), strong (BS1-4), or supporting (BP1-7)]. id, identification; PMID, PubMed identifier. * Publication by the authors that included the same patient.
