# Supplementary material for: Genetic architecture of congenital hypogonadotropic hypogonadism: insights from analysis of a Portuguese cohort
Source: Hum Reprod Open. 2024 Sep 11;2024(3):hoae053. doi: 10.1093/hropen/hoae053 (PMC11415827; doi:10.1093/hropen/hoae053)
Supplement: hoae053_Supplementary_Data [file hoae053_supplementary_data.zip › Supplementary Table S3.docx]

**Supplementary Table S3.** Characteristics of patients with Pathogenic (P), Likely Pathogenic (LP) and Variants of Uncertain Significance (VUS).

| **Patient id** | **Sex** | **Olfactory status** | **Other associated features** | **Variant** | **Zygosity** | **Allele frequency in GnomAD** | **ACMG classification** | **Previous report** |
| --- | --- | --- | --- | --- | --- | --- | --- | --- |
| 2520 | M | KS | Cryptorchidism, hearing impairment, mental retardation, spina bifida | NM_023110.2(*FGFR1*):c.2155A>G (p.Met719Val) | Het | - | P (PS4, PM1, PM2, PM5, PP2, PP3) | PMID: 26277103* |
|  |  |  |  | NM_017617.4(*NOTCH1*):c.6365C>T (p.Pro2122Leu) | Het | 0.000008 | VUS (PM2, PP2, BP6) | PMID: 28649221 |
|  |  |  |  | NM_001042522.2(*SPRED3*):c.360_363dup (p.Ser122Leufs*299) | Het | 0.000411 | VUS (PM2) | - |
| 2594 | M | KS | Obesity | None |  |  |  |  |
| 2661 | M | nHH |  | NM_004439.5(*EPHA5*):c.1480C>T (p.Arg494Cys) | Het | 0.000199 | VUS (PM2) | PMID: 27582484 |
| 3329 | M | KS | One affected brother | NM_023110.2(*FGFR1*):c.1961dupA (p.Tyr654Ter) | Het | - | LP (PVS1, PM2) | PMID: 26277103* |
|  |  |  |  | NM_006941.3(*SOX10*):c.191A>T (p.Asp64Val) | Het | 0.000021 | VUS (PM2) | PMID: 29419413 |
| 5040 | M | KS |  | NM_024685.4(*BBS10*):c.1837T>C (p.Tyr613His) | Het | 0.000044 | LP (PM1, PM2, PM5, PP3) | PMID: 16582908 |
|  |  |  |  | NM_002673.4(*PLXNB1*):c.1327T>G (p.Ser443Ala) | Het | 0.000203 | VUS (PM2, PP2, BP4) | - |
|  |  |  |  | NM_018117.11(*WDR11*):c.797G>C (p.Arg266Pro) | Het | 0.000012 | LP (PM2, PP3, PP5) | - |
| 5070 | M | nHH | Pituitary hypoplasia | NM_001102653.1(*OTUD4*):c.331G>C (p.Val111Leu) | Het | - | VUS (PM2) | - |
|  |  |  |  | NM_144773.3(*PROKR2*):c.528G>C (p.Leu176Phe) | Het | 0.000004 | VUS (PM2, BP4) | PMID: 30733481* |
| 5102 | M | KS | Unilateral renal agenesis. One affected brother | NM_000216.4(*ANOS1*):c.542-1G>C | Hemi | - | LP (PVS1, PM2) | PMID: 28122887* |
| 5135 | M | KS |  | None |  |  |  |  |
| 5136 | M | KS | Cryptorchidism, hearing impairment | NM_017780.3(*CHD7*):c.5561A>G (p.Asp1854Gly) | Het | - | VUS (PM2, PP2) | PMID: 30733481* |
|  |  |  |  | NM_004439.5(*EPHA5)*:c.1480C>T (p.Arg494Cys) | Het | 0.000199 | VUS (PM2) | PMID: 27582484 |
|  |  |  |  | NM_032844.5(*MASTL*):c.1156G>A (p.Val386Ile) | Het | 0.000008 | VUS (PM2, BP4) | - |
|  |  |  |  | NM_024574.3(*NDNF*):c.1624C>A (p.Leu542Met) | Het | 0.000012 | VUS (PM2, BP4) | - |
| 5139 | M | nHH |  | NM_017780.3(*CHD7*):c.2708A>C (p.His903Pro) | Het | - | VUS (PM2, PP2, PP3) | PMID: 30733481* |
|  |  |  |  | NM_001963.5(*EGF*):c.1481G>A (p.Arg494Gln) | Het | 0.000028 | VUS (PM2, PP3) | - |
| 5140 | F | KS | Mental retardation, hearing impairment, pituitary hypoplasia | NM_015662.2(*IFT172*):c.4130C>T (p.Ala1377Val) | Het | 0.000106 | VUS (PM2, PP3) | - |
|  |  |  |  | NM_014564.4(*LHX3*):c.38C>T (p.Ser13Leu) | Het | 0.000017 | VUS (PM2) | - |
| 5161 | M | nHH |  | NM_017563.4(*IL17RD*):c.2158T>C (p.Cys720Arg) | Het | - | VUS (PM2) | - |
| 5163 | M | KS |  | NM_207359.3(*GADL1*):c.9C>A (p.Ser3Arg) | Het | 0.000100 | VUS (PM2, BP4) | - |
|  |  |  |  | NM_000163.4(*GHR*):c.1156C>T (p.Arg386Cys) | Het | 0.000336 | VUS (BS4) | PMID: 18303074 |
|  |  |  |  | NM_004958.3(*MTOR*):c.4128T>G (p.Asp1376Glu) | Het | 0.000004 | VUS (PM2, PP2, BP6) | PMID: 27830187 |
|  |  |  |  | NM_004958.3(*MTOR*):c.5350C>T (p.Arg1784Cys) | Het | 0.000025 | VUS (PM2, PP2, BP6) | - |
|  |  |  |  | NM_005045.3(*RELN*):c.5468C>A (p.Ala1823Glu) | Het | - | VUS (PM2, PP2) | - |
| 5164 | M | KS | Chronic renal failure- Two affected brothers | NM_017780.3(*CHD7*):c.6194G>A (p.Arg2065His) | Het | - | P (PS4, PM2, PM5, PP2, PP3) | PMID: 21158681 PMID: 30733481* |
| 5174 | M | KS | Cryptorchidism | NM_023110.2(*FGFR1*):c.287C>G (p.Ser96Cys) | Het | - | LP (PM1, PM2, PP2, PP3, PP5) | PMID: 26277103* |
|  |  |  |  | NM_017563.4(*IL17RD*):c.392A>C (p.Lys131Thr) | Het | 0.000856 | VUS (PM2) | PMID: 23643382 |
|  |  |  |  | NM_017617.4(*NOTCH1*):c.5837G>A (p.Arg1946His) | Het | 0.000024 | VUS (PM2, PP2, BP6) | - |
| 5178 | M | KS | Cryptorchidism, pituitary hypoplasia | NM_017780.3(*CHD7*):c.1163G>A (p.Gly388Glu) | Het | 0.000004 | VUS (PM2, PP2) | PMID: 30733481* |
| 5182 | M | nHH |  | NM_001946.2(*DUSP6*):c.1037C>T (p.Thr346Met) | Het | 0.000286 | VUS (PM2, BP6) | PMID: 23643382 |
|  |  |  |  | NM_005270.4(*GLI2*):c.968T>C (p.Phe323Ser) | Het | 0.000024 | VUS (PM2) | - |
|  |  |  |  | NM_201266.1(*NRP2*):c.1909G>A (p.Asp637Asn) | Het | - | VUS (PM2) | - |
|  |  |  |  | NM_017514.4(*PLXNA3*):c.1357A>T (p.Thr453Ser) | Hemi | - | VUS (PM2, BP4) | - |
| 5183 | M | KS | Cryptorchidism | NM_019066.5(*MAGEL2*):c.2330C>T (p.Pro777Leu) | Het | - | VUS (PM2) | - |
|  |  |  |  | NM_000264.3(*PTCH1*):c.4031C>T (p.Ala1344Val) | Het | - | VUS (PM2, PP2) | - |
| 5184 | M | nHH | Cryptorchidism | NM_023110.2(*FGFR1*):c.95dupA (Pro33Alafs*17) | Het | - | LP (PVS1, PM2) | PMID: 26277103* |
| 5185 | M | nHH |  | NM_001080414.4(*CCDC88C*):c.925C>T (p.Arg309Cys) | Het | 0.000100 | VUS (PM2) | - |
|  |  |  |  | NM_003227.3(*TFR2*):c.840C>G (p.Phe280Leu) | Het | 0.000397 | VUS | PMID: 18762941 |
| 5186 | M | KS | Reversal of hypogonadism | NM_017780.3(CHD7):c.4354G>T (p.Val1452Leu) | Het | - | VUS (PM2, PP2, PP3) | PMID: 30733481* |
|  |  |  |  | NM_005270.4(*GLI2*):c.47A>G (p.Lys16Arg) | Het | - | VUS (PM2) | PMID: 23788652 |
|  |  |  |  | NM_005045.3(*RELN*):c.6170T>G (p.Leu2057Arg) | Het | 0.000081 | VUS (PM2, PP2) | PMID: 26934580 |
|  |  |  |  | NM_003612.3(*SEMA7A*):c.1865G>A (p.Arg622His) | Het | 0.000404 | VUS (PM2, BP4) | - |
| 5187 | F | nHH |  | NM_001030055.2(*ARHGAP5*):c.805G>C (p.Val269Leu) | Het | 0.000021 | VUS (PM2, PP2) | - |
|  |  |  |  | NM_024685.4(*BBS10*):c.273C>G (p.Cys91Trp) | Het | 0.000028 | P (PS3, PM2, PM3, PP2) | PMID: 16582908 |
|  |  |  |  | NM_014875.2(*KIF14*):c.2648G>A (p.Arg883His) | Het | 0.000064 | VUS (PM2, BP4) | - |
|  |  |  |  | NM_000439.4(*PCSK1*):c.337C>A (p.Leu113Ile) | Het | - | VUS (PM2, BP4) | - |
| 5189 | M | nHH |  | NM_032844.5(*MASTL*):c.247G>T (p.Asp83Tyr) | Het | 0.000020 | VUS (PM2) | - |
|  |  |  |  | NM_198309.3(*TTC8*):c.278G>T (p.Gly93Val) | Het | 0.000020 | VUS (PM2) | - |
| 5190 | M | nHH | Cryptorchidism, hearing impairment, pituitary hypoplasia | NM_004439.5(*EPHA5*):c.1690G>A (p.Ala564Thr) | Het | 0.000052 | VUS (PM2, BS2) | - |
| 5191 | M | KS | Hearing impairment | NM_000479.3(*AMH*):c.23G>C (p.Ser8Thr) | Het | 0.000143 | VUS | - |
| 5192 | M | nHH |  | None |  |  |  |  |
| 5194 | M | nHH | Mental retardation, epilepsy, Chiari malformation, pituitary hypoplasia | NM_001273.3(*CHD4*):c.1715G>A (p.Arg572Gln) | Het | - | VUS (PM2, PP2, PP3) | PMID: 29844320 |
|  |  |  |  | NM_012414.3(*RAB3GAP2*):c.1346T>A (p.Phe449Tyr) | Het | - | VUS (PM2, BP4) | - |
|  |  |  |  | NM_001145358.2(*SIN3A*):c.2519A>G (p.Glu840Gly) | Het | 0.000048 | VUS (PM2, PP2, BP4) | - |
| 5195 | M | nHH | Cryptorchidism | None |  |  |  |  |
| 5196 | M | nHH |  | NM_000406.2(*GNRHR*):c.401T>G (p.Val134Gly) | Het | 0.000028 | P (PS3, PM1, PM2, PM3, PP2, PP3) | PMID: 25016926 PMID: 28611058* |
|  |  |  |  | NM_144773.3(*PROKR2*):c.238C>T (p.Arg80Cys) | Het | 0.000008 | VUS (PM1, PM2) | PMID: 18682503 PMID: 28611058* |
|  |  |  |  | NM_003612.3(*SEMA7A*):c.290T>A (p.Leu97His) | Het | 0.001274 | VUS (PM2, BP4) | - |
| 5236 | M | nHH |  | NM_001963.5(*EGF*):c.2378A>T (p.Glu793Val) | Het | 0.000074 | VUS (PM2) | - |
| 5237 | M | nHH | Cryptorchidism | NM_004491.5(*ARHGAP35*):c.2495G>A (p.Arg832Gln) | Het | 0.000001 | VUS (PM2, PP2) | - |
|  |  |  |  | NM_002673.4(*PLXNB1*):c.2987G>A (p.Arg996His) | Het | 0.000026 | VUS (PM2, PP2, BP4) | - |
| 5268 | M | KS | Pituitary hypoplasia, short stature | NM_001376914.1(*SEC14L3*):c.1015C>T (p.Arg339*) | Het | 0.000322 | VUS (PM2) | - |
| 5404 | M | nHH | Hirschsprung's disease. One affected brother and one affected sister | NM_001024613.3(*FEZF1*):c.869G>C (p.Cys290Ser) | Het | - | VUS (PM2, PP1, PP3) | - |
|  |  |  |  | NM_004004.6(*GJB2*):c.358_360delGAG (p.Glu120del) | Het | 0.000071 | P (PS3, PM2, PM3, PM4) | PMID: 29501291 |
|  |  |  |  | NM_017617.4(*NOTCH1*):c.4699G>A (p.Glu1567Lys) | Het | - | VUS (PM2, PP2) | - |
|  |  |  |  | NM_004822.2*(NTN1*):c.1751C>G (p.Thr584Arg) | Het | 0.000004 | VUS (PM2, PP1, BP4) | - |
|  |  |  |  | NM_004787.3(*SLIT2*):c.1395C>A (p.Asn465Lys) | Het | 0.000004 | VUS (PM2, PP2) | - |
| 5679 | M | nHH |  | NM_022370.3(*ROBO3*):c.2899C>T (p.Pro967Ser) | Het | 0.000128 | VUS (PM2) | - |
|  |  |  |  | NM_006080.2(*SEMA3A*):c.229A>G (p.Ile77Val) | Het | 0.000046 | VUS (PM2, BP4) | - |
| 5915 | F | nHH | Pituitary hypoplasia | NM_007252.4(*POU6F2*):c.587_589dupAGC (p.Gln196dup) | Het | 0.001682 | VUS (PM2, PM4) | - |
| 5943 | M | nHH | Mental retardation, strabismus, obesity, reversal of hypogonadism | NM_000479.3(*AMH*):c.295A>T (p.Thr99Ser) | Het | 0.000219 | VUS (PM2) | PMID: 28505284 |
|  |  |  |  | NM_005270.4(*GLI2*):c.2998C>T (p.Pro1000Ser) | Het | - | VUS (PM2, BP4) | - |
| 6024 | M | nHH |  | NM_176875.2(*CCKBR*):c.199A>G (p.Met67Val) | Het | - | VUS (PM2, BP4) | - |
|  |  |  |  | NM_001963.5(*EGF*):c.2827C>T (p.Arg943Cys) | Het | 0.000057 | VUS (PM2, BP4) | - |
|  |  |  |  | NM_001963.5(*EGF*):c.2943T>A (p.Asp981Glu) | Het | 0.000117 | VUS (PM2, BP4) | - |
|  |  |  |  | NM_003227.3(*TFR2*):c.840C>G (p.Phe280Leu) | Het | 0.000397 | VUS | PMID: 18762941 |
| 6130 | M | KS | Pituitary hypoplasia | NM_005215.3(*DCC*):c.2708dupT (p.Ser904Lysfs*14) | Het | - | LP (PVS1, PM2) | - |
|  |  |  |  | NM_001174116.2(*DMXL2*):c.1330A>G (p.Met444Val) | Het | - | VUS (PM2, PP2, BP4) | - |
|  |  |  |  | NM_014564.4(*LHX3*):c.38C>T (p.Ser13Leu) | Het | 0.000017 | VUS (PM2) | - |
| 6178 | F | KS |  | NM_001963.5(*EGF*):c.3068G>A (p.Arg1023His) | Het | 0.000012 | VUS (PM2, BP4) | - |
|  |  |  |  | NM_017514.4(*PLXNA3*):c.124A>T (p.Thr42Ser) | Het | 0.000020 | VUS (PM2, BS2) | - |
| 6263 | F | nHH | One affected brother and one affected sister | NM_000115.4(*EDNRB*):c.167A>C (p.Lys56Thr) | Het | 0.000029 | VUS (PM2) | PMID: 20009762 |
|  |  |  |  | NM_000406.2(*GNRHR*):c.847T>C (p.Tyr283His) | Homo | 0.000008 | LP (PM1, PM2, PP1, PP2, PP3) | PMID: 28611058* |
|  |  |  |  | NM_014875.2(*KIF14*):c.2648G>A (p.Arg883His) | Het | 0.000064 | VUS (PM2, BP4) | - |
|  |  |  |  | NM_005045.3(*RELN*):c.6170T>G (p.Leu2057Arg) | Het | 0.000081 | VUS (PM2, PP2) | PMID: 26934580 |
|  |  |  |  | NM_004787.3(*SLIT2*):c.2372C>T (p.Thr791Met) | Het | 0.000018 | VUS (PM2, PP2) | - |
| 6319 | F | KS | Pituitary hypoplasia, short stature, low IGF1 | NM_000214.2(*JAG1*):c.5G>T (p.Arg2Leu) | Het | 0.000045 | VUS (PM2, PP2, BP4) | - |
| 6776 | M | KS | Unilateral renal agenesis, mental retardation, obesity, ichthyosis. One affected brother | NM_000216.4(*ANOS1*):deletion ~4.8 Mb | Hemi | - | P | PMID: 28122887* |
|  |  |  |  | NM_032242.3(*PLXNA1*):c.5627C>T (p.Ala1876Val) | Het | 0.000017 | VUS (PM2, PP2) | - |
|  |  |  |  | NM_018082.5(*POLR3B*):c.1568T>A (p.Val523Glu) | Het | 0.000290 | P (PM2, PM3, PP1, PP2, PP3) | PMID: 22036172 |
|  |  |  |  | NM_000264.3(*PTCH1*):c.3487G>A (p.Gly1163Ser) | Het | 0.000513 | VUS (PM5, PP2, PP3, BP6) | PMID: 25260786 |
| 6889 | M | nHH | Pituitary hypoplasia | NM_001142699.1(*DLG2*):c.1346C>T (p.Pro449Leu) | Het | 0.000735 | VUS (PM2) | - |
|  |  |  |  | NM_000168.5(*GLI3*):c.233C>T (p.Ser78Leu) | Het | 0.000008 | VUS (PM2, BP6) | - |
|  |  |  |  | NM_178822.4(*IGSF10):*c.4187C>T (p.Ser1396Phe) | Het | 0.000200 | VUS (PM2, BP4) | PMID: 31726455 |
|  |  |  |  | NM_032242.3(*PLXNA1*):c.2218C>G (p.Arg740Gly) | Het | 0.000032 | VUS (PM2, PP2, BP4) | - |
| 6980 | M | nHH | One affected sister | NM_001080414.4(*CCDC88C*):c.322G>A (p.Gly108Ser) | Het | 0.000225 | VUS (PM2) | - |
|  |  |  |  | NM_000406.2(*GNRHR*):c.317A>G (p.Gln106Arg) | Het | 0.002749 | LP (PM1, PP2, PM2, PP5) | PMID: 9371856 PMID: 28611058* |
|  |  |  |  | NM_000406.2(*GNRHR*):c.937_947delTTTTTAAACCC (p.Phe313Metfs*3) | Het | - | LP (PVS1, PM2, PP1) | PMID: 28611058* |
|  |  |  |  | NM_012414.3(*RAB3GAP2*):c.745C>T (p.Pro249Ser) | Het | 0.000004 | VUS (PM2, PP1) | - |
| 7013 | M | KS |  | NM_032242.3(*PLXNA1*):c.2564G>A (p.Arg855His) | Het | 0.000145 | VUS (PM2, PP2, BP4) | - |
| 7030 | M | KS |  | NM_001273.3(*CHD4*):c.86C>G (p.Pro29Arg) | Het | - | VUS (PM2, PP2) | - |
|  |  |  |  | NM_001963.5(*EGF*):c.531G>C (p.Glu177Asp) | Het | - | VUS (PM2) | - |
|  |  |  |  | NM_178822.4(*IGSF10*):c.467G>T (p.Arg156Leu) | Het | 0.000354 | VUS (PM2, PP5) | PMID: 27137492 |
| 7031 | F | nHH |  | NM_000264.3(*PTCH1*):c.3883C>T (p.Pro1295Ser) | Het | - | VUS (PM2, PP2) | - |
| 7039 | M | KS |  | NM_033661.4(*WDR4*):c.652T>C (p.Tyr218His) | Het | 0.000063 | VUS (PM2) | - |
| 7074 | M | KS | Pituitary hypoplasia, low IGF1. One affected brother and two affected nephews | NM_000479.3(*AMH*):c.1054C>T (p.Pro352Ser) | Het | 0.000476 | VUS | PMID: 28505284 |
|  |  |  |  | NM_000216.4(*ANOS1*):c.571C>T (p.Arg191*) | Hemi | - | P (PVS1, PM2, PM3, PP1) | PMID: 11297579 PMID: 28122887* |
|  |  |  |  | NM_032242.3(*PLXNA1*):c.965G>A (p.Arg322His) | Het | 0.000108 | VUS (PM2, PP2, BP4) | - |
| 7075 | M | KS | ACTH deficiency | NM_001126128.1(*PROK2*):c.163delA (p.Ile55*) | Homo | 0.000113 | P (PVS1, PS4, PM2) | PMID: 17959774 |
|  |  |  |  | NM_001145357.2(*SIN3A)*:c.2252A>G (p.Asn751Ser) | Het | 0.000096 | VUS (PM2, PP2) | - |
| 7081 | M | nHH |  | NM_017780.3(*CHD7*):c.3245C>T (p.Thr1082Ile) | Het | - | LP (PM2, PM5, PP2, PP3) | PMID: 30733481* |
|  |  |  |  | NM_023110.2(*FGFR1*):c.12G>T (p.Trp4Cys) | Het | - | VUS (PM2, PP2) | PMID: 26277103* |
|  |  |  |  | NM_201266.1(*NRP2*):c.2552C>T (p.Ser851Leu) | Het | 0.000056 | VUS (PM2, BP4) | - |
| 7095 | M | KS |  | NM_018117.11(*WDR11*):c.811C>T (p.Leu271Phe) | Het | - | VUS (PM2) | - |
| 7183 | M | nHH | Obesity, reversal of hypogonadism | NM_001174116.3(*DMXL2*):c.2069G>A (p.Ser690Asn) | Het | - | VUS (PM2, PP2, BP4) | - |
| 7334 | M | nHH |  | None |  |  |  |  |
| 7335 | M | KS | Reversal of hypogonadism | NM_173648.4(*CCDC141*):c.1394G>C (p.Gly465Ala) | Het | 0.000188 | VUS (PM2, BP4) | - |
|  |  |  |  | NM_173648.4(*CCDC141*):c.1396delT (p.Tyr466Thrfs*33) | Het | 0.000188 | VUS (PM2) | - |
|  |  |  |  | NM_000921.4(*PDE3A*):c.1807G>A (p.Glu603Lys) | Het | 0.000007 | VUS (PM2, BP4) | - |
|  |  |  |  | NM_007055.3(*POLR3A*):c.1177C>T (p.Pro393Ser) | Het | 0.000004 | VUS (PM2, PP2, PP3) | - |
| 7349 | M | KS | Low IGF1 | NM_005045.3(*RELN*):c.7634C>T (p.Ala2545Val) | Het | 0.000216 | VUS (PP2, BP6) | PMID: 29969175 |
| 7355 | F | nHH |  | NM_017780.3(*CHD7*):c.3036G>C (p.Leu1012Phe) | Het | - | VUS (PM2, PP2) | - |
|  |  |  |  | NM_004004.6(*GJB2*):c.284T>C (p.Val95Ala) | Het | 0.000004 | LP (PM1, PM2, PM5, PP2, PP3, PP5) | - |
|  |  |  |  | NM_000406.2(*GNRHR*):c.317A>G (p.Gln106Arg) | Het | 0.002749 | LP (PM1, PP2, PM2, PP5) | PMID: 9371856 PMID: 28611058* |
|  |  |  |  | NM_012414.3(*RAB3GAP2*):c.3058T>A (p.Tyr1020Asn) | Het | 0.000032 | VUS (PM2) | - |
| 7359 | M | nHH | Hearing impairment, chronic renal failure | NM_019066.5(*MAGEL2*):c.2028G>T (p.Glu676Asp) | Het | 0.000016 | VUS (PM2) | - |
|  |  |  |  | NM_000264.3(*PTCH1*):c.3487G>A (p.Gly1163Ser) | Het | 0.000513 | VUS (PM5, PP2, PP3, BP6) | PMID: 25260786 |
| 7363 | M | nHH |  | NM_000406.2(*GNRHR*):c.401T>G (p.Val134Gly) | Het | 0.000028 | P (PS3, PM1, PM2, PM3, PP2, PP3) | PMID: 25016926 PMID: 28611058* |
|  |  |  |  | NM_000406.2(*GNRHR*):c.415C>T (p.Arg139Cys) | Het | 0.000012 | P (PM1, PM2, PM3, PM5, PP2, PP3) | PMID: 17179725 PMID: 28611058* |
|  |  |  |  | NM_178822.4(*IGSF10*):c.4187C>T (p.Ser1396Phe) | Het | 0.000200 | VUS (PM2, BP4) | PMID: 31726455 |
|  |  |  |  | NM_032242.3(*PLXNA1*):c.841A>C (p.Lys281Gln) | Het | - | VUS (PM2, PP2) | - |
| 7367 | M | nHH |  | NM_005215.3(*DCC*):c.527A>G (p.Asn176Ser) | Het | 0.000124 | VUS | PMID: 24808016 |
|  |  |  |  | NM_023110.2(*FGFR1*):c.242T>C (p.Ile81Thr) | Het | - | VUS (PM2, PP2) | PMID: 35457241* |
|  |  |  |  | NM_005996.3(*TBX3*):c.1122G>C (p.Lys374Asn) | Het | - | VUS (PM2) | - |
| 7374 | M | nHH | Hearing impairment | NM_004822.2(*NTN1*):c.1466A>G (p.Lys489Arg) | Het | 0.000145 | VUS (PM2) | PMID: 28945198 |
| 7379 | M | KS | Cryptorchidism, pituitary hypoplasia, low IGF1 | None |  |  |  |  |
| 7381 | M | KS | Hearing impairment, mental retardation | NM_017780.3(*CHD7*):c.1808_1811delACAA (p.Asn603Thrfs*4) | Het | - | P (PVS1, PS4, PM2) | PMID: 19159393 |
| 7386 | M | nHH |  | NM_001030055.2(*ARHGAP5*):c.4430T>G (p.Val1477Gly) | Het | 0.000081 | VUS (PM2, BP4) | - |
|  |  |  |  | NM_014564.4(*LHX3*):c.964G>T (p.Val322Phe) | Het | - | VUS (PM2, BP4) | - |
| 7390 | M | nHH |  | NM_144773.3(*PROKR2*):c.253C>T (p.Arg85Cys) | Het | 0.000601 | LP (PM1, PM2, PM5, PP5) | PMID: 17054399 |
|  |  |  |  | NM_213653.3(*HJV*):c.904G>A (Glu302Lys) | Het | 0.000290 | VUS (PP2) | PMID: 15254010 |
| 7391 | M | KS |  | None |  |  |  |  |
| 7407 | M | nHH |  | NM_000510.2(*FSHB*):c.177C>A (p.Asp59Glu) | Het | - | VUS (PM2) | - |
|  |  |  |  | NM_000406.2(*GNRHR*):c.785G>A (p.Arg262Gln) | Het | 0.001789 | P (PM1, PM2, PM3, PM5, PP2, PP3) | PMID: 9371856 PMID: 28611058* |
|  |  |  |  | NM_000406.2(*GNRHR*):c.937_947delTTTTTAAACCC (p.Phe313Metfs*3) | Het | - | LP (PVS1, PM2) | PMID: 28611058* |
|  |  |  |  | NM_014875.2(*KIF14*):c.1685G>A (p.Gly562Asp) | Het | 0.000004 | VUS (PM2) | - |
|  |  |  |  | NM_002673.4(*PLXNB1*):c.655G>A (p.Val219Met) | Het | 0.001526 | VUS (PM2, BS2, PP2) | - |
| 7506 | M | nHH | Cryptorchidism | NM_015662.2(*IFT172*):c.806T>A (p.Ile269Asn) | Het | - | VUS (PM2, BP4) | - |
|  |  |  |  | NM_015662.2(*IFT172*):c.3196C>T (p.Arg1066Trp) | Het | 0.000024 | VUS (PM2) | - |
|  |  |  |  | NM_005045.3(*RELN*):c.4279T>C (p.Cys1427Arg) | Het | - | VUS (PM2, PP2, PP3) | - |
|  |  |  |  | NM_005045.3(*RELN*):c.7634C>T (p.Ala2545Val) | Het | 0.000216 | VUS (PP2, BP6) | PMID: 29969175 |
| 7612 | M | nHH |  | NM_022370.3(*ROBO3*):c.1655C>G (p.Pro552Arg) | Het | 0.000036 | VUS (PM2) | - |
| 7625 | M | nHH | Pituitary hypoplasia, ACTH deficiency | NM_000620.4(*NOS1*):c.335C>T (p.Thr112Ile) | Het | 0.000161 | VUS (PM2, PP2, BP4) | - |
|  |  |  |  | NM_005045.3(*RELN*):c.6749A>G (p.Tyr2250Cys) | Het | - | VUS (PM2, PP2) | - |
|  |  |  |  | NM_003193.3(*TBCE*):c.830C>T (p.Pro277Leu) | Het | 0.000002 | VUS (PM2) | - |
| 7628 | M | nHH |  | NM_032458.3(*PHF6*):c.1045A>G (p.Lys349Glu) | Hemi | 0.000011 | VUS (PM2, BS2, PP2) | - |
| 8214 | M | KS | Cryptorchidism, pituitary hypoplasia | NM_001030055.2(*ARHGAP5*):c.2067dupA (p.Tyr690Ilefs*4) | Het | - | LP (PVS1, PM2) | - |
|  |  |  |  | NM_025000.3(*DCAF17*):c.552_554delACA (p.Gln184del) | Het | 0.000016 | VUS (PM2, PM4) | - |
|  |  |  |  | NM_005103.5(*FEZ1*):c.960C>G (p.Ile320Met) | Het | 0.000085 | VUS (PM2, BP4) | - |
|  |  |  |  | NM_000230.2(*LEP*):c.397G>A (p.Gly133Ser) | Het | - | VUS (PM2, BP4) | - |
|  |  |  |  | NM_201266.1(*NRP2*):c.1000C>T (p.Arg334Cys) | Het | 0.001622 | VUS (PM2, BS2, PP3) | PMID: 33212964 |
| 8255 | M | nHH | Cryptorchidism, pituitary hypoplasia | NM_004439.5(*EPHA*5):c.1276G>A (p.Val426Ile) | Het | 0.000804 | VUS (PM2) | - |
|  |  |  |  | NM_000825.3(*GNRH1*):c.99delA (p.Leu34Cysfs*12) | Homo | - | LP (PVS1, PM2) | PMID: 19567835 |
|  |  |  |  | NM_015100.4(*POGZ*):c.364A>G (p.Thr122Ala) | Het | - | VUS (PM2, PP2, BP4) | - |
| 8276 | F | KS | Pituitary hypoplasia, abnormal neck rotation | NM_001126128.1(*PROK2*):c.297dupT (p.Gly100Trpfs*22) | Homo | 0.000110 | LP (PVS1, PM2) | PMID: 17054399 |
|  |  |  |  | NM_003108.4(*SOX11*):c.650T>C (p.Val217Ala) | Het | 0.000005 | VUS (PM2, PP2) | - |
| 8297 | M | KS |  | NM_001963.5(*EGF*):c.1478T>C (p.Ile493Thr) | Het | - | VUS (PM2, PP3) | PMID: 19319977 |
| 8299 | F | nHH |  | NM_001873.3(*CPE*):c.884C>G (p.Pro295Arg) | Het | - | VUS (PM2, BP4) | - |
|  |  |  |  | NM_000406.2(*GNRHR*):c.317A>G (p.Gln106Arg) | Het | 0.002749 | LP (PM1, PP2, PM2, PP5) | PMID: 9371856 PMID: 28611058* |
|  |  |  |  | NM_000406.2(*GNRHR*):c.785G>A (p.Arg262Gln) | Het | 0.001789 | P (PM1, PM2, PM3, PM5, PP2, PP3) | PMID: 9371856 PMID: 28611058* |
|  |  |  |  | NM_032844.5(*MASTL*):c.1774A>G (p.Arg592Gly) | Het | 0.000008 | VUS (PM2, BP4) | - |
|  |  |  |  | NM_012233.3(*RAB3GAP1*):c.244G>A (p.Glu82Lys) | Het | 0.000004 | VUS (PM2, BP4) | - |
|  |  |  |  | NM_018077.2(*RBM28*):c.1744_1745insT (p.Arg582Leufs*5) | Het | - | LP (PVS1, PM2) | - |
|  |  |  |  | NM_033661.4(*WDR4*):c.265C>T (p.Arg89Cys) | Het | 0.000014 | VUS (PM2) | - |
| 8302 | M | nHH |  | NM_000216.4(*ANOS1*):c.31A>G (p.Thr11Ala) | Hemi | - | VUS (PM2, BP4) | - |
|  |  |  |  | NM_021913.3(*AXL*):c.1829G>T (p.Arg610Leu) | Het | 0.000020 | VUS (PM2) | - |
|  |  |  |  | NM_004004.6(*GJB2*):c.101T>C (p.Met34Thr) | Het | 0.008996 | LP ( PM1, PM2, PM5, PP1, PP2, PP3, PP5) | PMID: 9139825 |
|  |  |  |  | NM_000406.2(*GNRHR*):c.317A>G (p.Gln106Arg) | Het | 0.002749 | LP (PM1, PP2, PM2, PP5) | PMID: 9371856 PMID: 28611058* |
|  |  |  |  | NM_032242.3(*PLXNA1*):c.1628G>A (p.Arg543Gln) | Het | 0.000007 | VUS (PM2, PP2) | - |
| 8325 | M | KS | Pituitary hypoplasia | NM_023110.2(*FGFR1*):c.677_678delGCinsAA (p.Gly226Glu) | Het | - | LP (PM1, PM2, PM5, PP2) | - |
|  |  |  |  | NM_017617.4(*NOTCH1*):c.6119A>G (p.Asn2040Ser) | Het | 0.000004 | VUS (PM2, PP2) | PMID: 29177441 |
| 8347 | F | nHH |  | NM_001035235.2(*SRA1*):c.236C>T (p.Pro79Leu) | Het | - | VUS (PM2) | - |
|  |  |  |  | NM_007252.4(*POU6F2*):c.1340C>G (p.Ala447Gly) | Het | 0.000054 | VUS (PM2) | - |
| 8474 | M | KS |  | NM_178822.4(*IGSF10*): c.2610G>A (p.Met870Ile) | Het | 0.000021 | VUS (PM2, BP4) | - |
|  |  |  |  | NM_178822.4(*IGSF10)*:c.5983G>A (p.Val1995Ile) | Het | 0.000772 | VUS (PM2, BS2) | PMID:31726455 |
|  |  |  |  | NM_018344.5(*SLC29A3*): c.59C>G (p.Thr20Arg) | Het | 0.000016 | VUS (PM2, BP4) | - |
|  |  |  |  | NM_006941.3(*SOX10*): c.778G>A (p.Gly260Arg) | Het | 0.000022 | VUS (PM2, PP3) | - |
| 8500 | M | nHH |  | NM_000406.2(*GNRHR*):c.410T>C (p.Leu137Pro) | Homo | - | LP (PM1, PM2, PM3, PP2) | - |

id, identification; M, male; F, female; KS, Kallmann syndrome; nHH, normosmic hypogonadotropic hypogonadism; ACTH, adrenocorticotropic hormone; IGF1, insulin like growth factor 1; Het, heterozygous; Homo, homozygous; Hemi, hemizygous; GnomAD, Genome Aggregation Database; ACMG, American College of Medical Genetics and Genomics. Variants were classified as Pathogenic (P), Likely Pathogenic (LP), Variants of Uncertain Significance (VUS), Likely Benign (LB), or Benign (B), based on the evidence for pathogenicity [very strong (PVS1), strong (PS1-4), moderate (PM1–6), or supporting (PP1–5)] or benign impact [stand-alone (BA), strong (BS1-4), or supporting (BP1-7)]. PMID, PubMed identifier. * Publication by the authors that included the same patient.
