# Supplementary material for: Genetic architecture of congenital hypogonadotropic hypogonadism: insights from analysis of a Portuguese cohort
Source: Hum Reprod Open. 2024 Sep 11;2024(3):hoae053. doi: 10.1093/hropen/hoae053 (PMC11415827; doi:10.1093/hropen/hoae053)
Supplement: hoae053_Supplementary_Data [file hoae053_supplementary_data.zip › Supplementary Table S4.docx]

**Supplementary Table S4.** Rare sequence variants identified in controls.

| **Gene** | **Reference sequence** | **Variant** | **Effect** | **Allele frequency in GnomAD** | **Allele frequency in Portuguese controls** | **ACMG classification** | **Control id** |
| --- | --- | --- | --- | --- | --- | --- | --- |
| *AMH* | NM_000479.3 | c.16C>G (p.Leu6Val) | Missense | 0.000005 | 0.002 | VUS (PM2, BP4) | 5497 |
|  | NM_000479.3 | c.35T>G (p.Val12Gly) | Missense | 0.001422 | 0.004 | LB (BS1, BS2) | 7995, 8177 |
|  | NM_000479.3 | c.53C>T(p.Ala18Val) | Missense | 0.001032 | 0.002 | LB (BS1, BP6) | 7763 |
|  | NM_000479.3 | c.428C>T (p.Thr143Ile) | Missense | 0.005473 | 0.006 | B (BA1, BS2, BP4, BP6) | 5156, 6040, 7835 |
|  | NM_000479.3 | c.553C>G (p.Gln185Glu) | Missense | 0.000353 | 0.002 | VUS (PM2, BP6) | 8092 |
|  | NM_000479.3 | c.556-2A>G | Splice-site | - | 0.002 | LP (PVS1, PM2) | 8232 |
|  | NM_000479.3 | c.790G>C (p.Gly264Arg) | Missense | 0.000129 | 0.004 | VUS (PP3, BS1) | 7634, 7637 |
|  | NM_000479.3 | c.802A>G (p.Thr268Ala) | Missense | 0.000231 | 0.002 | VUS (PM2) | 8045 |
|  | NM_000479.3 | c.864C>G (p.Asp288Glu) | Missense | 0.001269 | 0.002 | LB (PP3, BS1, BS2, BP6) | 6045 |
|  | NM_000479.3 | c.974A>G (p.Gln325Arg) | Missense | 0.005543 | 0.002 | B (BA1, BS2, BP6) | 7635 |
|  | NM_000479.3 | c.1054C>T (p.Pro352Ser) | Missense | 0.000476 | 0.004 | VUS | 5160, 7892 |
|  | NM_000479.3 | c.1193C>T (p.Pro398Leu) | Missense | 0.000650 | 0.004 | LB (BS1, BS2) | 7879, 7885 |
|  | NM_000479.3 | c.1232G>A (p.Cys411Tyr) | Missense | - | 0.004 | VUS (PM2) | 7644, 8022 |
|  | NM_000479.3 | c.1303C>T (p.Arg435Cys) | Missense | - | 0.002 | VUS (PM2) | 7920 |
|  | NM_000479.3 | c.1505G>T (p.Arg502Leu) | Missense | 0.000034 | 0.004 | VUS (PM2) | 5725, 7722 |
|  | NM_000479.3 | c.1556C>T (p.Ala519Val) | Missense | 0.001632 | 0.008 | LB (BS1, BS2, BP4, BP6) | 7736, 7833, 7935, 7958 |
|  | NM_000479.3 | c.1668G>C (p.Glu556Asp) | Missense | - | 0.002 | VUS (PM2) | 7635 |
| *ANOS1* | NM_000216.4 | c.383T>C (p.Leu128Ser) | Missense | 0.000024 | 0.002 | VUS (PM2, BP4) | 7851 |
|  | NM_000216.4 | c.727A>G (p.Thr243Ala) | Missense | 0.000005 | 0.002 | VUS (PM2) | 8018 |
|  | NM_000216.4 | c.1283C>T (p.Pro428Leu) | Missense | 0.000785 | 0.002 | B (BA1, BS2, BP4, BP6) | 7680 |
|  | NM_000216.4 | c.1759G>T (p.Val587Leu) | Missense | 0.002226 | 0.002 | B (BS1, BS2, BP6) | 7853 |
| *ARHGAP35* | NM_004491.5 | c.1090A>G (p.Ile364Val) | Missense | 0.000111 | 0.002 | VUS (PM2, PP2, BP4) | 8304 |
|  | NM_004491.5 | c.1190T>A (p.Met397Lys) | Missense | - | 0.002 | VUS (PM2, PP2, BP4) | 6077 |
|  | NM_004491.5 | c.2230C>T (p.Arg744Cys) | Missense | 0.000036 | 0.002 | VUS (PM2, PP2) | 7943 |
|  | NM_004491.5 | c.2885G>C (p.Ser962Thr) | Missense | 0.000759 | 0.002 | VUS (PM2, PP2, BP4) | 7863 |
|  | NM_004491.5 | c.3362A>G (p.Asn1121Ser) | Missense | 0.000004 | 0.002 | VUS (PM2, PP2) | 8174 |
| *ARHGAP5* | NM_001030055.2 | c.3290C>A (p.Thr1097Lys) | Missense | 0.000096 | 0.002 | VUS (PM2, PP2) | 7674 |
|  | NM_001030055.2 | c.3340G>A (p.Asp1114Asn) | Missense | - | 0.002 | VUS (PM2, PP2) | 8296 |
|  | NM_001030055.2 | c.3508A>G (p.Arg1170Gly) | Missense | 0.000181 | 0.002 | VUS (PM2, PP2) | 7935 |
|  | NM_001030055.2 | c.4216G>A (p.Ala1406Thr) | Missense | 0.005966 | 0.004 | B (PP2, BS1, BS2) | 5555, 8215 |
| *ARL6* | NM_177976.1 | c.30G>C (p.Leu10Phe) | Missense | - | 0.002 | VUS (PM2, PP2, BP4) | 8298 |
|  | NM_177976.1 | c.538C>T (p.Gln180*) | Nonsense | 0.000008 | 0.002 | VUS (PM2, PVS1) | 7792 |
| *AXL* | NM_021913.3 | c.53C>T (p.Ala18Val) | Missense | 0.000314 | 0.002 | LB (PM2, BP4, BP6) | 8024 |
|  | NM_021913.3 | c.1549G>A (p.Gly517Ser) | Missense | 0.004279 | 0.002 | LB (PM2, BS2, BP6) | 7853 |
|  | NM_021913.3 | c.2399G>A (p.Arg800Gln) | Missense | 0.000085 | 0.002 | VUS (PM2) | 7839 |
| *B9D1* | NM_015681.3 | c.278A>G (p.Asp93Gly) | Missense | - | 0.002 | VUS (PM2, PP3) | 6077 |
|  | NM_015681.3 | c.376T>A (p.Ser126Thr) | Missense | 0.000272 | 0.002 | VUS (PM2, BP6) | 8026 |
|  | NM_015681.3 | c.380C>T (p.Thr127Met) | Missense | 0.000028 | 0.002 | VUS (PM2) | 7678 |
| *BBIP1* | NM_001195306.2 | c.135A>G (p.Ile45Met) | Missense | 0.000012 | 0.002 | VUS (PM2, BP4) | 8221 |
| *BBS1* | NM_024649.5 | c.158dupA (p.Leu54Alafs*45) | Frameshift | 0.000004 | 0.002 | P (PVS1, PM2, PP5) | 8025 |
|  | NM_024649.5 | c.616T>G (p.Leu206Val) | Missense | 0.000516 | 0.002 | LB (PM2, BS2, BP6) | 8206 |
|  | NM_024649.5 | c.725T>C (p.Met242Thr) | Missense | 0.000036 | 0.002 | VUS (PM2) | 8095 |
|  | NM_024649.5 | c.1036G>A (p.Val346Ile) | Missense | 0.000841 | 0.002 | B (BS1, BS2, BP6) | 8178 |
|  | NM_024649.5 | c.1318C>T (p.Arg440*) | Nonsense | 0.000008 | 0.002 | P (PVS1, PM2, PM3) | 7945 |
| *BBS10* | NM_024685.4 | c.137T>G (p.Leu46Arg) | Missense | - | 0.002 | VUS (PM2, PP2, PP3) | 8181 |
|  | NM_024685.4 | c.424G>A (p.Asp142Asn) | Missense | 0.007475 | 0.008 | B (PP2, BS1, BS2, BP6) | 5153, 7694, 7894, 8181 |
|  | NM_024685.4 | c.765G>A (p.Met255Ile) | Missense | 0.000595 | 0.002 | VUS (PM1, PP2, PM2, BP6) | 7116 |
|  | NM_024685.4 | c.1634C>T (p.Ser545Phe) | Missense | 0.000004 | 0.002 | VUS (PM2, PP2) | 8294 |
| *BBS5* | NM_152384.2 | c.551A>G (p.Asn184Ser) | Missense | 0.004164 | 0.002 | B (PP3, BA1, BS2, BP6) | 8294 |
|  | NM_152384.2 | c.620G>A (p.Arg207His) | Missense | 0.006398 | 0.004 | B (PP3, BA1, BS2, BP6) | 7956, 7993 |
|  | NM_152384.2 | c.751A>G (p.Asn251Asp) | Missense | 0.001036 | 0.002 | VUS (BS1) | 7883 |
| *CASR* | NM_000388.3 | c.2265G>T (p.Glu755Asp) | Missense | 0.000025 | 0.002 | VUS (PM1, PM2, PP2, BP6) | 7870 |
|  | NM_000388.3 | c.2563A>C (p.Asn855His) | Missense | - | 0.004 | VUS (PM2, PP2) | 7687, 7840 |
| *CCDC141* | NM_173648.4 | c.103-1G>T | Splice-site | 0.000018 | 0.002 | VUS (PM2) | 7684 |
|  | NM_173648.4 | c.665G>A (p.Arg222His) | Missense | 0.001087 | 0.002 | VUS (PM2, BP4) | 8180 |
|  | NM_173648.4 | c.781G>C (p.Val261Leu) | Missense | 0.002886 | 0.006 | B (PP3, BS1, BS2, BP6) | 7637, 7694, 8045 |
|  | NM_173648.4 | c.1394G>C (p.Gly465Ala) | Missense | 0.000188 | 0.002 | VUS (PM2, BP4) | 7769 |
|  | NM_173648.4 | c.1396delT (p.Tyr466Thrfs*33) | Frameshift | 0.000188 | 0.002 | VUS (PM2) | 7769 |
|  | NM_173648.4 | c.1521A>C (p.Gln507His) | Missense | 0.003767 | 0.006 | LB (PM2, BS2, BP4, BP6) | 6077, 7641, 7883 |
|  | NM_173648.4 | c.1600G>A (p.Val534Ile) | Missense | 0.000545 | 0.002 | VUS (PM2, BP4) | 8195 |
|  | NM_173648.4 | c.2299G>A (p.Asp767Asn) | Missense | 0.003676 | 0.002 | LB (PM2, BS2, BP4, BP6) | 6051 |
|  | NM_173648.4 | c.2614C>G (p.Leu872Val) | Missense | 0.000679 | 0.002 | LB (PM2, BP4, BP6) | 8195 |
|  | NM_173648.4 | c.3091A>G (p.Thr1031Ala) | Missense | 0.000016 | 0.002 | VUS (PM2) | 8184 |
|  | NM_173648.4 | c.3321delA (p.Leu1107Phefs*12) | Frameshift | 0.000292 | 0.004 | VUS (PM2) | 7763, 7943 |
|  | NM_173648.4 | c.3782C>T (p.Ala1261Val) | Missense | 0.000108 | 0.002 | LB (PM2, BS2, BP4) | 7656 |
|  | NM_173648.4 | c.4369G>A (p.Val1457Ile) | Missense | 0.004793 | 0.004 | B (BS2, BP6) | 7663, 7836 |
|  | NM_173648.4 | c.4565C>T (p.Ser1522Phe) | Missense | 0.000029 | 0.002 | VUS (PM2, BP4) | 7770 |
| *CCDC88C* | NM_001080414.4 | c.41A>G (p.Gln14Arg) | Missense | - | 0.002 | VUS (PM2, BP4) | 7920 |
|  | NM_001080414.4 | c.1669A>G (p.Lys557Glu) | Missense | - | 0.002 | VUS (PM2, BP4) | 8044 |
|  | NM_001080414.4 | c.1878G>C (p.Lys626Asn) | Missense | 0.001281 | 0.002 | LB (BS1, BP4, BP6) | 7712 |
|  | NM_001080414.4 | c.1885C>T (p.Arg629Trp) | Missense | 0.000075 | 0.002 | VUS (PM2) | 7676 |
|  | NM_001080414.4 | c.2819T>C (p.Leu940Pro) | Missense | 0.000127 | 0.002 | VUS (PM2) | 8232 |
|  | NM_001080414.4 | c.2958G>A (p.Met986Ile) | Missense | 0.000182 | 0.002 | VUS (PM2, BP4) | 5147 |
|  | NM_001080414.4 | c.3202G>T (p.Ala1068Ser) | Missense | 0.001601 | 0.004 | B (BS1, BS2, BP4, BP6) | 6077, 8128 |
|  | NM_001080414.4 | c.3419A>G (p.Gln1140Arg) | Missense | 0.001630 | 0.002 | B (BA1, BS2, BP6) | 7633 |
|  | NM_001080414.4 | c.3596A>G (p.His1199Arg) | Missense | 0.000095 | 0.002 | VUS (PM2) | 6908 |
|  | NM_001080414.4 | c.3628G>A (p.Gly1210Arg) | Missense | 0.000049 | 0.002 | VUS (PM2, BP4) | 7778 |
|  | NM_001080414.4 | c.3640G>A (p.Gly1214Ser) | Missense | 0.000965 | 0.002 | LB (BS1, BP4, BP6) | 7878 |
|  | NM_001080414.4 | c.4265C>T (p.Ser1422Leu) | Missense | 0.003619 | 0.004 | B (BA1, BS2, BP4, BP6) | 7813, 7924 |
|  | NM_001080414.4 | c.4327G>A (p.Ala1443Thr) | Missense | 0.001922 | 0.008 | B (BA1, BS1, BS2, BP4, BP6) | 5546, 7648, 7792, 7839 |
|  | NM_001080414.4 | c.4433T>C (p.Val1478Ala) | Missense | 0.001841 | 0.002 | B (BA1, BS2, BP4, BP6) | 7728 |
|  | NM_001080414.4 | c.5087T>C (p.Leu1696Pro) | Missense | 0.002496 | 0.002 | B (BS1, BS2, BP6) | 7804 |
|  | NM_001080414.4 | c.5090G>A (p.Ser1697Asn) | Missense | 0.000099 | 0.002 | LB (PM2, BP4, BP6) | 7985 |
|  | NM_001080414.4 | c.5183T>C (p.Phe1728Ser) | Missense | 0.002143 | 0.002 | B (BA1, BS2, BP4, BP6) | 7728 |
|  | NM_001080414.4 | c.5332T>C (p.Ser1778Pro) | Missense | 0.000012 | 0.002 | VUS (PM2, BP4) | 7820 |
|  | NM_001080414.4 | c.5899G>A (p.Gly1967Arg) | Missense | 0.000015 | 0.002 | VUS (PM2, BP4) | 8253 |
|  | NM_001080414.4 | c.5927G>C (p.Gly1976Ala) | Missense | 0.000767 | 0.004 | LB (BS1, BP4) | 5559, 7686 |
|  | NM_001080414.4 | c.5980C>G (p.Arg1994Gly) | Missense | 0.006488 | 0.004 | B (BA1, BS2, BP4, BP6) | 7883, 8118 |
|  | NM_001080414.4 | c.6053C>T (p.Pro2018Leu) | Missense | 0.000045 | 0.002 | VUS (PM2, BP4) | 7853 |
| *CCKAR* | NM_000730.3 | c.97C>A (p.Pro33Thr) | Missense | 0.002434 | 0.002 | B (BS1, BS2) | 8296 |
|  | NM_000730.3 | c.329G>A (p.Gly110Glu) | Missense | 0.000011 | 0.002 | VUS (PM2, PP3) | 7791 |
|  | NM_000730.3 | c.590G>A (p.Arg197His) | Missense | 0.000014 | 0.002 | VUS (PM2) | 7986 |
|  | NM_000730.3 | c.628C>T (p.His210Tyr) | Missense | 0.002487 | 0.004 | LB (PM2, BS2, BP4) | 5147, 7884 |
|  | NM_000730.3 | c.685T>C (p.Tyr229His) | Missense | 0.000025 | 0.002 | VUS (PM2, PP3) | 7744 |
|  | NM_000730.3 | c.799G>A (p.Asp267Asn) | Missense | 0.000032 | 0.002 | VUS (PM2) | 8072 |
|  | NM_000730.3 | c.1006C>T (p.Arg336Trp) | Missense | 0.000024 | 0.002 | VUS (PM2) | 8232 |
| *CDKN1C* | NM_000076.2 | c.355C>T (p.Leu119Phe) | Missense | 0.000011 | 0.002 | VUS (PM2) | 7715 |
| *CDON* | NM_016952.4 | c.458G>A (p.Arg153His) | Missense | 0.000008 | 0.002 | VUS (PM2) | 7970 |
|  | NM_016952.4 | c.791C>A (p.Pro264Gln) | Missense | 0.000103 | 0.002 | VUS (PM2, BP6) | 8057 |
|  | NM_016952.4 | c.848C>T (p.Ala283Val) | Missense | 0.000014 | 0.002 | VUS (PM2, BP4) | 7949 |
|  | NM_016952.4 | c.1495A>G (p.Ile499Val) | Missense | 0.000004 | 0.002 | VUS (PM2, BP4) | 7745 |
|  | NM_016952.4 | c.2051C>G (p.Thr684Ser) | Missense | 0.007158 | 0.004 | B (BS1, BS2, BP4, BP6) | 6077, 7642 |
|  | NM_016952.4 | c.2177G>T (p.Arg726Leu) | Missense | - | 0.002 | VUS (PM2) | 7111 |
|  | NM_016952.4 | c.2383G>A (p.Val795Ile) | Missense | 0.000074 | 0.002 | VUS (PM2, BP6) | 7656 |
|  | NM_016952.4 | c.2462G>A (p.Arg821His) | Missense | 0.000605 | 0.004 | LB (PM2, BS2, BP6) | 5473, 5547 |
|  | NM_016952.4 | c.3367A>G (p.Lys1123Glu) | Missense | 0.000008 | 0.002 | VUS (PM2) | 8235 |
|  | NM_016952.4 | c.3689T>A (p.Ile1230Asn) | Missense | 0.000011 | 0.002 | VUS (PM2, BS2) | 7889 |
| *CHD4* | NM_001273.3 | c.304C>T (p.Arg102Cys) | Missense | 0.000039 | 0.002 | VUS (PM2, PP2, PP3) | 7689 |
|  | NM_001273.3 | c.1064G>A (p.Gly355Asp) | Missense | 0.000315 | 0.002 | VUS (PM2, PP2, PP3, BP6) | 7879 |
|  | NM_001273.3 | c.3088A>G (p.Met1030Val) | Missense | 0.000099 | 0.002 | VUS (PP2) | 7952 |
| *CHD7* | NM_017780.3 | c.1018A>G (p.Met340Val) | Missense | 0.004606 | 0.002 | B (PP2, BA1, BS2, BP6) | 8030 |
|  | NM_017780.3 | c.1046A>G (p.Asn349Ser) | Missense | 0.000089 | 0.002 | LB (PM2, PP2, BP4, BP6) | 5469 |
|  | NM_017780.3 | c.1056_1070delATTCCCATCAAACAG (p.Phe353_Ser357del) | Inframe deletion | - | 0.002 | VUS (PM2, PM4) | 7929 |
|  | NM_017780.3 | c.1472T>A (p.Ile491Asn) | Missense | - | 0.002 | LB (PM2, PP2, BP4, BP6) | 7894 |
|  | NM_017780.3 | c.1554G>T (p.Gln518His) | Missense | 0.000016 | 0.002 | VUS (PM2, PP2, BP6) | 8018 |
|  | NM_017780.3 | c.2053_2058dupGCAAAA (p.Ala685_Lys686dup) | Inframe insertion | 0.006230 | 0.002 | B (PM4, BA1, BS2, BP6) | 5155 |
|  | NM_017780.3 | c.2185A>G (p.Lys729Glu) | Missense | 0.000357 | 0.002 | LB (PP2, BS2, BP6) | 8181 |
|  | NM_017780.3 | c.2209_2211delCCT (p.Pro737del) | Inframe deletion | 0.000086 | 0.002 | VUS (PM4, BP6) | 7637 |
|  | NM_017780.3 | c.2787C>G (p.Ile929Met) | Missense | 0.000018 | 0.002 | VUS (PM2, PP2, BP6) | 8265 |
|  | NM_017780.3 | c.2830C>T (p.Arg944Cys) | Missense | 0.000028 | 0.002 | LP (PM2, PM5, PP2, PP3) | 8298 |
|  | NM_017780.3 | c.4972G>C (p.Glu1658Gln) | Missense | - | 0.002 | VUS (PM2, PP2) | 6052 |
|  | NM_017780.3 | c.5533G>A (p.Gly1845Arg) | Missense | 0.000040 | 0.002 | VUS (PM2, PP2, PP3) | 6052 |
|  | NM_017780.3 | c.7253G>A (p.Arg2418Gln) | Missense | 0.000068 | 0.002 | VUS (PM2, PM5, PP2, BP6) | 8054 |
|  | NM_017780.3 | c.7579A>C (p.Met2527Leu) | Missense | 0.002038 | 0.002 | LB (PP2, BS1, BS2, BP6) | 8126 |
|  | NM_017780.3 | c.8950C>T (p.Leu2984Phe) | Missense | 0.003887 | 0.004 | B (PP2, BA1, BS2, BP4, BP6) | 8212, 8235 |
| *CHL1* | NM_006614.2 | c.704C>T (p.Ser235Leu) | Missense | 0.000950 | 0.002 | B (PM2, BS2, BP4, BP6) | 8294 |
|  | NM_006614.2 | c.946G>A (p.Val316Ile) | Missense | 0.001210 | 0.002 | B (BS2, BP4, BP6) | 7850 |
|  | NM_006614.2 | c.1706G>A (p.Ser569Asn) | Missense | 0.000354 | 0.002 | LB (PM2, BP4, BP6) | 7851 |
|  | NM_006614.2 | c.2488G>A (p.Val830Met) | Missense | 0.000024 | 0.002 | VUS (PM2, BP4) | 7111 |
|  | NM_006614.2 | c.3153G>C (p.Glu1051Asp) | Missense | 0.000882 | 0.008 | LB (PM2, BP4, BP6) | 5575, 7111, 7744, 8167 |
|  | NM_006614.2 | c.3437A>C (p.Asp1146Ala) | Missense | - | 0.002 | VUS (PM2, PP3) | 8095 |
| *CNTN2* | NM_005076.3 | c.191G>A (p.Arg64Gln) | Missense | 0.000036 | 0.002 | VUS (PM2, PP2) | 6045 |
|  | NM_005076.3 | c.689C>T (p.Ala230Val) | Missense | 0.000040 | 0.002 | VUS (PM2, PP2, BP4) | 5555 |
|  | NM_005076.3 | c.821G>A (p.Arg274His) | Missense | 0.000121 | 0.002 | VUS (PM2, PP2) | 8079 |
|  | NM_005076.3 | c.947C>T (p.Thr316Ile) | Missense | 0.005082 | 0.008 | B (PP2, BA1, BS2, BP4, BP6) | 5153, 7722, 7924, 8054 |
|  | NM_005076.3 | c.1276C>T (p.Arg426Cys) | Missense | 0.000020 | 0.002 | VUS (PM2, PP2) | 8072 |
|  | NM_005076.3 | c.1975A>G (p.Asn659Asp) | Missense | 0.008071 | 0.004 | B (PP2, BA1, BS2, BP4, BP6) | 8051, 8221 |
|  | NM_005076.3 | c.2050C>T (p.Arg684Trp) | Missense | 0.000004 | 0.002 | VUS (PM2, PP2) | 5546 |
|  | NM_005076.3 | c.2307G>C (p.Gln769His) | Missense | - | 0.002 | VUS (PM2, PP2, BP4) | 8022 |
|  | NM_005076.3 | c.3011G>A (p.Gly1004Glu) | Missense | - | 0.002 | VUS (PM2, PP2, PP3) | 8126 |
|  | NM_005076.3 | c.3014-2A>G | Splice-site | - | 0.002 | VUS (PVS1, PM2) | 7873 |
| *CPE* | NM_001873.3 | c.1268C>T (p.Ala423Val) | Missense | 0.004678 | 0.004 | B (BS1, BS2, BP6) | 7703, 7993 |
|  | NM_001873.3 | c.1309G>A (p.Val437Ile) | Missense | 0.000539 | 0.002 | VUS (PM2, BP4) | 8253 |
| *CRY1* | NM_004075.5 | c.199C>T (p.Arg67*) | Nonsense | 0.000020 | 0.002 | VUS (PM2) | 8203 |
|  | NM_004075.5 | c.1718C>T (p.Thr573Ile) | Missense | 0.000004 | 0.002 | VUS (PM2, BP4) | 7870 |
| *CXCR4* | NM_003467.2 | c.50G>A (p.Gly17Asp) | Missense | 0.000008 | 0.002 | VUS (PM2, BP4) | 7745 |
| *CYP19A1* | NM_031226.2 | c.578G>A (p.Arg193His) | Missense | 0.000048 | 0.002 | VUS (PM2, BP4) | 8021 |
|  | NM_031226.2 | c.1094G>A (p.Arg365Gln ) | Missense | 0.000032 | 0.002 | P (PS4, PP3, PM2) | 5463 |
| *DCC* | NM_005215.3 | c.527A>G (p.Asn176Ser) | Missense | 0.000124 | 0.002 | VUS | 8018 |
|  | NM_005215.3 | c.744T>G (p.Asn248Lys) | Missense | 0.000028 | 0.002 | VUS (PM2) | 7945 |
|  | NM_005215.3 | c.1157G>A (p.Arg386Gln) | Missense | 0.000251 | 0.002 | LB (PM2, BS2, BP4, BP6) | 7804 |
|  | NM_005215.3 | c.1256A>G (p.Lys419Arg) | Missense | 0.002868 | 0.004 | LB (BS1, BP4, BP6) | 7667, 8184 |
|  | NM_005215.3 | c.1409G>A (p.Gly470Asp) | Missense | 0.002885 | 0.002 | B (BA1, BS2, BP6) | 7686 |
|  | NM_005215.3 | c.1904A>G (p.Asn635Ser) | Missense | 0.000134 | 0.002 | VUS (PM2, BP4) | 7884 |
|  | NM_005215.3 | c.3404C>T (p.Thr1135Ile) | Missense | 0.000012 | 0.002 | VUS (PM2, BP4) | 7813 |
|  | NM_005215.3 | c.4027C>T (p.Arg1343Cys) | Missense | 0.000141 | 0.002 | VUS (PM2) | 7675 |
| *DLG2* | NM_001142699.1 | c.1001A>G (p.Asp334Gly) | Missense | 0.000071 | 0.002 | VUS (PM2) | 8182 |
|  | NM_001142699.1 | c.1337C>T (p.Thr446Ile) | Missense | 0.000742 | 0.004 | LB (PM2, BS2, BP4) | 5147, 7675 |
|  | NM_001142699.1 | c.1541T>C (p.Leu514Pro) | Missense | 0.000946 | 0.004 | LB (PM2, BS2, BP6) | 5158, 8044 |
| *DLX5* | NM_005221.5 | c.702C>A (p.Ser234Arg) | Missense | 0.009918 | 0.006 | B (BS1, BS2, BP6) | 7641, 7728, 7844 |
|  | NM_005221.5 | c.712C>A (p.His238Asn) | Missense | 0.000195 | 0.002 | VUS (PM2) | 8189 |
| *DMXL2* | NM_001174116.3 | c.23C>G (p.Thr8Ser) | Missense | 0.004346 | 0.002 | B (PP2, BA1, BS1, BS2, BP4, BP6) | 8041 |
|  | NM_001174116.3 | c.368A>G (p.Asn123Ser) | Missense | 0.001332 | 0.006 | B (PP2, BA1, BS2, BP4, BP6) | 7828,7837, 8105 |
|  | NM_001174116.3 | c.1235G>A (p.Arg412Gln) | Missense | 0.000024 | 0.002 | VUS (PM2, PP2, BP4) | 7970 |
|  | NM_001174116.3 | c.1318C>T (p.Arg440Trp) | Missense | 0.000043 | 0.002 | VUS (PM2, PP2) | 7952 |
|  | NM_001174116.3 | c.1427C>G (p.Thr476Ser) | Missense | 0.000124 | 0.002 | VUS (PM2, PP2, BP4) | 8215 |
|  | NM_001174116.3 | c.1463C>T (p.Thr488Met) | Missense | 0.000382 | 0.002 | LB (PP2, BS1, BS2) | 5546 |
|  | NM_001174116.3 | c.2096T>C (p.Ile699Thr) | Missense | 0.000509 | 0.002 | LB (PP2, BS1, BP4, BP6) | 8041 |
|  | NM_001174116.3 | c.2806G>A (p.Val936Ile) | Missense | 0.000099 | 0.002 | LB (PP2, BS1, BP4) | 8045 |
|  | NM_001174116.3 | c.2866C>A (p.Pro956Thr) | Missense | 0.002185 | 0.002 | B (PP2, BA1, BS2, BP4, BP6) | 8045 |
|  | NM_001174116.3 | c.3935T>C (p.Val1312Ala) | Missense | 0.001168 | 0.002 | B (PP2, BA1, BS2, BP4, BP6) | 7850 |
|  | NM_001174116.3 | c.4187C>T (p.Ser1396Phe) | Missense | 0.001582 | 0.002 | B (PP2, BS1, BS2, BP6) | 8022 |
|  | NM_001174116.3 | c.4442A>G (p.Asp1481Gly) | Missense | 0.009030 | 0.006 | B (PP2, BA1, BS2, BP6) | 7870, 7878, 7971 |
|  | NM_001174116.3 | c.4379C>G (p.Thr1460Arg) | Missense | 0.000014 | 0.002 | VUS (PM2, PP2, BP4) | 8115 |
|  | NM_001174116.3 | c.4540C>G (p.Leu1514Val) | Missense | - | 0.002 | VUS (PM2, PP2) | 8304 |
|  | NM_001174116.3 | c.4670C>T (p.Ser1557Leu) | Missense | 0.000016 | 0.004 | VUS (PM2, PP2) | 7680, 8178 |
|  | NM_001174116.3 | c.4987G>A (p.Ala1663Thr) | Missense | 0.000078 | 0.002 | VUS (PM2, PP2) | 7929 |
|  | NM_001174116.3 | c.7454A>G (p.Asp2485Gly) | Missense | 0.002658 | 0.006 | B (PP2, BA1, BS2, BP4, BP6) | 7675, 7804, 7828 |
|  | NM_001174116.3 | c.8065G>A (p.Asp2689Asn) | Missense | - | 0.002 | VUS (PM2, PP2) | 8184 |
|  | NM_001174116.3 | c.8428G>C (p.Val2810Leu) | Missense | - | 0.002 | VUS (PM2, PP2) | 8003 |
|  | NM_001174116.3 | c.8770C>A (p.His2924Asn) | Missense | 0.000152 | 0.002 | LB (PP2, BS1, BP4) | 8041 |
| *DUSP6* | NM_001946.2 | c.11C>G (p.Thr4Arg) | Missense | 0.000022 | 0.002 | VUS (PM2, BP4) | 6908 |
|  | NM_001946.2 | c.1037C>T (p.Thr346Met) | Missense | 0.000286 | 0.002 | VUS (PM2, BP6) | 8112 |
| *EBF2* | NM_022659.4 | c.1513G>A (p.Gly505Ser) | Missense | 0.000071 | 0.002 | VUS (PM2, BP4) | 7686 |
|  | NM_022659.4 | c.1537_1539delTCA (p.Ser513del) | Inframe deletion | 0.001882 | 0.002 | VUS (PM2, BS2, PM4) | 7868 |
| *EDNRB* | NM_000115.4 | c.167A>C (p.Lys56Thr) | Missense | 0.000029 | 0.002 | VUS (PM2) | 7916 |
|  | NM_000115.4 | c.169G>A (p.Gly57Ser) | Missense | 0.005143 | 0.002 | LB (BP4, BP6) | 7814 |
|  | NM_000115.4 | c.778G>T (p.Val260Phe) | Missense | 0.000851 | 0.002 | B (BS1, BS2, BP4, BP6) | 8051 |
| *EGF* | NM_001963.5 | c.1789A>G (p.Ile597Val) | Missense | 0.001118 | 0.002 | B (BS2, BP6) | 5160 |
| *EGF* | NM_001963.5 | c.3251C>G (p.Ala1084Gly) | Missense | 0.001373 | 0.004 | B (BS2, BP4, BP6) | 7637, 8234 |
| *EGFR* | NM_005228.3 | c.635A>G (p.Lys212Arg) | Missense | - | 0.002 | VUS (PM2, BP4) | 5557 |
|  | NM_005228.3 | c.671G>A (p.Arg224His) | Missense | 0.000032 | 0.002 | VUS (PM2) | 8111 |
|  | NM_005228.3 | c.3629C>T (p.Ala1210Val) | Missense | 0.000304 | 0.002 | VUS (PM2) | 8002 |
| *EPHA5* | NM_004439.5 | c.1480C>T (p.Arg494Cys) | Missense | 0.000199 | 0.006 | VUS (PM2) | 5546, 5557, 7712 |
|  | NM_004439.5 | c.2134G>A (p.Gly712Ser) | Missense | - | 0.002 | VUS (PM2) | 8304 |
|  | NM_004439.5 | c.2726A>G (p.Gln909Arg) | Missense | - | 0.002 | VUS (PM2) | 7728 |
| *ERBB4* | NM_005235.2 | c.890T>G (p.Phe297Cys) | Missense | - | 0.002 | VUS (PM2, PP3) | 7850 |
|  | NM_005235.2 | c.1395C>A (p.Asn465Lys) | Missense | 0.000085 | 0.002 | VUS (PM2) | 8030 |
| *FEZF1* | NM_001024613.3 | c.727T>C (p.Phe243Leu) | Missense | - | 0.002 | VUS (PM2) | 7814 |
|  | NM_001024613.3 | c.752G>T (p.Gly251Val) | Missense | - | 0.002 | VUS (PM2, BP4) | 8014 |
| *FGF8* | NM_033163.3 | c.77C>T (p.Pro26Leu) | Missense | 0.001149 | 0.002 | VUS (PP2, BS1, BP6) | 8253 |
|  | NM_033163.3 | c.652C>A (p.Leu218Met) | Missense | - | 0.002 | VUS (PM2, PP2) | 7821 |
|  | NM_033163.3 | c.693C>A (p.Ser231Arg) | Missense | - | 0.002 | VUS (PM2, PP2, BP4) | 7836 |
| *FGFR1* | NM_023110.2 | c.266A>G (p.Gln89Arg) | Missense | 0.000048 | 0.004 | LB (PM2, PP2, BP4, BP6) | 7742, 8245 |
|  | NM_023110.2 | c.584A>G (p.Lys195Arg) | Missense | 0.000016 | 0.002 | VUS (PM2, PP2, BP4) | 8078 |
|  | NM_023110.2 | c.1368G>T (p.Met456Ile) | Missense | 0.000409 | 0.002 | VUS (PP2, PP3, BP6) | 7878 |
|  | NM_023110.2 | c.2314C>T (p.Pro772Ser) | Missense | 0.004194 | 0.004 | B (PP2, BA1, BS2, BP6) | 7637, 7850 |
|  | NM_023110.2 | c.2464C>T (p.Arg822Cys) | Missense | 0.000256 | 0.002 | VUS (PP2, BP6) | 7835 |
| *FLRT3* | NM_198391.2 | c.596C>G (p.Thr199Ser) | Missense | 0.000014 | 0.002 | VUS (PM2, BP4) | 8177 |
|  | NM_198391.2 | c.1255A>T (p.Thr419Ser) | Missense | 0.000110 | 0.002 | VUS (PM2, BP4) | 8203 |
| *FSHB* | NM_000510.2 | c.59G>T (p.Ser20Ile) | Missense | 0.002118 | 0.006 | LB (PM2, BS2, BP6) | 5158, 7993, 8097 |
|  | NM_000510.2 | c.327C>A (p.Ser109Arg) | Missense | 0.000769 | 0.002 | VUS (PM2, BS2) | 7836 |
| *FSTL5* | NM_020116.5 | c.51G>C (p.Glu17Asp) | Missense | 0.002928 | 0.006 | LB (PM2, BS2, BP4) | 7862, 7986, 8112 |
|  | NM_020116.5 | c.447A>T (p.Lys149Asn) | Missense | 0.000260 | 0.002 | VUS (PM2) | 7742 |
|  | NM_020116.5 | c.517A>G (p.Ile173Val) | Missense | 0.000605 | 0.002 | LB (PM2, BS2, BP4) | 7764 |
|  | NM_020116.5 | c.871A>T (p.Asn291Tyr) | Missense | 0.002126 | 0.002 | VUS (PM2, BS2) | 7821 |
|  | NM_020116.5 | c.1358T>C (p.Met453Thr) | Missense | 0.001177 | 0.002 | VUS (PM2) | 7631 |
|  | NM_020116.5 | c.2029G>A (p.Val677Ile) | Missense | - | 0.002 | VUS (PM2, BP4) | 8057 |
| *GADL1* | NM_207359.3 | c.8G>A (p.Ser3Asn) | Missense | 0.000071 | 0.002 | VUS (PM2, BP4) | 5540 |
|  | NM_207359.3 | c.473C>T (p.Ala158Val) | Missense | 0.000053 | 0.002 | VUS (PM2, BP4) | 8181 |
|  | NM_207359.3 | c.620C>T (p.Ser207Leu) | Missense | 0.000805 | 0.002 | LB (PM2, BS2, BP4) | 7837 |
|  | NM_207359.3 | c.1114G>T (p.Asp372Tyr) | Missense | 0.001477 | 0.002 | VUS (BS2) | 8045 |
|  | NM_207359.3 | c.1234G>T (p.Ala412Ser) | Missense | 0.000004 | 0.004 | VUS (PM2) | 7952, 8245 |
|  | NM_207359.3 | c.1312G>A (p.Ala438Thr) | Missense | 0.004121 | 0.008 | B (BS1, BS2, BP4) | 6055, 6058, 7821, 7971 |
| *GH1* | NM_000515.3 | c.7A>C (p.Thr3Pro) | Missense | 0.000238 | 0.002 | VUS (PM2, BS2) | 8112 |
|  | NM_000515.3 | c.134G>A (p.Arg45His) | Missense | 0.000187 | 0.002 | VUS (BS2) | 7687 |
|  | NM_000515.3 | c.493T>C (p.Phe165Leu) | Missense | 0.000103 | 0.002 | VUS | 8189 |
| *GHR* | NM_000163.4 | c.25A>G (p.Thr9Ala) | Missense | 0.000032 | 0.002 | VUS (PM2) | 8026 |
|  | NM_000163.4 | c.679G>C (p.Glu227Gln) | Missense | - | 0.002 | VUS (PM2, PP3) | 7694 |
|  | NM_000163.4 | c.968A>G (p.Asn323Ser) | Missense | 0.000515 | 0.002 | B (BA1, BP4, BP6) | 8164 |
|  | NM_000163.4 | c.1156C>T (p.Arg386Cys) | Missense | 0.000336 | 0.002 | VUS (BS4) | 6056 |
|  | NM_000163.4 | c.1463C>T (p.Ala488Val) | Missense | 0.000103 | 0.002 | VUS (PM2) | 7861 |
| *GJB2* | NM_004004.6 | c.11G>A (p.Gly4Asp) | Missense | 0.000463 | 0.002 | B (PP2, BS1, BP6) | 8253 |
|  | NM_004004.6 | c.88A>G (p.Ile30Val) | Missense | 0.000044 | 0.002 | LP (PM1, PM2, PM5, PP2, PP3) | 8130 |
|  | NM_004004.6 | c.101T>C (p.Met34Thr) | Missense | 0.008996 | 0.008 | LP (PM1, PM2, PM5, PP2, PP3, PP5) | 7644, 7736, 7827, 7862 |
|  | NM_004004.6 | c.478G>A (p.Gly160Ser) | Missense | 0.000602 | 0.002 | LB (PP2, PP3, BS1, BP6) | 8054 |
|  | NM_004004.6 | c.663G>C (p.Lys221Asn) | Missense | 0.000043 | 0.002 | VUS (PM2, PP2) | 7656 |
|  | NM_004004.6 | c.670A>C (p.Lys224Gln) | Missense | 0.000129 | 0.002 | VUS (PM2, PP2) | 8212 |
| *GLI2* | NM_005270.4 | c.8C>T (p.Thr3Met) | Missense | 0.000032 | 0.002 | VUS (PM2, BP4) | 7632 |
|  | NM_005270.4 | c.188C>T (p.Pro63Leu) | Missense | 0.000042 | 0.002 | VUS (PM2, PP3) | 7906 |
|  | NM_005270.4 | c.592G>A (p.Gly198Arg) | Missense | 0.000050 | 0.002 | VUS (PM2) | 7636 |
|  | NM_005270.4 | c.803C>T (p.Ala268Val) | Missense | 0.000822 | 0.002 | B (BA1, BP6) | 7851 |
|  | NM_005270.4 | c.1294G>A (p.Val432Met) | Missense | 0.001542 | 0.004 | B (BA1, BS2, BP4, BP6) | 7850, 8234 |
|  | NM_005270.4 | c.1445A>C (p.Lys482Thr) | Missense | 0.000032 | 0.002 | VUS (PM2, PP3) | 7904 |
|  | NM_005270.4 | c.2159G>A (p.Arg720His) | Missense | 0.000429 | 0.004 | LB (BS1, BP6) | 8005, 8093 |
|  | NM_005270.4 | c.3281C>T (p.Ser1094Phe) | Missense | - | 0.002 | VUS (PM2, BP4) | 8131 |
|  | NM_005270.4 | c.3528G>T (p.Gln1176His) | Missense | 0.000690 | 0.002 | B (BA1, BP6) | 7667 |
|  | NM_005270.4 | c.3623G>A (p.Arg1208His) | Missense | 0.000229 | 0.002 | LB (BP4, BP6) | 5147 |
|  | NM_005270.4 | c.3626G>T (p.Ser1209Ile) | Missense | 0.000145 | 0.002 | LB (BS1, BP4, BP6) | 7837 |
|  | NM_005270.4 | c.3721A>C (p.Met1241Leu) | Missense | 0.000111 | 0.002 | VUS (PM2, BP4) | 7916 |
|  | NM_005270.4 | c.4018G>C (p.Val1340Leu) | Missense | 0.000033 | 0.002 | VUS (PM2, BP4) | 8282 |
|  | NM_005270.4 | c.4054A>G (p.Met1352Val) | Missense | 0.008556 | 0.008 | B (BA1, BS2, BP4, BP6) | 7632, 7641, 7656, 7712 |
|  | NM_005270.4 | c.4558G>A (p.Asp1520Asn) | Missense | 0.009183 | 0.008 | B (BA1, BS2, BP6) | 7632, 7641, 7656, 7712 |
|  | NM_005270.4 | c.4628G>A (p.Arg1543His) | Missense | 0.000534 | 0.002 | VUS (BP6) | 8238 |
| *GLI3* | NM_000168.5 | c.245G>A (p.Arg82Lys) | Missense | 0.000014 | 0.002 | VUS (PM2, BP6) | 7791 |
|  | NM_000168.5 | c.314G>A (p.Arg105His) | Missense | 0.000020 | 0.002 | VUS (PM2) | 8093 |
|  | NM_000168.5 | c.538C>T (p.Arg180Trp) | Missense | 0.000008 | 0.002 | VUS (PM2, PM5) | 7863 |
|  | NM_000168.5 | c.557C>A (p.Ser186Tyr) | Missense | - | 0.002 | VUS (PM2) | 8095 |
|  | NM_000168.5 | c.1354C>A (p.Gln452Lys) | Missense | - | 0.002 | VUS (PM2, PP3) | 8195 |
|  | NM_000168.5 | c.1471T>C (p.Phe491Leu) | Missense | 0.000018 | 0.002 | VUS (PM2, PP3, BP6) | 7682 |
|  | NM_000168.5 | c.2006C>T (p.Thr669Ile) | Missense | 0.000180 | 0.002 | LB (BS1, BP4, BP6) | 7868 |
|  | NM_000168.5 | c.2395C>A (p.Pro799Thr) | Missense | - | 0.002 | VUS (PM2, BP4) | 6048 |
|  | NM_000168.5 | c.2424A>G (p.Ile808Met) | Missense | 0.001937 | 0.004 | B (BS1, BS2, BP6) | 7844, 8306 |
|  | NM_000168.5 | c.2887G>A (p.Ala963Thr) | Missense | 0.000024 | 0.002 | VUS (PM2, BP4) | 6906 |
|  | NM_000168.5 | c.3001G>A (p.Gly1001Ser) | Missense | 0.000586 | 0.004 | B (BS1, BP4, BP6) | 7676, 7923 |
|  | NM_000168.5 | c.3598C>G (p.His1200Asp) | Missense | 0.000963 | 0.002 | B (BA1, BS2, BP6) | 5158 |
|  | NM_000168.5 | c.3664C>T (p.Pro1222Ser) | Missense | 0.002656 | 0.004 | B (BS1, BS2, BP4, BP6) | 5160 |
|  | NM_000168.5 | c.3935T>G (p.Met1312Arg) | Missense | 0.000316 | 0.002 | B (BS1, BS2, BP4, BP6) | 7729 |
| *GNRH1* | NM_000825.3 | c.153G>C (p.Glu51Asp) | Missense | 0.001482 | 0.004 | LB (PM2, BS2, PP3, BP6) | 7920, 8126 |
| *GNRHR* | NM_000406.2 | c.317A>G (p.Gln106Arg) | Missense | 0.002749 | 0.004 | P (PM1, PP2, PM2, PP5) | 7845, 8238 |
|  | NM_000406.2 | c.436C>T (p.Pro146Ser) | Missense | 0.001237 | 0.002 | LB (PM1, PP2, PP3, BS1, BS2) | 5156 |
| *HDAC8* | NM_018486.2 | c.20C>G (p.Pro7Arg) | Missense | 0.000068 | 0.002 | B (PM2, BS2, BP4, BP6) | 8306 |
| *HESX1* | NM_003865.2 | c.102C>G (p.Asp34Glu) | Missense | 0.000012 | 0.002 | VUS (PM2, PP2) | 7889 |
|  | NM_003865.2 | c.134G>T (p.Trp45Leu) | Missense | 0.000012 | 0.002 | VUS (PM2, PP2, PP3) | 7889 |
|  | NM_003865.2 | c.385G>A (p.Val129Ile) | Missense | 0.000791 | 0.002 | LB (PP2, BS1, BS2) | 5477 |
| *HFE* | NM_000410.3 | c.18G>C (p.Arg6Ser) | Missense | 0.000684 | 0.002 | VUS | 7949 |
|  | NM_000410.3 | c.50C>T (p.Thr17Ile) | Missense | 0.000444 | 0.002 | LB (BS2, BP4) | 5470 |
|  | NM_000410.3 | c.884T>C (p.Val295Ala) | Missense | 0.000793 | 0.002 | LB (BS1, BP6) | 7952 |
| *HGF* | NM_000601.4 | c.1787C>T (p.Thr596Met) | Missense | 0.000060 | 0.002 | VUS (PM2) | 5547 |
| *HJV* | NM_213653.3 | c.46A>C (p.Ser16Arg) | Missense | - | 0.002 | VUS (PM2, PP2) | 7633 |
|  | NM_213653.3 | c.143C>G (p.Ser48Trp) | Missense | - | 0.002 | VUS (PM2, PP2, BP4) | 8168 |
|  | NM_213653.3 | c.1003C>T (p.Arg335Trp) | Missense | 0.000011 | 0.002 | VUS (PM2, PP2) | 7806 |
|  | NM_213653.3 | c.1246T>C (p.Ser416Pro) | Missense | 0.000057 | 0.002 | VUS (PP2) | 7644 |
| *HS6ST1* | NM_004807.2 | c.917G>A (p.Arg306Gln) | Missense | 0.000694 | 0.002 | VUS (PM2, PM5, PP3) | 5557 |
|  | NM_004807.2 | c.1124G>A (p.Arg375His) | Missense | 0.000212 | 0.002 | VUS (PM2) | 7945 |
| *IFT172* | NM_015662.2 | c.178A>G (p.Met60Val) | Missense | 0.000012 | 0.002 | VUS (PM2, BP4) | 8075 |
|  | NM_015662.2 | c.649C>T (p.Arg217Trp) | Missense | 0.000032 | 0.002 | VUS (PM2) | 8005 |
|  | NM_015662.2 | c.704G>A (p.Ser235Asn) | Missense | - | 0.002 | VUS (PM2) | 8198 |
|  | NM_015662.2 | c.1056C>G (p.His352Gln) | Missense | 0.000042 | 0.002 | VUS (PM2, BP4) | 8152 |
|  | NM_015662.2 | c.1243G>A (p.Gly415Arg) | Missense | 0.000024 | 0.002 | VUS (PM2) | 5160 |
|  | NM_015662.2 | c.1426G>A (p.Gly476Ser) | Missense | 0.000209 | 0.002 | VUS (PM2) | 7835 |
|  | NM_015662.2 | c.1513C>T (p.Arg505Trp) | Missense | 0.000074 | 0.002 | VUS (BS2) | 7116 |
|  | NM_015662.2 | c.1685C>G (p.Thr562Ser) | Missense | 0.006290 | 0.006 | B (BA1, BS2, BP6) | 7853, 8115, 5473 |
|  | NM_015662.2 | c.2026G>A (p.Gly676Arg) | Missense | 0.000024 | 0.002 | VUS (PM2) | 8093 |
|  | NM_015662.2 | c.2551G>T (p.Val851Leu) | Missense | 0.000875 | 0.002 | B (BA1, BS2, BP4, BP6) | 7851 |
|  | NM_015662.2 | c.2597A>G (p.Gln866Arg) | Missense | 0.000142 | 0.002 | LB (BS1, BP4, BP6) | 7820 |
|  | NM_015662.2 | c.2691G>T (p.Lys897Asn) | Missense | - | 0.002 | VUS (PM2, BP4) | 7992 |
|  | NM_015662.2 | c.2858G>A (p.Arg953His) | Missense | 0.002376 | 0.002 | B (BA1, BS2, BP4, BP6) | 7943 |
|  | NM_015662.2 | c.3073C>G (p.Pro1025Ala) | Missense | 0.001276 | 0.002 | B (BA1, BS1, BS2, BP4, BP6) | 7826 |
|  | NM_015662.2 | c.3610G>C (p.Val1204Leu) | Missense | 0.000901 | 0.002 | B (BA1, BS2, BP4, BP6) | 7851 |
|  | NM_015662.2 | c.4147G>A (p.Glu1383Lys) | Missense | 0.000213 | 0.002 | VUS | 7806 |
|  | NM_015662.2 | c.4363C>T (p.Arg1455Trp) | Missense | 0.000322 | 0.002 | VUS | 7833 |
|  | NM_015662.2 | c.4745T>C (p.Ile1582Thr) | Missense | 0.007594 | 0.004 | B (BA1, BS2, BP4, BP6) | 5480, 5546 |
|  | NM_015662.2 | c.4933G>A (p.Val1645Ile) | Missense | 0.000653 | 0.002 | B (BA1, BP4, BP6) | 8208 |
| *IGSF1* | NM_001170961.1 | c.314G>A (p.Arg105Gln) | Missense | 0.000076 | 0.002 | LB (PM2, BS2, BP6) | 7945 |
|  | NM_001170961.1 | c.929A>T (p.Asp310Val) | Missense | 0.000142 | 0.002 | LB (PM2, BS2, BP6) | 7722 |
|  | NM_001170961.1 | c.1184T>G (p.Leu395Arg) | Missense | 0.009223 | 0.007 | B (BA1, BS2, BP4, BP6) | 8237, 7904, 8118 |
|  | NM_001170961.1 | c.2807C>T (p.Thr936Ile) | Missense | 0.000006 | 0.002 | VUS (PM2, BP4) | 8047 |
|  | NM_001170961.1 | c.2954T>C (p.Val985Ala) | Missense | 0.008812 | 0.005 | B (BA1, BS2, BP6) | 6058, 7904 |
|  | NM_001170961.1 | c.3243G>C (p.Met1081Ile) | Missense | 0.003153 | 0.007 | B (BA1, BS2, BP4, BP6) | 7689, 7956, 8152 |
| *IGSF10* | NM_178822.4 | c.187A>C (p.Asn63His) | Missense | - | 0.002 | VUS (PM2) | 5153 |
|  | NM_178822.4 | c.353G>A (p.Arg118Gln) | Missense | 0.000878 | 0.002 | LB (PM2, BS2, BP4, BP6) | 7914 |
|  | NM_178822.4 | c.467G>T (p.Arg156Leu) | Missense | 0.000354 | 0.002 | VUS (PM2, PP5) | 8018 |
|  | NM_178822.4 | c.620A>G (p.Tyr207Cys) | Missense | 0.000110 | 0.002 | VUS (PM2) | 7814 |
|  | NM_178822.4 | c.823G>A (p.Ala275Thr) | Missense | 0.000131 | 0.002 | VUS (PM2, BP4) | 7821 |
|  | NM_178822.4 | c.1420A>G (p.Met474Val) | Missense | 0.000028 | 0.004 | VUS (PM2, BP4) | 5485, 7911 |
|  | NM_178822.4 | c.1792A>G (p.Thr598Ala) | Missense | 0.000004 | 0.002 | VUS (PM2, BP4) | 8278 |
|  | NM_178822.4 | c.2126G>T (p.Gly709Val) | Missense | 0.000325 | 0.002 | LB (PM2, BS2, BP6) | 7680 |
|  | NM_178822.4 | c.2161T>G (p.Tyr721Asp) | Missense | - | 0.002 | VUS (PM2, BP4) | 8072 |
|  | NM_178822.4 | c.2165G>A (p.Arg722Gln) | Missense | 0.001917 | 0.002 | B (BS1, BS2, BP4, BP6) | 8075 |
|  | NM_178822.4 | c.2204G>A (p.Arg735Gln) | Missense | 0.000050 | 0.002 | VUS (PM2, BP4) | 7680 |
|  | NM_178822.4 | c.2210_2211delTT (p.Phe737*) | Frameshift | 0.000736 | 0.004 | VUS (PM2, BP6) | 5471, 7845 |
|  | NM_178822.4 | c.2359C>T (p.Pro787Ser) | Missense | - | 0.002 | VUS (PM2, BP4) | 7764 |
|  | NM_178822.4 | c.2360C>T (p.Pro787Leu) | Missense | 0.000693 | 0.002 | LB (PM2, BS2, BP4) | 7663 |
|  | NM_178822.4 | c.2852C>A (p.Thr951Lys) | Missense | 0.006092 | 0.006 | B (BS2, BP4, BP6) | 7029, 7805, 8042 |
|  | NM_178822.4 | c.2942C>T (p.Thr981Met) | Missense | 0.000735 | 0.002 | LB (PM2, BS, BP6) | 7680 |
|  | NM_178822.4 | c.3712A>G (p.Thr1238Ala) | Missense | 0.006200 | 0.002 | B (PM2, BS2, BP4, BP6) | 8115 |
|  | NM_178822.4 | c.3797C>G (p.Thr1266Ser) | Missense | 0.001642 | 0.004 | LB (PM2, BP4, BP6) | 5555, 7956 |
|  | NM_178822.4 | c.3856A>G (p.Lys1286Glu) | Missense | 0.000693 | 0.002 | LB (PM2, BP4, BP6) | 8296 |
|  | NM_178822.4 | c.3941C>T (p.Thr1314Met) | Missense | 0.000138 | 0.002 | VUS (PM2, BP4) | 5575 |
|  | NM_178822.4 | c.4187C>T (p.Ser1396Phe) | Missense | 0.000233 | 0.006 | VUS (PM2, BP4) | 5147, 7828, 7993 |
|  | NM_178822.4 | c.4804G>T (p.Glu1602*) | Nonsense | 0.002472 | 0.002 | LB (PM2, BS2, , BP6) | 6077 |
|  | NM_178822.4 | c.5278G>A (p.Gly1760Arg) | Missense | 0.000032 | 0.002 | VUS (PM2, PP3) | 5559 |
|  | NM_178822.4 | c.5983G>A (p.Val1995Ile) | Missense | 0.000772 | 0.002 | VUS (PM2, BS2) | 8195 |
|  | NM_178822.4 | c.6006T>G (p.Phe2002Leu) | Missense | 0.000280 | 0.002 | VUS (PM2, BP4) | 7682 |
|  | NM_178822.4 | c.6245A>G (p.Asn2082Ser) | Missense | 0.000400 | 0.002 | LB (PM, BS2, BP4) | 7680 |
|  | NM_178822.4 | c.6499G>A (p.Gly2167Arg) | Missense | - | 0.002 | VUS (PM2, PP3) | 8023 |
|  | NM_178822.4 | c.7334_7336dupTCA (p.Ile2445dup) | Inframe insertion | 0.000173 | 0.006 | VUS (PM2, PM4) | 7686, 7887, 7904 |
|  | NM_178822.4 | c.7568T>C (p.Ile2523Thr) | Missense | 0.001312 | 0.002 | B (BS2, BP6) | 7850 |
|  | NM_178822.4 | c.7840G>A (p.Asp2614Asn) | Missense | 0.006676 | 0.004 | B (BS1, BS2, BP6) | 6905, 7835 |
| *IL17RD* | NM_017563.4 | c.359C>T (p.Ser120Leu) | Missense | 0.000021 | 0.002 | VUS (PM2) | 7835 |
|  | NM_017563.4 | c.392A>C (p.Lys131Thr) | Missense | 0.000856 | 0.002 | VUS (PM2) | 7764 |
|  | NM_017563.4 | c.481G>A (p.Val161Ile) | Missense | 0.000021 | 0.002 | VUS (PM2) | 8181 |
|  | NM_017563.4 | c.604C>A (p.Pro202Thr) | Missense | - | 0.002 | VUS (PM2) | 8204 |
|  | NM_017563.4 | c.665C>T (p.Pro222Leu) | Missense | 0.000028 | 0.002 | VUS (PM2) | 5554 |
|  | NM_017563.4 | c.964C>T (p.Arg322Cys) | Missense | 0.000049 | 0.002 | VUS (PM2) | 7684 |
|  | NM_017563.4 | c.1570C>G (p.His524Asp) | Missense | 0.000025 | 0.002 | VUS (PM2, BP4) | 8208 |
|  | NM_017563.4 | c.1646T>G (p.Met549Arg) | Missense | 0.000142 | 0.002 | VUS (PM2) | 8025 |
|  | NM_017563.4 | c.1900C>T (p.Arg634Trp) | Missense | 0.000012 | 0.004 | VUS (PM2, BP4) | 7675, 7821 |
| *JAG1* | NM_000214.2 | c.5G>T (p.Arg2Leu) | Missense | 0.000045 | 0.002 | VUS (PM2, PP2, BP4) | 7853 |
|  | NM_000214.2 | c.494G>A (p.Arg165Gln) | Missense | 0.000025 | 0.002 | VUS (PM2, PP2, BP6) | 8018 |
| *KIF14* | NM_014875.2 | c.346G>A (p.Glu116Lys) | Missense | 0.001234 | 0.002 | B (BS1, BP6) | 5575 |
|  | NM_014875.2 | c.397T>C (p.Trp133Arg) | Missense | - | 0.002 | VUS (PM2, BP4) | 7956 |
|  | NM_014875.2 | c.941A>G (p.Lys314Arg) | Missense | 0.001230 | 0.004 | B (BA1, BP4, BP6) | 8199, 8204 |
|  | NM_014875.2 | c.1009G>A (p.Glu337Lys) | Missense | 0.000134 | 0.008 | VUS (PM2, BP4) | 5579, 7853, 8182, 8253 |
|  | NM_014875.2 | c.1087G>A (p.Val363Ile) | Missense | 0.002968 | 0.006 | B (BS1, BS2, BP6) | 6559, 7853, 8182 |
|  | NM_014875.2 | c.1184C>T (p.Thr395Met) | Missense | 0.000750 | 0.002 | B (BA1, BP6) | 7728 |
|  | NM_014875.2 | c.1734G>A (p.Met578Ile) | Missense | 0.003042 | 0.002 | B (BA1, BS2, BP6) | 7923 |
|  | NM_014875.2 | c.2030C>T (p.Thr677Met) | Missense | 0.000040 | 0.002 | VUS (PM2, PP3) | 7861 |
|  | NM_014875.2 | c.2201G>A (p.Arg734Gln) | Missense | 0.003110 | 0.002 | B (BA1, BS2, BP6) | 8045 |
|  | NM_014875.2 | c.2648G>A (p.Arg883His) | Missense | 0.000064 | 0.002 | VUS (PM2, BP4) | 7985 |
|  | NM_014875.2 | c.3181A>G (p.Ile1061Val) | Missense | 0.000078 | 0.002 | VUS (PM2, BP4) | 7844 |
|  | NM_014875.2 | c.3192A>T (p.Glu1064Asp) | Missense | - | 0.002 | VUS (PM2, BP4) | 7945 |
|  | NM_014875.2 | c.3274A>G (p.Met1092Val) | Missense | 0.000456 | 0.002 | B (BS1, BS2, BP4, BP6) | 8003 |
|  | NM_014875.2 | c.3808A>C (p.Ser1270Arg) | Missense | 0.005705 | 0.004 | B (BA1, BS2, BP4, BP6) | 6472, 7884 |
|  | NM_014875.2 | c.4057C>G (p.Gln1353Glu) | Missense | 0.000065 | 0.002 | VUS (PM2) | 8030 |
|  | NM_014875.2 | c.4843G>A (p.Gly1615Ser) | Missense | 0.000573 | 0.002 | LB (BS1, BP4, BP6) | 7688 |
| *KISS1* | NM_002256.3 | c.154_156dupCCG (p.Pro52dup) | Inframe insertion | 0.000030 | 0.002 | VUS (PM2, PM4) | 7923 |
|  | NM_002256.3 | c.244C>A (p.Gln82Lys) | Missense | 0.000161 | 0.004 | VUS (PM2, BP4) | 5540, 8208 |
| *KISS1R* | NM_032551.4 | c.305T>C (p.Leu102Pro) | Missense | 0.000027 | 0.002 | LP (PS4, PS3, PM2) | 7712 |
|  | NM_032551.4 | c.872C>T (p.Ala291Val) | Missense | 0.000013 | 0.004 | VUS (PM2, BP4) | 7852, 7904 |
| *KLB* | NM_175737.3 | c.497T>C (p.Val166Ala) | Missense | 0.001018 | 0.002 | LB (BS2, BP4, BP6) | 7637 |
|  | NM_175737.3 | c.2329_2331delTTC (p.Phe777del) | Inframe deletion | 0.002451 | 0.002 | VUS (PM2, BS2, PM4) | 5473 |
|  | NM_175737.3 | c.2443A>G (p.Lys815Glu) | Missense | 0.000978 | 0.002 | VUS (PM2, BP4) | 8030 |
|  | NM_175737.3 | c.3124G>A (p.Val1042Ile) | Missense | 0.003275 | 0.002 | LB (PM2, BS2, BP4, BP6) | 7845 |
| *LEPR* | NM_002303.5 | c.296G>A (p.Cys99Tyr) | Missense | 0.000100 | 0.002 | VUS (PM2, BP6) | 7904 |
|  | NM_002303.5 | c.371A>G (p.Asp124Gly) | Missense | 0.000714 | 0.004 | B (BA1, BP4, BP6) | 8234, 7995 |
|  | NM_002303.5 | c.1246C>T (p.His416Tyr) | Missense | 0.000295 | 0.002 | VUS | 6077 |
|  | NM_002303.5 | c.2260G>A (p.Val754Met) | Missense | 0.000559 | 0.002 | LB (BS1, BP6) | 8221 |
|  | NM_002303.5 | c.2918C>A (p.Thr973Asn) | Missense | 0.000159 | 0.002 | LB (BS1, BP4, BP6) | 8234 |
| *LHB* | NM_000894.2 | c.239G>A (p.Arg80His) | Missense | 0.000032 | 0.004 | VUS (PM2) | 8075, 8174 |
| *LHX3* | NM_014564.4 | c.400G>A (p.Asp134Asn) | Missense | 0.000050 | 0.002 | VUS (PM2, PP3) | 7663 |
|  | NM_014564.4 | c.748G>A (p.Val250Ile) | Missense | - | 0.002 | VUS (PM2) | 6055 |
|  | NM_014564.4 | c.944G>C (p.Arg315Pro) | Missense | 0.000665 | 0.004 | LB (BS1, BS2) | 5477, 8215 |
|  | NM_014564.4 | c.949G>A (p.Gly317Ser) | Missense | 0.000014 | 0.002 | VUS (PM2, BP4) | 7116 |
| *LHX4* | NM_033343.3 | c.256G>A (p.Gly86Ser) | Missense | 0.000021 | 0.002 | VUS (PM2, PP3) | 5793 |
| *LIF* | NM_002309.3 | c.256G>A (p.Val86Met) | Missense | 0.005179 | 0.002 | B (BS1, BS2, BP4, BP6) | 7992 |
|  | NM_002309.3 | c.415G>A (p.Ala139Thr) | Missense | 0.001771 | 0.002 | B (BS1, BS2, BP4) | 7850 |
|  | NM_002309.3 | c.481G>A (p.Val161Met) | Missense | 0.000014 | 0.002 | VUS (PM2) | 6036 |
| *LZTFL1* | NM_020347.4 | c.26T>C (p.Met9Thr) | Missense | 0.000922 | 0.004 | LB (BS1, BS2, BP4) | 8002, 8296 |
| *MAGEL2* | NM_019066.5 | c.263C>T (p.Pro88Leu) | Missense | 0.000202 | 0.002 | VUS (PM2, BP6) | 7637 |
|  | NM_019066.5 | c.383T>C (p.Leu128Pro) | Missense | 0.002584 | 0.002 | B (BA1, BS2, BP6) | 8045 |
|  | NM_019066.5 | c.1038G>C (p.Arg346Ser) | Missense | 0.001977 | 0.002 | B (BA1, BS2, BP6) | 7820 |
|  | NM_019066.5 | c.1079C>T (p.Ala360Val) | Missense | 0.006048 | 0.006 | B (BA1, BS2, BP6) | 7631, 8030, 8115 |
|  | NM_019066.5 | c.1266_1286dup (p.Ile423_Pro429) | Inframe insertion | 0.000067 | 0.002 | VUS (PM2, PM4) | 8198 |
|  | NM_019066.5 | c.1286C>T (p.Pro429Leu) | Missense | 0.002873 | 0.006 | B (BS1, BS2, BP6) | 7675, 7729, 7844 |
|  | NM_019066.5 | c.1386_1406del (p.Ala463_Pro469del) | Inframe insertion | 0.000457 | 0.002 | B (BS1, BP3, BP6) | 8044 |
|  | NM_019066.5 | c.1720C>G (p.Leu574Val) | Missense | 0.000091 | 0.002 | VUS (BP6) | 7837 |
|  | NM_019066.5 | c.2028G>T (p.Glu676Asp) | Missense | 0.000016 | 0.002 | VUS (PM2) | 7806 |
|  | NM_019066.5 | c.2074G>A (p.Val692Ile) | Missense | 0.003543 | 0.002 | B (BA1, BS2, BP6) | 7634 |
|  | NM_019066.5 | c.2281G>C (p.Ala761Pro) | Missense | 0.000831 | 0.002 | B (BS1, BP6) | 7929 |
|  | NM_019066.5 | c.2290G>A (p.Ala764Thr) | Missense | 0.000710 | 0.006 | B (BS1, BP6) | 7674, 7959, 8239 |
|  | NM_019066.5 | c.2343G>T (p.Glu781Asp) | Missense | 0.000008 | 0.002 | VUS (PM2) | 7860 |
|  | NM_019066.5 | c.2633C>T (p.Pro878Leu) | Missense | 0.001623 | 0.002 | B (BA1, BS2, BP6) | 8044 |
|  | NM_019066.5 | c.2692A>G (p.Ser898Gly) | Missense | 0.001681 | 0.004 | B (BA1, BS2, BP6) | 8131, 8174 |
|  | NM_019066.5 | c.3017C>G (p.Thr1006Ser) | Missense | 0.003454 | 0.002 | B (BA1, BS2, BP6) | 7648 |
|  | NM_019066.5 | c.3464A>C (p.Lys1155Thr) | Missense | 0.000032 | 0.002 | VUS (PM2) | 7689 |
|  | NM_019066.5 | c.3690G>C (p.Glu1230Asp) | Missense | 0.000325 | 0.002 | LB (BS1, BP6) | 8024 |
| *MASTL* | NM_032844.5 | c.34G>C (p.Gly12Arg) | Missense | 0.002931 | 0.002 | B (BS1, BS2, BP4, BP6) | 8189 |
|  | NM_032844.5 | c.70A>G (p.Ile24Val) | Missense | 0.000504 | 0.002 | LB (PM2, BS2, BP4) | 8045 |
|  | NM_032844.5 | c.496A>T (p.Asn166Tyr) | Missense | 0.000032 | 0.002 | VUS (PM2, PP3) | 7745 |
|  | NM_032844.5 | c.1012A>G (p.Met338Val) | Missense | 0.000007 | 0.002 | VUS (PM2, BP4) | 7813 |
|  | NM_032844.5 | c.1016T>A (p.Met339Lys) | Missense | 0.000677 | 0.002 | LB (PM2, BP4, BP6) | 7689 |
|  | NM_032844.5 | c.1828G>A (p.Val610Ile) | Missense | 0.009365 | 0.008 | B (BS2, BP4, BP6) | 6119, 7778, 7860, 8079 |
|  | NM_032844.5 | c.1930A>C (p.Lys644Gln) | Missense | 0.001139 | 0.002 | B (PM2, BS2, BP4, BP6) | 8126 |
|  | NM_032844.5 | c.2051C>T (p.Ser684Leu) | Missense | 0.000020 | 0.002 | VUS (PM2) | 7985 |
| *MC4R* | NM_005912.2 | c.94G>A (p.Gly32Arg) | Missense | - | 0.002 | VUS (PM2, PP2, BP4) | 5579 |
| *MET* | NM_001127500.1 | c.37G>A (p.Val13Met) | Missense | 0.000018 | 0.002 | LB (PM2, BP4, BP6) | 7839 |
|  | NM_001127500.1 | c.305G>A (p.Ser102Asn) | Missense | 0.000008 | 0.002 | VUS (PM2, BP4) | 7820 |
|  | NM_001127500.1 | c.607T>A (p.Ser203Thr) | Missense | 0.000594 | 0.002 | LB (BS1, BS2, BP6) | 6045 |
|  | NM_001127500.1 | c.2962C>T (p.Arg988Cys) | Missense | 0.002843 | 0.004 | B (BA1, BS2, BP6) | 8180, 8232 |
|  | NM_001127500.1 | c.3029C>T (p.Thr1010Ile) | Missense | 0.008249 | 0.004 | B (BA1, BS2, BP6) | 5473, 6559 |
| *MKKS* | NM_018848.3 | c.67A>G (p.Arg23Gly) | Missense | 0.000609 | 0.002 | LB (BS1, BP6) | 7956 |
|  | NM_018848.3 | c.721G>T (p.Val241Leu) | Missense | 0.000012 | 0.002 | VUS (PM2) | 8234 |
|  | NM_018848.3 | c.724G>T (p.Ala242Ser) | Missense | 0.005251 | 0.006 | B (PP3, BA1, BS2, BP6) | 5496, 7920, 8051 |
|  | NM_018848.3 | c.1015A>G (p.Ile339Val) | Missense | 0.004174 | 0.004 | B (BA1, BS2, BP6) | 8128, 8204 |
|  | NM_018848.3 | c.1318C>G (p.Gln440Glu) | Missense | 0.000064 | 0.002 | VUS (PM2) | 5485 |
|  | NM_018848.3 | c.1456T>C (p.Cys486Arg) | Missense | - | 0.002 | VUS (PM2) | 8022 |
|  | NM_018848.3 | c.1474G>A (p.Asp492Asn) | Missense | 0.000318 | 0.002 | VUS | 7906 |
| *MTOR* | NM_004958.3 | c.889G>A (p.Asp297Asn) | Missense | 0.000078 | 0.002 | VUS (PM2, PP2, BP6) | 7970 |
|  | NM_004958.3 | c.1688A>G (p.His563Arg) | Missense | 0.000004 | 0.002 | VUS (PM2, PP2) | 7329 |
|  | NM_004958.3 | c.2323A>G (p.Ile775Val) | Missense | - | 0.002 | VUS (PM2, PP2) | 7906 |
|  | NM_004958.3 | c.3671A>G (p.Asp1224Gly) | Missense | 0.000004 | 0.002 | VUS (PM2, PP2) | 8203 |
|  | NM_004958.3 | c.4885A>C (p.Ile1629Leu) | Missense | 0.000004 | 0.002 | VUS (PM2, PP2) | 6086 |
| *NDNF* | NM_024574.3 | c.239C>T (p.Thr80Met) | Missense | 0.001357 | 0.006 | VUS | 7029, 7806, 8042 |
|  | NM_024574.3 | c.602C>G (p.Thr201Ser) | Missense | 0.001023 | 0.004 | LB (BS2, BP6) | 5491, 7804 |
|  | NM_024574.3 | c.653A>G (p.Glu218Gly) | Missense | 0.000320 | 0.002 | VUS (PM2) | 7935 |
| *NEUROG3* | NM_020999.4 | c.17C>T (p.Ser6Leu) | Missense | - | 0.002 | VUS (PM2) | 8057 |
|  | NM_020999.4 | c.44G>A (p.Arg15His) | Missense | 0.000036 | 0.002 | VUS (PM2, BP6) | 8167 |
|  | NM_020999.4 | c.46G>C (p.Glu16Gln) | Missense | 0.000432 | 0.002 | LB (PM2, BS2, BP6) | 5575 |
|  | NM_020999.4 | c.367G>C (p.Glu123Gln) | Missense | 0.000074 | 0.002 | VUS (PM2, PP3) | 8296 |
| *NHLH2* | NM_005599.3 | c.283T>A (p.Leu95Met) | Missense | 0.000000 | 0.002 | VUS (PM2) | 7952 |
| *NOS1* | NM_000620.4 | c.142C>T (p.Arg48Cys) | Missense | 0.000064 | 0.002 | VUS (PM2, PP2) | 6077 |
|  | NM_000620.4 | c.234C>G (p.Asp78Glu) | Missense | - | 0.002 | VUS (PM2, PP2, BP4) | 6077 |
|  | NM_000620.4 | c.458C>T (p.Ser153Leu) | Missense | 0.002062 | 0.002 | B (PP2, BS1, BS2, BP4, BP6) | 8024 |
|  | NM_000620.4 | c.721G>A (p.Asp241Asn) | Missense | 0.001968 | 0.006 | LB (PM2, BS2, PP2, BP4, BP6) | 7845, 8054, 8265 |
|  | NM_000620.4 | c.1082C>T (p.Pro361Leu) | Missense | 0.000056 | 0.002 | VUS (PM2, PP2, BP4) | 7703 |
|  | NM_000620.4 | c.1181A>C (p.Asp394Ala) | Missense | 0.004755 | 0.002 | B (PM2, BS2, PP2, BP4, BP6) | 5485 |
|  | NM_000620.4 | c.1517T>C (p.Phe506Ser) | Missense | - | 0.002 | VUS (PM2, PP2) | 7635 |
|  | NM_000620.4 | c.2173A>G (p.Asn725Asp) | Missense | 0.002325 | 0.002 | B (PP2, BS1, BS2, BP6) | 8075 |
|  | NM_000620.4 | c.3092A>T (p.Gln1031Leu) | Missense | 0.000004 | 0.002 | VUS (PM2, PP2) | 7805 |
| *NOTCH1* | NM_017617.4 | c.64C>T (p.Pro22Ser) | Missense | 0.002126 | 0.006 | B (PP2, BA1, BS2, BP4, BP6) | 7863, 7911, 8296 |
|  | NM_017617.4 | c.368C>T (p.Thr123Met) | Missense | 0.000907 | 0.004 | B (PP2, BA1, BS1, BS2, BP6) | 8195, 8047 |
|  | NM_017617.4 | c.452A>G (p.Asn151Ser) | Missense | 0.000017 | 0.002 | VUS (PM2, PP2, PP3) | 8023 |
|  | NM_017617.4 | c.701G>A (p.Arg234His) | Missense | 0.000446 | 0.002 | LB (PP2, BS1, BP6) | 8075 |
|  | NM_017617.4 | c.1543G>C (p.Glu515Gln) | Missense | 0.000061 | 0.002 | VUS (PM2, PP2) | 7682 |
|  | NM_017617.4 | c.1682C>T (p.Thr561Met) | Missense | 0.000102 | 0.002 | VUS (PM2, PP2, BP6) | 8126 |
|  | NM_017617.4 | c.2482G>A (p.Val828Met) | Missense | 0.000018 | 0.002 | VUS (PM2, PP2) | 7892 |
|  | NM_017617.4 | c.3190G>A (p.Asp1064Asn) | Missense | 0.000060 | 0.002 | VUS (PM2, PP2, BP6) | 7929 |
|  | NM_017617.4 | c.3767C>T (p.Pro1256Leu) | Missense | 0.002026 | 0.002 | B (PP2, BA1, BS2, BP6) | 8174 |
|  | NM_017617.4 | c.5011G>A (p.Val1671Ile) | Missense | 0.003530 | 0.006 | B (PP2, BA1, BS2, BP4, BP6) | 7116, 7863, 7911 |
|  | NM_017617.4 | c.5273G>A (p.Arg1758His) | Missense | 0.000140 | 0.006 | VUS (PP2, BP6) | 7992, 8126, 8141 |
|  | NM_017617.4 | c.5476G>A (p.Glu1826Lys) | Missense | 0.000028 | 0.002 | VUS (PM2, PP2) | 7895 |
|  | NM_017617.4 | c.5508C>A (p.Asp1836Glu) | Missense | - | 0.002 | VUS (PM2, PP2) | 7883 |
|  | NM_017617.4 | c.6454G>C (p.Gly2152Arg) | Missense | 0.001472 | 0.002 | B (PP2, BA1, BS2, BP4, BP6) | 7820 |
|  | NM_017617.4 | c.6644C>T (p.Ser2215Leu) | Missense | - | 0.002 | VUS (PM2, PP2, PP3) | 7674 |
|  | NM_017617.4 | c.6814C>T (p.Arg2272Cys) | Missense | 0.000016 | 0.002 | VUS (PM2, PP2) | 5147 |
|  | NM_017617.4 | c.6910T>G (p.Leu2304Val) | Missense | 0.000008 | 0.004 | VUS (PM2, PP2, BP6) | 8212, 8233 |
|  | NM_017617.4 | c.7223T>C (p.Leu2408Pro) | Missense | 0.000096 | 0.002 | VUS (PP2, BP6) | 8278 |
|  | NM_017617.4 | c.7397C>G (p.Thr2466Arg) | Missense | - | 0.002 | VUS (PM2, PP2) | 7789 |
|  | NM_017617.4 | c.7498C>G (p.His2500Asp) | Missense | 0.000033 | 0.002 | VUS (PM2, PP2, PP3) | 7993 |
| *NR0B1* | NM_000475.4 | c.16C>A (p.His6Asn) | Missense | 0.001142 | 0.002 | B (BS1, BS2, BP6) | 8047 |
| *NR5A1* | NM_004959.4 | c.523G>A (p.Gly175Ser) | Missense | 0.000009 | 0.002 | VUS (PM2, PP2) | 5160 |
|  | NM_004959.4 | c.754A>T (p.Thr252Ser) | Missense | 0.000012 | 0.002 | VUS (PM1, PM2, PP2, BP4) | 7993 |
|  | NM_004959.4 | c.787G>A (p.Gly263Ser) | Missense | 0.000041 | 0.002 | VUS (PM1, PM2, PP2) | 5470 |
| *NRP1* | NM_003873.7 | c.800G>A (p.Ser267Asn) | Missense | 0.002862 | 0.006 | B (BS1, BS2, BP6) | 5575, 8266, 8278 |
|  | NM_003873.7 | c.1030G>A (p.Ala344Thr) | Missense | 0.000020 | 0.002 | VUS (PM2, PP3) | 5160 |
|  | NM_003873.7 | c.1531T>C (p.Phe511Leu) | Missense | 0.000025 | 0.002 | VUS (PM2, PP3, BS2) | 7852 |
|  | NM_003873.7 | c.1610C>T (p.Ala537Val) | Missense | 0.000014 | 0.002 | VUS (PM2) | 7722 |
|  | NM_003873.7 | c.1775C>T (p.Pro592Leu) | Missense | 0.000016 | 0.002 | VUS (PM2, PP3) | 8131 |
|  | NM_003873.7 | c.2471T>C (p.Ile824Thr) | Missense | 0.000213 | 0.002 | VUS (PM2) | 7894 |
|  | NM_003873.7 | c.2500G>A (p.Glu834Lys) | Missense | 0.000825 | 0.002 | LB (PM2, BP4, BP6) | 7680 |
| *NRP2* | NM_201266.1 | c.292G>T (p.Ala98Ser) | Missense | 0.000012 | 0.002 | VUS (PM2) | 7820 |
|  | NM_201266.1 | c.487C>T (p.Pro163Ser) | Missense | 0.000004 | 0.002 | VUS (PM2) | 7970 |
|  | NM_201266.1 | c.860G>A (p.Arg287Gln) | Missense | 0.001142 | 0.002 | LB (BS2, BP6) | 8024 |
|  | NM_201266.1 | c.1198G>A (p.Ala400Thr) | Missense | 0.000088 | 0.004 | VUS (PM2) | 7793, 8092 |
|  | NM_201266.1 | c.1412G>A (p.Arg471His) | Missense | 0.000174 | 0.002 | VUS (PM2, PP3) | 7878 |
|  | NM_201266.1 | c.2552C>T (p.Ser851Leu) | Missense | 0.000056 | 0.002 | VUS (PM2, BP4) | 8195 |
| *NSMF* | NM_015537.4 | c.241G>A (p.Gly81Ser) | Missense | 0.000157 | 0.004 | VUS (PM2, BP4) | 8003, 8130 |
|  | NM_015537.4 | c.1019G>C (p.Gly340Ala) | Missense | 0.000011 | 0.002 | VUS (PM2, BP4) | 7778 |
|  | NM_015537.4 | c.1091G>A (p.Arg364His) | Missense | 0.000004 | 0.002 | VUS (PM2) | 7689 |
|  | NM_015537.4 | c.1330C>T (p.Arg444Cys) | Missense | 0.000008 | 0.002 | VUS (PM2) | 8026 |
|  | NM_015537.4 | c.1453G>A (p.Val485Ile) | Missense | 0.000039 | 0.002 | VUS (PM2, BP4) | 7804 |
| *NTN1* | NM_004822.2 | c.34_42delCTGGCGGCG (p.Leu12_Ala14del) | Inframe deletion | 0.000090 | 0.002 | VUS (PM2, PM4) | 7684 |
|  | NM_004822.2 | c.116A>G (p.Asp39Gly) | Missense | 0.000015 | 0.002 | VUS (PM2) | 8118 |
|  | NM_004822.2 | c.1121A>G (p.Asn374Ser) | Missense | 0.000021 | 0.002 | VUS (PM2) | 8178 |
|  | NM_004822.2 | c.1289C>T (p.Thr430Met) | Missense | 0.000067 | 0.002 | VUS (PM2) | 7949 |
|  | NM_004822.2 | c.1466A>G (p.Lys489Arg) | Missense | 0.000145 | 0.002 | VUS (PM2) | 7791 |
|  | NM_004822.2 | c.1693C>G (p.Pro565Ala) | Missense | - | 0.002 | VUS (PM2) | 7763 |
| *OTUD4* | NM_001102653.1 | c.386C>G (p.Ala129Gly) | Missense | 0.001292 | 0.002 | LB (PM2, BS2, BP4) | 7663 |
|  | NM_001102653.1 | c.1042C>T (p.Arg348Trp) | Missense | 0.000036 | 0.002 | VUS (PM2) | 7821 |
|  | NM_001102653.1 | c.2755A>G (p.Thr919Ala) | Missense | 0.000004 | 0.002 | VUS (PM2, BP4) | 8044 |
| *PALM2* | NM_001037293.3 | c.464C>G (p.Ser155Cys) | Missense | 0.000004 | 0.002 | VUS (PM2) | 6055 |
|  | NM_001037293.3 | c.818A>G (p.Asn273Ser) | Missense | 0.004110 | 0.008 | B (BS1, BS2, BP4) | 7687, 7789, 7813, 7839 |
|  | NM_001037293.3 | c.979G>A (p.Val327Met) | Missense | 0.000025 | 0.002 | VUS (PM2, BP4) | 8094 |
| *PCSK1* | NM_000439.4 | c.170T>C (p.Leu57Pro) | Missense | - | 0.002 | VUS (PM2) | 7637 |
|  | NM_000439.4 | c.680T>C (p.Val227Ala) | Missense | - | 0.002 | VUS (PM2, PP3) | 5544 |
|  | NM_000439.4 | c.1460T>A (p.Ile487Asn) | Missense | - | 0.002 | VUS (PM2) | 7959 |
|  | NM_000439.4 | c.1549C>T (p.Arg517*) | Nonsense | 0.000012 | 0.002 | P (PVS1, PM2, PM3) | 7894 |
|  | NM_000439.4 | c.1654G>T (p.Asp552Tyr) | Missense | - | 0.002 | VUS (PM2, PP3) | 7688 |
|  | NM_000439.4 | c.1918A>G (p.Thr640Ala) | Missense | 0.001306 | 0.006 | LB (BS1, BP4, BP6) | 7631, 8174, 8178 |
|  | NM_000439.4 | c.2236G>A (p.Val746Met) | Missense | 0.000035 | 0.002 | VUS (PM2, BP4) | 7911 |
| *PDE3A* | NM_000921.4 | c.296A>C (p.Glu99Ala) | Missense | 0.003209 | 0.004 | LB (PM2, BS2, BP4, BP6) | 6051, 8024 |
|  | NM_000921.4 | c.578T>C (p.Val193Ala) | Missense | 0.003176 | 0.004 | LB (PM2, BS2, BP4, BP6) | 5555, 8265 |
|  | NM_000921.4 | c.617T>C (p.Leu206Pro) | Missense | 0.000366 | 0.002 | VUS (PM2) | 8237 |
|  | NM_000921.4 | c.761A>G (p.Tyr254Cys) | Missense | 0.000591 | 0.002 | VUS (PM2) | 7804 |
|  | NM_000921.4 | c.1219T>G (p.Cys407Gly) | Missense | - | 0.002 | VUS (PM2) | 6559 |
|  | NM_000921.4 | c.1375C>T (p.Arg459Trp) | Missense | 0.000011 | 0.002 | VUS (PM1, PM2) | 7934 |
|  | NM_000921.4 | c.1376G>A (p.Arg459Gln) | Missense | 0.001456 | 0.006 | LB (PM2, BS2, BP6) | 7868, 7929, 8014 |
|  | NM_000921.4 | c.3284G>A (p.Arg1095Gln) | Missense | 0.000262 | 0.002 | VUS (PM2, BP4) | 7850 |
| *PGM1* | NM_002633.2 | c.107C>A (p.Ala36Glu) | Missense | 0.000105 | 0.004 | VUS | 7943, 8306 |
|  | NM_002633.2 | c.1103C>T (p.Ala368Val) | Missense | 0.000474 | 0.002 | VUS (BS1) | 7992 |
| *PKNOX2* | NM_001382323.2 | c.210C>G (p.Asp70Glu) | Missense | 0.000004 | 0.002 | VUS (PM2) | 8294 |
|  | NM_001382323.2 | c.846C>A (p.Asp282Glu) | Missense | - | 0.002 | VUS (PM2) | 5157 |
|  | NM_001382323.2 | c.1141G>A (p.Ala381Thr) | Missense | 0.000046 | 0.002 | VUS (PM2) | 7879 |
|  | NM_001382323.2 | c.1339G>A (p.Glu447Lys) | Missense | - | 0.002 | VUS (PM2) | 8296 |
| *PLEKHA5* | NM_001143821.3 | c.890G>C (p.Arg297Thr) | Missense | - | 0.002 | VUS (PM2) | 7870 |
|  | NM_001143821.3 | c.2200G>C (p.Glu734Gln) | Missense | 0.000217 | 0.002 | VUS (PM2) | 6667 |
|  | NM_001143821.3 | c.2339A>G (p.Tyr780Cys) | Missense | - | 0.002 | VUS (PM2) | 7879 |
|  | NM_001143821.3 | c.2404G>A (p.Gly802Ser) | Missense | 0.000214 | 0.002 | VUS (PM2) | 7896 |
|  | NM_001143821.3 | c.2530A>G (p.Met844Val) | Missense | 0.000100 | 0.002 | VUS (PM2, BP4) | 7745 |
|  | NM_001143821.3 | c.3389A>C (p.Glu1130Ala) | Missense | 0.000298 | 0.002 | VUS (PM2) | 7850 |
| *PLXNA1* | NM_032242.3 | c.329G>A (p.Ser110Asn) | Missense | 0.000823 | 0.002 | VUS (PM2, PP2, BP4) | 8233 |
|  | NM_032242.3 | c.649G>A (p.Gly217Ser) | Missense | 0.000035 | 0.002 | VUS (PM2, PP2, BP4) | 8178 |
|  | NM_032242.3 | c.1114C>T (p.Arg372Cys) | Missense | 0.000008 | 0.002 | VUS (PM2, PP2) | 7839 |
|  | NM_032242.3 | c.1474C>T (p.Leu492Phe) | Missense | 0.000011 | 0.002 | VUS (PM2, PP2, BP4) | 7896 |
|  | NM_032242.3 | c.1528G>T (p.Val510Leu) | Missense | 0.000005 | 0.002 | VUS (PM2, PP2, BP4) | 8245 |
|  | NM_032242.3 | c.1832A>G (p.Glu611Gly) | Missense | 0.000012 | 0.004 | VUS (PM2, PP2, BP4) | 5160, 8118 |
|  | NM_032242.3 | c.2261G>A (p.Arg754His) | Missense | 0.000046 | 0.002 | VUS (PM2, PP2, BP4) | 8177 |
|  | NM_032242.3 | c.2690G>A (p.Arg897His) | Missense | 0.000975 | 0.002 | VUS (PM2, PP2, BP4) | 6040 |
|  | NM_032242.3 | c.2908C>T (p.Arg970Cys) | Missense | 0.000004 | 0.002 | VUS (PM2, PP2) | 7820 |
|  | NM_032242.3 | c.3110A>G (p.Asn1037Ser) | Missense | 0.000064 | 0.002 | VUS (PM2, PP2, BP4) | 5155 |
|  | NM_032242.3 | c.3460C>T (p.Pro1154Ser) | Missense | 0.000060 | 0.002 | VUS (PM2, PP2, BP4) | 8237 |
|  | NM_032242.3 | c.3629C>A (p.Ala1210Glu) | Missense | - | 0.002 | VUS (PM2, PP2) | 8005 |
|  | NM_032242.3 | c.3679G>A (p.Glu1227Lys) | Missense | 0.000017 | 0.002 | VUS (PM2, PP2) | 7920 |
|  | NM_032242.3 | c.4903A>G (p.Ser1635Gly) | Missense | 0.001644 | 0.002 | LB (PM2, BS2, PP2, BP6) | 7878 |
| *PLXNA3* | NM_017514.4 | c.85A>G (p.Thr29Ala) | Missense | 0.000006 | 0.002 | VUS (PM2, BP4) | 5579 |
|  | NM_017514.4 | c.265C>T (p.Arg89Cys) | Missense | 0.000016 | 0.002 | LB (PM2, BS2, BP4) | 7959 |
|  | NM_017514.4 | c.719C>T (p.Thr240Met) | Missense | 0.000127 | 0.002 | VUS (PM2, BS2) | 7715 |
|  | NM_017514.4 | c.1150G>A (p.Gly384Ser) | Missense | 0.000894 | 0.002 | B (PM2, BS2, BP6) | 5540 |
|  | NM_017514.4 | c.1204G>A (p.Glu402Lys) | Missense | 0.000067 | 0.002 | VUS (PM2, BS2) | 7960 |
|  | NM_017514.4 | c.1237G>A (p.Gly413Ser) | Missense | 0.000930 | 0.002 | LB (PM2, BS2, BP6) | 7893 |
|  | NM_017514.4 | c.1249G>A (p.Val417Met) | Missense | 0.000162 | 0.002 | VUS (PM2, BS2) | 7820 |
|  | NM_017514.4 | c.1298G>A (p.Arg433His) | Missense | 0.009517 | 0.007 | B (BS1, BS2, BP6) | 8051, 8206, 7722 |
|  | NM_017514.4 | c.1357A>T (p.Thr453Ser) | Missense | - | 0.002 | VUS (PM2, BP4) | 8178 |
|  | NM_017514.4 | c.1493C>T (p.Ala498Val) | Missense | 0.000179 | 0.002 | LB (PM2, BS2, BP4) | 7934 |
|  | NM_017514.4 | c.1628G>A (p.Arg543Gln) | Missense | 0.003025 | 0.005 | B (PM2, BS2, BP4, BP6) | 6472, 7833 |
|  | NM_017514.4 | c.1678G>A (p.Val560Ile) | Missense | 0.000066 | 0.002 | LB (PM2, BS2, BP4) | 7793 |
|  | NM_017514.4 | c.1958C>T (p.Pro653Leu) | Missense | 0.005203 | 0.007 | B (BS2, BP6) | 5463, 8023, 8121 |
|  | NM_017514.4 | c.2268T>G (p.His756Gln) | Missense | 0.000195 | 0.002 | LB (PM2, BS2, BP4) | 7880 |
|  | NM_017514.4 | c.3034A>T (p.Ser1012Cys) | Missense | 0.000097 | 0.002 | VUS (PM2, BS2) | 8174 |
|  | NM_017514.4 | c.3705C>G (p.Ile1235Met) | Missense | - | 0.002 | VUS (PM2) | 8195 |
|  | NM_017514.4 | c.3712G>A (p.Val1238Met) | Missense | 0.000466 | 0.002 | LB (PM2, BS2, BP6) | 8130 |
|  | NM_017514.4 | c.4861G>A (p.Asp1621Asn) | Missense | 0.000016 | 0.002 | VUS (PM2, BS2) | 6040 |
|  | NM_017514.4 | c.5111G>A (p.Arg1704His) | Missense | 0.000033 | 0.002 | LB (PM2, BS2, BP4) | 7632 |
| *PLXNB1* | NM_002673.4 | c.378G>T (p.Gln126His) | Missense | - | 0.002 | VUS (PM2, PP2, BP4) | 7641 |
|  | NM_002673.4 | c.479T>G (p.Leu160Trp) | Missense | - | 0.002 | VUS (PM2, PP2, BP4) | 7728 |
|  | NM_002673.4 | c.655G>A (p.Val219Met) | Missense | 0.001526 | 0.006 | VUS (PM2, BS2, PP2) | 6905, 7675, 7923 |
|  | NM_002673.4 | c.815G>A (p.Arg272His) | Missense | 0.004737 | 0.006 | LB (PP2, BS2, BP4) | 7893, 7985, 8149 |
|  | NM_002673.4 | c.1186C>T (p.Pro396Ser) | Missense | 0.001234 | 0.002 | VUS (PP2) | 8057 |
|  | NM_002673.4 | c.1544C>T (p.Ser515Leu) | Missense | 0.000171 | 0.002 | VUS (PM2, PP2, BP4) | 7964 |
|  | NM_002673.4 | c.2450A>G (p.Asp817Gly) | Missense | 0.000111 | 0.002 | VUS (PM2, PP2, BP4) | 7836 |
|  | NM_002673.4 | c.2512G>T (p.Ala838Ser) | Missense | 0.002461 | 0.002 | B (PP2, BS1, BS2, BP4, BP6) | 8024 |
|  | NM_002673.4 | c.2653C>A (p.Leu885Ile) | Missense | 0.000004 | 0.002 | VUS (PM2, PP2, BP4) | 7792 |
|  | NM_002673.4 | c.2986C>T (p.Arg996Cys) | Missense | 0.000141 | 0.002 | VUS (PM2, PP2, BP4) | 7820 |
|  | NM_002673.4 | c.3911G>A (p.Arg1304His) | Missense | 0.000053 | 0.002 | VUS (PM2, PP2, BP4) | 7964 |
|  | NM_002673.4 | c.5707C>T (p.Leu1903Phe) | Missense | 0.000040 | 0.002 | VUS (PM2, PP2) | 8105 |
| *PNPLA6* | NM_006702.4 | c.1340C>T (p.Pro447Leu) | Missense | 0.004745 | 0.002 | B (PM5, PP2, BA1, BS2, BP4, BP6) | 6091 |
|  | NM_006702.4 | c.3067C>T (p.His1023Tyr) | Missense | 0.000081 | 0.002 | VUS (PM2, PP2) | 7111 |
|  | NM_006702.4 | c.3577C>G (p.Gln1193Glu) | Missense | 0.000149 | 0.002 | VUS (PM2, PP2) | 7878 |
|  | NM_006702.4 | c.3598C>G (p.Gln1200Glu) | Missense | 0.000166 | 0.002 | VUS (PM2, PP2) | 7674 |
|  | NM_006702.4 | c.3611C>T (p.Ala1204Val) | Missense | 0.000028 | 0.002 | VUS (PM2, PP2, BP4) | 8245 |
|  | NM_006702.4 | c.3632G>A (p.Arg1211His) | Missense | 0.000016 | 0.002 | VUS (PM2, PP2) | 6907 |
|  | NM_006702.4 | c.4108G>A (p.Gly1370Ser) | Missense | 0.005078 | 0.004 | B (PP2, BA1, BS2, BP4, BP6) | 7641, 8021 |
| *POGZ* | NM_015100.4 | c.3979A>G (p.Ser1327Gly) | Missense | 0.000018 | 0.002 | VUS (PM2, PP2, BP6) | 6045 |
| *POLA1* | NM_016937.3 | c.437C>T (p.Thr146Ile) | Missense | 0.000032 | 0.002 | LB (PM2, BS2, BP4, BP6) | 7945 |
|  | NM_016937.3 | c.823A>G (p.Lys275Glu) | Missense | - | 0.002 | VUS (PM2, BP4) | 8097 |
|  | NM_016937.3 | c.1345A>G (p.Lys449Glu) | Missense | - | 0.002 | VUS (PM2, BP4) | 8026 |
|  | NM_016937.3 | c.1555A>G (p.Met519Val) | Missense | 0.000073 | 0.002 | B (PM2, BS2, BP4, BP6) | 7793 |
|  | NM_016937.3 | c.3604G>C (p.Asp1202His) | Missense | 0.003915 | 0.005 | B (BA1, BS2, BP6) | 7805, 8184 |
|  | NM_016937.3 | c.3731C>T (p.Thr1244Ile) | Missense | - | 0.002 | VUS (PM2, BP4) | 8306 |
| *POLR1C* | NM_203290.2 | c.34A>G (p.Ser12Gly) | Missense | 0.000020 | 0.002 | VUS (PM2, PP2) | 7959 |
|  | NM_203290.2 | c.193A>G (p.Met65Val) | Missense | 0.000916 | 0.004 | VUS (PP2, PP5, BS1) | 8051, 8239 |
|  | NM_203290.2 | c.421C>T (p.Arg141Cys) | Missense | 0.000619 | 0.006 | LB (PP2, BS1, BP6) | 7934, 7995, 8199 |
| *POLR3A* | NM_007055.3 | c.1177C>T (p.Pro393Ser) | Missense | 0.000004 | 0.002 | VUS (PM2, PP2, PP3) | 7729 |
|  | NM_007055.3 | c.1744C>T (p.Arg582Cys) | Missense | 0.000173 | 0.002 | VUS (PM2, PP2) | 8097 |
|  | NM_007055.3 | c.2938A>G (p.Ile980Val) | Missense | 0.005996 | 0.004 | B (PP2, BS1, BS2, BP4, BP6) | 7880, 8005 |
|  | NM_007055.3 | c.2993G>A (p.Arg998His) | Missense | 0.000120 | 0.002 | VUS (PM2, PP2) | 7896 |
|  | NM_007055.3 | c.3301G>C (p.Val1101Leu) | Missense | 0.000032 | 0.002 | VUS (PM2, PP2) | 8112 |
|  | NM_007055.3 | c.3388G>C (p.Val1130Leu) | Missense | - | 0.002 | VUS (PM2, PP2) | 6667 |
|  | NM_007055.3 | c.3734G>A (p.Arg1245Gln) | Missense | 0.001575 | 0.002 | B (PP2, BS2, BP4, BP6) | 5153 |
|  | NM_007055.3 | c.3840G>A (p.Met1280Ile) | Missense | 0.000014 | 0.002 | VUS (PM2, PP2) | 7641 |
| *POLR3B* | NM_018082.5 | c.432C>G (p.Asn144Lys) | Missense | 0.000007 | 0.002 | VUS (PM2, PP2) | 8232 |
|  | NM_018082.5 | c.469A>G (p.Lys157Glu) | Missense | 0.000008 | 0.002 | VUS (PM2, PP2) | 5160 |
|  | NM_018082.5 | c.1244T>C (p.Met415Thr) | Missense | 0.000566 | 0.002 | LP (PS4, PP2, PP3, BS1) | 5793 |
|  | NM_018082.5 | c.1502A>G (p.His501Arg) | Missense | - | 0.002 | VUS (PM2, PP2, PP3) | 7770 |
|  | NM_018082.5 | c.1931C>T (p.Ala644Val) | Missense | 0.000012 | 0.004 | VUS (PM2, PP2, PP3) | 8072, 8179 |
|  | NM_018082.5 | c.1958A>T (p.Asp653Val) | Missense | 0.006543 | 0.002 | B (PP2, BA1, BS2, BP6) | 5497 |
| *POU6F2* | NM_007252.4 | c.551T>G (p.Leu184Arg) | Missense | 0.000833 | 0.002 | LB (PM2, BS2, BP6) | 6906 |
|  | NM_007252.4 | c.552_553insAG (p.Gln185Serfs*118) | Frameshift | 0.000703 | 0.002 | LP (PVS1, PM2) | 6906 |
|  | NM_007252.4 | c.557T>A (p.Leu186His) | Missense | 0.001374 | 0.002 | LB (PM2, BS2, BP6) | 6906 |
|  | NM_007252.4 | c.734C>T (p.Ala245Val) | Missense | 0.001484 | 0.002 | B (BS2, BP4, BP6) | 7631 |
|  | NM_007252.4 | c.1885A>C (p.Asn629His) | Missense | 0.000440 | 0.002 | VUS (PM2, BS2, PP3) | 8041 |
| *PRDM13* | NM_021620.4 | c.758A>T (p.Glu253Val) | Missense | 0.000011 | 0.002 | VUS (PM2) | 8206 |
|  | NM_021620.4 | c.934T>C (p.Tyr312His) | Missense | 0.000261 | 0.004 | VUS (PM2, BP4) | 7676, 8216 |
|  | NM_021620.4 | c.1102C>G (p.Leu368Val) | Missense | - | 0.002 | VUS (PM2, BP4) | 7929 |
|  | NM_021620.4 | c.1883C>G (p.Thr628Arg) | Missense | - | 0.002 | VUS (PM2) | 7893 |
|  | NM_021620.4 | c.1972C>G (p.Leu658Val) | Missense | 0.000774 | 0.002 | B (PM2, BS2, BP4, BP6) | 7851 |
| *PROKR2* | [NM_144773.3](http://www.ncbi.nlm.nih.gov/entrez/viewer.fcgi?val=NM_000479.3) | c.151G>A (p.Ala51Thr) | Missense | 0.004574 | 0.006 | B (BS1, BS2, BP4, BP6) | 7968, 8131, 8253 |
|  | NM_144773.3 | c.253C>T (p.Arg85Cys) | Missense | 0.000601 | 0.002 | LP (PM1, PM2, PM5, PP5) | 8232 |
|  | [NM_144773.3](http://www.ncbi.nlm.nih.gov/entrez/viewer.fcgi?val=NM_000479.3) | c.518T>G (p.Leu173Arg) | Missense | 0.002196 | 0.004 | LB (BS2, BP6) | 7688, 8184 |
|  | NM_144773.3 | c.1044G>A (p.Met348Ile) | Missense | - | 0.002 | VUS (PM2, BP4) | 7689 |
| *PROP1* | NM_006261.4 | c.301_302delAG (p.Leu102Cysfs*8) | Frameshift | 0.000181 | 0.004 | P (PVS1, PS3, PM2, PM3, PP1) | 7648, 8005 |
|  | NM_006261.4 | c.425C>T (p.Ala142Val) | Missense | 0.001993 | 0.004 | LB (BS1, BP6) | 7880, 8141 |
| *PTCH1* | NM_000264.3 | c.49_51delGGC (p.Gly17del) | Inframe deletion | 0.000835 | 0.006 | LB (PM4, BS1, BP6) | 7805, 7924, 7968 |
|  | NM_000264.3 | c.67G>A (p.Ala23Thr) | Missense | - | 0.002 | VUS (PM2, PP2) | 7684 |
|  | NM_000264.3 | c.1306G>A (p.Asp436Asn) | Missense | 0.000704 | 0.002 | LB (PP2, BS1, BP6) | 7914 |
|  | NM_000264.3 | c.1628G>A (p.Arg543His) | Missense | 0.000016 | 0.002 | VUS (PM2, PP2, PP3, BP6) | 5471 |
|  | NM_000264.3 | c.2015C>T (p.Thr672Met) | Missense | 0.000020 | 0.002 | VUS (PM2, PP2, PP3, BP6) | 7884 |
|  | NM_000264.3 | c.2173C>T (p.Pro725Ser) | Missense | 0.000891 | 0.002 | B (PP2, BS1, BP6) | 7116 |
|  | NM_000264.3 | c.2183C>T (p.Thr728Met) | Missense | 0.002135 | 0.004 | B (PP2, BA1, BS2, BP6) | 7850, 8234 |
|  | NM_000264.3 | c.2689A>G (p.Ile897Val) | Missense | 0.000025 | 0.004 | VUS (PP2, PM2, BP6) | 7111, 8178 |
|  | NM_000264.3 | c.3487G>A (p.Gly1163Ser) | Missense | 0.000513 | 0.002 | VUS (PM5, PP2, PP3, BP6) | 5155 |
|  | NM_000264.3 | c.3845C>T (p.Pro1282Leu) | Missense | 0.002944 | 0.002 | B (PP2, BA1, BS2, BP6) | 8024 |
|  | NM_000264.3 | c.4252G>A (p.Val1418Ile) | Missense | 0.000414 | 0.002 | LB (PP2, BS1, BP6) | 7945 |
|  | NM_000264.3 | c.4324C>T (p.Arg1442Trp) | Missense | 0.001193 | 0.002 | B (PP2, BA1, BS2, BP6) | 7678 |
|  | NM_000264.3 | c.4325G>A (p.Arg1442Gln) | Missense | 0.000411 | 0.002 | B (PP2, BS1, BS2, BP6) | 7827 |
| *RAB18* | NM_021252.3 | c.394G>T (p.Asp132Tyr) | Missense | 0.000085 | 0.002 | VUS (PM2, PP3) | 7862 |
| *RAB3GAP1* | NM_012233.3 | c.244G>A (p.Glu82Lys) | Missense | 0.000004 | 0.002 | VUS (PM2, BP4) | 7703 |
|  | NM_012233.3 | c.913A>G (p.Ile305Val) | Missense | 0.008398 | 0.002 | B (BA1, BS2, BP4, BP6) | 6392 |
|  | NM_012233.3 | c.1006C>T (p.Arg336Cys) | Missense | 0.004542 | 0.006 | B (BA1, BS2, BP6) | 6051, 7840, 7924 |
| *RAB3GAP2* | NM_012414.3 | c.1580C>T (p.Pro527Leu) | Missense | 0.000308 | 0.002 | VUS (PP3, BS1) | 7769 |
|  | NM_012414.3 | c.1657G>A (p.Asp553Asn) | Missense | 0.000106 | 0.002 | VUS (PM2) | 7736 |
|  | NM_012414.3 | c.3355G>A (p.Glu1119Lys) | Missense | - | 0.002 | VUS (PM2, BP4) | 7806 |
|  | NM_012414.3 | c.3991C>A (p.Leu1331Ile) | Missense | 0.000810 | 0.004 | B (BS1, BS2, BP4, BP6) | 7807, 7923 |
|  | NM_012414.3 | c.4060A>G (p.Ile1354Val) | Missense | 0.000870 | 0.008 | B (BS1, BS2, BP4, BP6) | 8045, 8093, 8294, 8304 |
| *RBM28* | NM_018077.2 | c.718G>C (p.Asp240His) | Missense | 0.002576 | 0.004 | B (BS1, BS2, BP4, BP6) | 7863, 8234 |
|  | NM_018077.2 | c.746A>G (p.Asp249Gly) | Missense | 0.000913 | 0.002 | LB (PM2, BS2, BP4) | 8180 |
|  | NM_018077.2 | c.1387A>C (p.Met463Leu) | Missense | 0.001527 | 0.002 | LB (PM2, BP4, BP6) | 7736 |
|  | NM_018077.2 | c.1433A>G (p.Lys478Arg) | Missense | - | 0.002 | VUS (PM2, BP4) | 7878 |
|  | NM_018077.2 | c.2015C>T (p.Ala672Val) | Missense | 0.002074 | 0.004 | B (BS1, BS2, BP4, BP6) | 7863, 8234 |
|  | NM_018077.2 | c.2077C>T (p.His693Tyr) | Missense | 0.002121 | 0.008 | B (PM2, BS2, BP4, BP6) | 5158, 5471, 6086, 7873 |
|  | NM_018077.2 | c.2273A>G (p.Asp758Gly) | Missense | 0.001213 | 0.002 | B (PM2, BS2, BP4, BP6) | 6091 |
| *RD3* | NM_183059.2 | c.16T>C (p.Trp6Arg) | Missense | 0.002725 | 0.002 | B (BA1, BS2, BP6) | 8149 |
|  | NM_183059.2 | c.69G>C (p.Glu23Asp) | Missense | 0.002678 | 0.002 | B (BA1, BS2, BP4, BP6) | 8149 |
|  | NM_183059.2 | c.202C>T (p.Arg68Trp) | Missense | 0.000265 | 0.002 | VUS (BP4) | 6086 |
|  | NM_183059.2 | c.223C>T (p.Pro75Ser) | Missense | 0.000004 | 0.002 | VUS (PM2) | 8051 |
|  | NM_183059.2 | c.584A>T (p.Asp195Val) | Missense | 0.009535 | 0.006 | B (BA1, BS2, BP4, BP6) | 7814, 7894, 8022 |
| *RELN* | NM_005045.3 | c.7C>G (p.Arg3Gly) | Missense | - | 0.002 | VUS (PM2, PP2, BP4) | 7792 |
|  | NM_005045.3 | c.1108G>C (p.Gly370Arg) | Missense | 0.000255 | 0.002 | VUS (PM2, PP2, BP6) | 5153 |
|  | NM_005045.3 | c.2782A>G (p.Ile928Val) | Missense | 0.000004 | 0.002 | VUS (PM2, PP2, BP4) | 7852 |
|  | NM_005045.3 | c.3651C>G (p.Ile1217Met) | Missense | 0.002524 | 0.002 | B (PP2, PP3, BS1, BS2, BP6) | 7722 |
|  | NM_005045.3 | c.4408G>A (p.Val1470Ile) | Missense | 0.002425 | 0.006 | B (PP2, BS1, BS2, BP4, BP6) | 7633, 8216, 8278 |
|  | NM_005045.3 | c.5108C>G (p.Pro1703Arg) | Missense | 0.003513 | 0.002 | B (PP2, BA1, BS2, BP6) | 8014 |
|  | NM_005045.3 | c.5200C>G (p.Leu1734Val) | Missense | 0.000279 | 0.002 | LB (PP2, BS1, BP4, BP6) | 7851 |
|  | NM_005045.3 | c.5345G>A (p.Arg1782His) | Missense | 0.000011 | 0.002 | VUS (PM2, PP2, BP4) | 7685 |
|  | NM_005045.3 | c.5467G>A (p.Ala1823Thr) | Missense | - | 0.002 | VUS (PM2, PP2) | 7641 |
|  | NM_005045.3 | c.5822T>C (p.Val1941Ala) | Missense | 0.000358 | 0.004 | VUS (PP2, BP4) | 6052, 8130 |
|  | NM_005045.3 | c.6770C>T (p.Ser2257Leu) | Missense | 0.000040 | 0.004 | VUS (PP2, PM2) | 5547, 8282 |
|  | NM_005045.3 | c.7114G>A (p.Val2372Met) | Missense | 0.000237 | 0.002 | LB (PP2, BS1, BP6) | 8047 |
|  | NM_005045.3 | c.7438G>A (p.Gly2480Ser) | Missense | 0.002620 | 0.004 | LB (PP2, BS1, BP6) | 7880, 8025 |
|  | NM_005045.3 | c.7634C>T (p.Ala2545Val) | Missense | 0.000216 | 0.004 | VUS (PP2, BP6) | 7964, 7995 |
|  | NM_005045.3 | c.8005G>A (p.Val2669Ile) | Missense | 0.000166 | 0.002 | LB (PM2, PP2, BP4, BP6) | 7949 |
| *RNF216* | NM_207111.4 | c.91C>G (p.Pro31Ala) | Missense | - | 0.002 | VUS (PM2, BP4) | 8149 |
|  | NM_207111.4 | c.230A>G (p.Asn77Ser) | Missense | 0.000089 | 0.002 | VUS (PM2, BP4) | 7715 |
|  | NM_207111.4 | c.854C>T (p.Ser285Leu) | Missense | 0.001577 | 0.002 | B (BS1, BS2, BP4, BP6) | 8025 |
|  | NM_207111.4 | c.1865C>T (p.Thr622Met) | Missense | 0.000014 | 0.002 | VUS (PM2, BP4) | 5559 |
|  | NM_207111.4 | c.2686G>A (p.Val896Ile) | Missense | 0.000495 | 0.002 | B (BA1, BP4, BP6) | 8024 |
| *ROBO3* | NM_022370.3 | c.323G>T (p.Arg108Leu) | Missense | 0.000008 | 0.002 | VUS (PM2) | 5554 |
|  | NM_022370.3 | c.377C>A (p.Pro126Gln) | Missense | 0.000046 | 0.002 | VUS (PM2) | 5470 |
|  | NM_022370.3 | c.1542G>A (p.Met514Ile) | Missense | 0.000511 | 0.002 | B (BA1, BS2, BP4, BP6) | 7637 |
|  | NM_022370.3 | c.1615C>T (p.Arg539Trp) | Missense | 0.003234 | 0.006 | B (BA1, BS2, BP4, BP6) | 5480, 5579, 6545 |
|  | NM_022370.3 | c.2021G>A (p.Arg674His) | Missense | 0.000068 | 0.002 | VUS (PM2) | 8079 |
|  | NM_022370.3 | c.2300C>A (p.Ala767Asp) | Missense | 0.000047 | 0.002 | VUS (PM2, BP4) | 8005 |
|  | NM_022370.3 | c.2461C>G (p.Arg821Gly) | Missense | 0.000013 | 0.002 | VUS (PM2, BP4) | 8045 |
|  | NM_022370.3 | c.2621T>A (p.Leu874Gln) | Missense | 0.005196 | 0.006 | B (BA1, BS2, BP4, BP6) | 7678, 7885, 7895 |
|  | NM_022370.3 | c.2763G>C (p.Glu921Asp) | Missense | 0.001301 | 0.002 | B (BA1, BP6) | 7952 |
|  | NM_022370.3 | c.2899C>T (p.Pro967Ser) | Missense | 0.000128 | 0.002 | VUS (PM2) | 8167 |
|  | NM_022370.3 | c.2993G>T (p.Gly998Val) | Missense | 0.001755 | 0.002 | B (PP3, BS1, BS2, BP6) | 5557 |
|  | NM_022370.3 | c.3271T>C (p.Ser1091Pro) | Missense | 0.000068 | 0.002 | VUS | 7985 |
|  | NM_022370.3 | c.3593G>A (p.Arg1198His) | Missense | 0.007416 | 0.004 | B (BA1, BS2, BP4, BP6) | 5496, 7728 |
|  | NM_022370.3 | c.3836C>T (p.Ala1279Val) | Missense | 0.000240 | 0.002 | LB (BS1, BS2, BP4, BP6) | 6077 |
|  | NM_022370.3 | c.4100_4104delGGAGT (p.Arg1367Profs*29) | Frameshift | 0.002672 | 0.004 | B (PVS1, BA1, BS2, BP6) | 7851, 8296 |
|  | NM_022370.3 | c.4116C>A (p.Ser1372Arg) | Missense | 0.002020 | 0.004 | B (BS1, BS2, BP4, BP6) | 7889, 8232 |
| *SEC14L3* | NM_174975.4 | c.81delT (p.Pro28Leufs*52) | Frameshift | 0.000219 | 0.002 | VUS (PM2) | 7634 |
|  | NM_174975.4 | c.808G>C (p.Val270Leu) | Missense | 0.004186 | 0.004 | B (BS1, BS2, BP4) | 5555, 6052 |
|  | NM_174975.4 | c.1120G>A (p.Ala374Thr) | Missense | 0.000272 | 0.006 | VUS (PM2, BP4) | 6051, 8025, 8177 |
| *SEMA3A* | NM_006080.2 | c.196C>T (p.Arg66Trp) | Missense | 0.000513 | 0.002 | VUS (PM2) | 8051 |
|  | NM_006080.2 | c.271A>G (p.Ile91Val) | Missense | 0.000020 | 0.002 | VUS (PM2) | 6392 |
|  | NM_006080.2 | c.1923G>C (p.Gln641His) | Missense | 0.001140 | 0.002 | LB (BP4, BP6) | 7674 |
| *SEMA3E* | NM_012431.2 | c.142A>G (p.Ile48Val) | Missense | - | 0.002 | VUS (PM2, BP4) | 5156 |
|  | NM_012431.2 | c.1174G>A (p.Gly392Arg) | Missense | 0.000379 | 0.002 | LB (PM2, BP4, BP6) | 7971 |
|  | NM_012431.2 | c.1296C>A (p.Asn432Lys) | Missense | 0.000004 | 0.002 | VUS (PM2, BP4) | 7860 |
| *SEMA4D* | NM_006378.3 | c.1528G>A (p.Glu510Lys) | Missense | 0.000004 | 0.002 | VUS (PM2, BP4) | 6052 |
|  | NM_006378.3 | c.1957G>A (p.Val653Ile) | Missense | 0.000028 | 0.002 | VUS (PM2, BP4) | 7637 |
|  | NM_006378.3 | c.2393G>A (p.Ser798Asn) | Missense | 0.000941 | 0.002 | VUS (PM2, BP4) | 8075 |
| *SEMA7A* | NM_003612.3 | c.290T>A (p.Leu97His) | Missense | 0.001274 | 0.002 | VUS (PM2, BP4) | 8054 |
|  | NM_003612.3 | c.379G>A (p.Glu127Lys) | Missense | 0.000053 | 0.002 | VUS (PM2, BP4) | 5480 |
|  | NM_003612.3 | c.709G>A (p.Asp237Asn) | Missense | 0.000230 | 0.002 | VUS (PM2, BP4) | 7935 |
|  | NM_003612.3 | c.1864C>T (p.Arg622Cys) | Missense | 0.000598 | 0.002 | VUS (PM2, BP4) | 5158 |
|  | NM_003612.3 | c.1865G>A (p.Arg622His) | Missense | 0.000404 | 0.002 | VUS (PM2, BP4) | 7935 |
|  | NM_003612.3 | c.1996C>T (p.His666Tyr) | Missense | - | 0.002 | VUS (PM2, BP4) | 7880 |
| *SIN3A* | NM_001145358.2 | c.17A>G (p.Asp6Gly) | Missense | - | 0.002 | VUS (PM2, PP2, BP4) | 7870 |
|  | NM_001145358.2 | c.836C>T (p.Pro279Leu) | Missense | 0.001935 | 0.002 | B (PP2, BS1, BS2, BP6) | 7845 |
| *SLC29A3* | NM_018344.5 | c.40A>G (p.Asn14Asp) | Missense | 0.000036 | 0.002 | VUS (PM2, BP4) | 6392 |
|  | NM_018344.5 | c.269C>T (p.Thr90Ile) | Missense | 0.000085 | 0.002 | VUS (PM2, BP4) | 7743 |
|  | NM_018344.5 | c.385G>A (p.Val129Ile) | Missense | 0.000068 | 0.002 | VUS (PM2, BP4) | 8198 |
|  | NM_018344.5 | c.488G>T (p.Gly163Val) | Missense | 0.008063 | 0.002 | B (BA1, BS2, BP4, BP6) | 7836 |
|  | NM_018344.5 | c.707C>T (p.Thr236Met) | Missense | 0.000350 | 0.002 | VUS | 5157 |
|  | NM_018344.5 | c.1285C>A (p.Leu429Ile) | Missense | 0.000011 | 0.002 | VUS (PM2) | 6051 |
| *SLIT2* | NM_004787.3 | c.287G>A (p.Arg96Lys) | Missense | 0.000020 | 0.002 | VUS (PM2, PP2, BP4) | 7778 |
|  | NM_004787.3 | c.688G>T (p.Val230Phe) | Missense | 0.000040 | 0.002 | VUS (PM2, PP2) | 7635 |
|  | NM_004787.3 | c.1850G>A (p.Arg617Gln) | Missense | 0.000080 | 0.002 | VUS (PM2, PP2, BP4) | 5496 |
|  | NM_004787.3 | c.2875C>G (p.His959Asp) | Missense | - | 0.002 | VUS (PM2, PP2) | 7958 |
|  | NM_004787.3 | c.3095T>C (p.Leu1032Ser) | Missense | 0.000018 | 0.002 | VUS (PM2, PP2, PP3) | 8057 |
|  | NM_004787.3 | c.3724A>G (p.Ile1242Val) | Missense | 0.000025 | 0.002 | VUS (PM2, PP2, BP4) | 7631 |
|  | NM_004787.3 | c.4049G>A (p.Ser1350Asn) | Missense | 0.003739 | 0.006 | B (PM2, PP2, BS2, BS6) | 5480, 7833, 8051 |
|  | NM_004787.3 | c.4253C>T (p.Ala1418Val) | Missense | 0.002813 | 0.002 | B (PM2, PP2, BS2, BP6) | 7835 |
|  | NM_004787.3 | c.4400G>T (p.Gly1467Val) | Missense | 0.000266 | 0.002 | VUS (PM2, PP2) | 7835 |
| *SMCHD1* | NM_015295.2 | c.23G>A (p.Gly8Glu) | Missense | - | 0.002 | VUS (PM2, PP2, BP4) | 6545 |
|  | NM_015295.2 | c.424C>T (p.Pro142Ser) | Missense | 0.000011 | 0.002 | VUS (PM1, PM2, PP2, PP3) | 8047 |
|  | NM_015295.2 | c.3527C>T (p.Thr1176Ile) | Missense | 0.000304 | 0.002 | VUS (PP2, BP4) | 8238 |
|  | NM_015295.2 | c.5024A>G (p.His1675Arg) | Missense | 0.000139 | 0.002 | LB (PP2, BS1, BP4) | 8045 |
|  | NM_015295.2 | c.5729A>G (p.Gln1910Arg) | Missense | 0.000004 | 0.002 | VUS (PM2, PP2, BP4) | 8024 |
|  | NM_015295.2 | c.5737C>G (p.Arg1913Gly) | Missense | - | 0.002 | VUS (PM2, PP2, BP4) | 5469 |
| *SOX11* | NM_003108.4 | c.1051_1062dupAGCGGCAGCAGC (p.Ser351_Ser354dup) | Inframe insertion | 0.003398 | 0.004 | B (PM4, BA1, BS2, BP6) | 5570, 5793 |
| *SOX3* | NM_005634.2 | c.14G>A (p.Arg5Gln) | Missense | 0.003194 | 0.002 | B (BS2, BP6) | 7920 |
|  | NM_005634.2 | c.1059_1076delCACCGCCGCGGCCGCAGC (p.Thr354_Ala359del) | Inframe deletion | - | 0.002 | VUS (PM2, BP3) | 7924 |
| *SPRY4* | NM_030964.5 | c.722C>A (p.Ser241Tyr) | Missense | 0.004530 | 0.006 | VUS (PM2, BP6) | 7644, 8018, 8111 |
|  | NM_030964.5 | c.841G>A (p.Val281Met) | Missense | 0.000280 | 0.002 | VUS (PM2) | 8054 |
| *SRA1* | NM_001035235.2 | c.184C>A (p.Pro62Thr) | Missense | 0.000004 | 0.002 | VUS (PM2, BP4) | 7885 |
|  | NM_001035235.2 | c.413G>A (p.Gly138Glu) | Missense | 0.002195 | 0.004 | LB (PM2, BS2, BP4, BP6) | 8025, 6086 |
|  | NM_001035235.2 | c.653C>T (p.Pro218Leu) | Missense | 0.001549 | 0.002 | B (BS1, BS2, BP6) | 8045 |
| *STS* | NM_000351.4 | c.1124G>C (p.Gly375Ala) | Missense | 0.000429 | 0.002 | B (PP3, BS1, BS2, BP6) | 6040 |
|  | NM_000351.4 | c.1717G>A (p.Asp573Asn) | Missense | - | 0.002 | VUS (PM2) | 7945 |
| *STUB1* | NM_005861.4 | c.44C>T (p.Ala15Val) | Missense | 0.000078 | 0.002 | VUS (PM2, PP2, BP4) | 6040 |
| *TAC3* | NM_013251.3 | c.153C>G (p.Phe51Leu) | Missense | 0.000004 | 0.002 | VUS (PM2, BP4) | 8130 |
| *TACR3* | NM_001059.2 | c.496A>C (p.Ile166Leu) | Missense | 0.000007 | 0.002 | VUS (PM2) | 5160 |
|  | NM_001059.2 | c.745A>G (p.Ile249Val) | Missense | 0.000142 | 0.002 | VUS (PM2, BP4) | 8105 |
|  | NM_001059.2 | c.918G>A (p.Met306Ile) | Missense | 0.000128 | 0.002 | VUS (PM2, BP4) | 7682 |
|  | NM_001059.2 | c.1345G>A (p.Ala449Thr) | Missense | 0.005288 | 0.006 | B (BA1, BS2, BP4, BP6) | 7885, 8005, 8180 |
| *TBCE* | NM_003193.3 | c.146A>C (p.His49Pro) | Missense | 0.000032 | 0.002 | VUS (PM2, PP3) | 7870 |
|  | NM_003193.3 | c.214C>T (p.Pro72Ser) | Missense | 0.003571 | 0.004 | B (BA1, BS1, BS2, BP6) | 6907, 7826 |
|  | NM_003193.3 | c.1465C>A (p.Leu489Ile) | Missense | 0.000520 | 0.002 | VUS (BP4) | 8216 |
| *TBX3* | NM_005996.3 | c.1052G>T (p.Ser351Ile) | Missense | 0.000023 | 0.002 | VUS (PM2) | 8181 |
|  | NM_005996.3 | c.1242C>G (p.Asp414Glu) | Missense | 0.000008 | 0.002 | VUS (PM2) | 7958 |
|  | NM_005996.3 | c.1783T>G (p.Ser595Ala) | Missense | 0.000664 | 0.002 | B (BS1, BS2, BP6) | 8051 |
| *TCF12* | NM_207036.1 | c.454C>T (p.Pro152Ser) | Missense | 0.000329 | 0.002 | VUS (BP4) | 7743 |
|  | NM_207036.1 | c.1409G>A (p.Ser470Asn) | Missense | 0.000011 | 0.002 | VUS (PM2, BP4) | 7676 |
|  | NM_207036.1 | c.1520T>G (p.Leu507Arg) | Missense | 0.000999 | 0.002 | B (BA1, BS2, BP6) | 7729 |
| *TFR2* | NM_003227.3 | c.840C>G (p.Phe280Leu) | Missense | 0.000397 | 0.002 | VUS | 8235 |
|  | NM_003227.3 | c.1259G>A (p.Arg420His) | Missense | 0.000170 | 0.002 | VUS (PM2) | 8218 |
| *TLE4* | NM_001282748.2 | c.440C>T (p.Pro147Leu) | Missense | - | 0.002 | VUS (PM2, PP2) | 8024 |
|  | NM_001282748.2 | c.494G>C (p.Ser165Thr) | Missense | 0.002545 | 0.002 | LB (PM2, BS2, PP2, BP4) | 8234 |
| *TRAPPC9* | NM_031466.5 | c.29C>G (p.Ala10Gly) | Missense | - | 0.002 | VUS (PM2) | 8105 |
|  | NM_031466.5 | c.140G>A (p.Arg47Gln) | Missense | 0.000315 | 0.004 | VUS (PM2, BP4) | 7821, 8178 |
|  | NM_031466.5 | c.853C>T (p.Arg285Trp) | Missense | 0.000453 | 0.002 | VUS | 8079 |
|  | NM_031466.5 | c.2354T>G (p.Val785Gly) | Missense | 0.000007 | 0.002 | VUS (PM2, BP4) | 8265 |
|  | NM_031466.5 | c.2632C>G (p.Leu878Val) | Missense | 0.000012 | 0.002 | VUS (PM2) | 8164 |
|  | NM_031466.5 | c.2671A>C (p.Thr891Pro) | Missense | 0.000191 | 0.002 | VUS (BS1) | 7736 |
|  | NM_031466.5 | c.3149G>A (p.Arg1050Gln) | Missense | 0.000890 | 0.002 | LB (BS1, BP4, BP6) | 7863 |
| *TRIM32* | NM_012210.3 | c.558G>C (p.Gln186His) | Missense | 0.001857 | 0.002 | LB (BS1, BS2, BP6) | 7329 |
| *TTC8* | NM_198309.3 | c.1219C>T (p.His407Tyr) | Missense | - | 0.004 | VUS (PM2) | 5579, 6052 |
| *TYRO3* | NM_006293.3 | c.1790C>G (p.Pro597Arg) | Missense | 0.000092 | 0.002 | VUS | 7945 |
|  | NM_006293.3 | c.2264C>T (p.Pro755Leu) | Missense | 0.000057 | 0.002 | VUS (PM2) | 7745 |
| *WDPCP* | NM_015910.7 | c.160G>A (p.Asp54Asn) | Missense | 0.000129 | 0.002 | VUS (PM2, PP3, PP5) | 8057 |
|  | NM_015910.7 | c.691A>G (p.Ile231Val) | Missense | 0.000056 | 0.004 | LB (PM2, BP4, BP6) | 5477, 7656 |
|  | NM_015910.7 | c.1079C>T (p.Ser360Leu) | Missense | 0.000171 | 0.002 | VUS (BS1) | 8005 |
|  | NM_015910.7 | c.1263A>C (p.Leu421Phe) | Missense | 0.000504 | 0.002 | LB (BS1, BP4, BP6) | 8206 |
|  | NM_015910.7 | c.1429A>G (p.Lys477Glu) | Missense | - | 0.002 | VUS (PM2) | 8047 |
|  | NM_015910.7 | c.1606C>G (p.Leu536Val) | Missense | 0.000008 | 0.004 | VUS (PM2) | 7630, 7839 |
|  | NM_015910.7 | c.1799delG (p.Arg600Leufs*12) | Frameshift | 0.000012 | 0.002 | P (PVS1, PM2, PP5) | 7923 |
| *WDR11* | NM_018117.11 | c.1449G>C (p.Met483Ile) | Missense | 0.000305 | 0.002 | VUS | 7764 |
|  | NM_018117.11 | c.2305A>G (p.Met769Val) | Missense | 0.000562 | 0.008 | VUS (BS1) | 5555, 7887, 7934, 8018 |
|  | NM_018117.11 | c.2584C>G (p.Gln862Glu) | Missense | - | 0.002 | VUS (PM2, PP3) | 7637 |
|  | NM_018117.11 | c.3571G>A (p.Gly1191Ser) | Missense | 0.000141 | 0.008 | VUS (PM2, PP3) | 5147, 6559, 7840, 7943 |
| *WDR4* | NM_033661.4 | c.586G>A (p.Val196Met) | Missense | 0.000641 | 0.002 | LB (BS2, BP4) | 7663 |
|  | NM_033661.4 | c.1036A>G (p.Met346Val) | Missense | 0.000435 | 0.002 | LB (BS1, BP6) | 7885 |
|  | NM_033661.4 | c.1148T>C (p.Leu383Pro) | Missense | 0.000024 | 0.002 | VUS (PM2) | 7895 |
|  | NM_033661.4 | c.1150G>A (p.Glu384Lys) | Missense | 0.000024 | 0.002 | VUS (PM2, BP4) | 7895 |

GnomAD, Genome Aggregation Database; ACMG, American College of Medical Genetics and Genomics. Variants were classified as Pathogenic (P), Likely Pathogenic (LP), Variants of Uncertain Significance (VUS), Likely Benign (LB), or Benign (B), based on the evidence for pathogenicity [very strong (PVS1), strong (PS1-4), moderate (PM1–6), or supporting (PP1–5)] or benign impact [stand-alone (BA), strong (BS1-4), or supporting (BP1-7)]. id, identification; PMID, PubMed identifier.
