# Supplementary material for: Genetic architecture of congenital hypogonadotropic hypogonadism: insights from analysis of a Portuguese cohort
Source: Hum Reprod Open. 2024 Sep 11;2024(3):hoae053. doi: 10.1093/hropen/hoae053 (PMC11415827; doi:10.1093/hropen/hoae053)
Supplement: hoae053_Supplementary_Data [file hoae053_supplementary_data.zip › Supplementary Table S5.docx]

**Supplementary Table S5.** Characteristics of controls with Pathogenic (P), Likely Pathogenic (LP) and Variants of Uncertain Significance (VUS).

| **Control id** | **Sex** |  | **Variant** | **Zygosity** | **Allele frequency in GnomAD** | **ACMG classification** |
| --- | --- | --- | --- | --- | --- | --- |
| 5147 | M |  | NM_001080414.4(*CCDC88C*):c.2958G>A (p.Met986Ile) | Het | 0.000182 | VUS (PM2, BP4) |
|  |  |  | NM_178822.4(*IGSF10*):c.4187C>T (p.Ser1396Phe) | Het | 0.000233 | VUS (PM2, BP4) |
|  |  |  | NM_017617.4(*NOTCH1*):c.6814C>T (p.Arg2272Cys) | Het | 0.000016 | VUS (PM2, PP2) |
|  |  |  | NM_018117.11(*WDR11*):c.3571G>A (p.Gly1191Ser) | Het | 0.000141 | VUS (PM2, PP3) |
| 5153 | F |  | NM_178822.4(*IGSF10*):c.187A>C (p.Asn63His) | Het | - | VUS (PM2) |
|  |  |  | NM_005045.3(*RELN*):c.1108G>C (p.Gly370Arg) | Het | 0.000255 | VUS (PM2, PP2, BP6) |
| 5155 | F |  | NM_032242.3(*PLXNA1*):c.3110A>G (p.Asn1037Ser) | Het | 0.000064 | VUS (PM2, PP2, BP4) |
|  |  |  | NM_000264.3(*PTCH1*):c.3487G>A (p.Gly1163Ser) | Het | 0.000513 | VUS (PM5, PP2, PP3, BP6) |
| 5156 | M |  | NM_012431.2(*SEMA3E*):c.142A>G (p.Ile48Val) | Het | - | VUS (PM2, BP4) |
| 5157 | M |  | NM_001382323.2(*PKNOX2*):c.846C>A (p.Asp282Glu) | Het | - | VUS (PM2) |
|  |  |  | NM_018344.5(*SLC29A3*):c.707C>T (p.Thr236Met) | Het | 0.000350 | VUS |
| 5158 | F |  | NM_003612.3(*SEMA7A*):c.1864C>T (p.Arg622Cys) | Het | 0.000598 | VUS (PM2, BP4) |
| 5160 | M |  | NM_000479.3(*AMH*):c.1054C>T (p.Pro352Ser) | Het | 0.000476 | VUS |
|  |  |  | NM_015662.2(*IFT172*):c.1243G>A (p.Gly415Arg) | Het | 0.000024 | VUS (PM2) |
|  |  |  | NM_003873.7(*NRP1*):c.1030G>A (p.Ala344Thr) | Het | 0.000020 | VUS (PM2, PP3) |
|  |  |  | NM_004959.4(*NR5A1*):c.523G>A (p.Gly175Ser) | Het | 0.000009 | VUS (PM2, PP2) |
|  |  |  | NM_032242.3(*PLXNA1*):c.1832A>G (p.Glu611Gly) | Het | 0.000012 | VUS (PM2, PP2, BP4) |
|  |  |  | NM_018082.5(*POLR3B*):c.469A>G (p.Lys157Glu) | Het | 0.000008 | VUS (PM2, PP2) |
|  |  |  | NM_001059.2(*TACR3*):c.496A>C (p.Ile166Leu) | Het | 0.000007 | VUS (PM2) |
| 5463 | F |  | NM_031226.2(*CYP19A1*):c.1094G>A (p.Arg365Gln) | Het | 0.000032 | P (PS4, PP3, PM2) |
| 5469 | F |  | NM_015295.2(*SMCHD1*):c.5737C>G (p.Arg1913Gly) | Het | - | VUS (PM2, PP2, BP4) |
| 5470 | M |  | NM_004959.4(*NR5A1*):c.787G>A (p.Gly263Ser) | Het | 0.000041 | VUS (PM1, PM2, PP2) |
|  |  |  | NM_022370.3(*ROBO3*):c.377C>A (p.Pro126Gln) | Het | 0.000046 | VUS (PM2) |
| 5471 | M |  | NM_178822.4(*IGSF10*):c.2210_2211delTT (p.Phe737*) | Het | 0.000736 | VUS (PM2, BP6) |
|  |  |  | NM_000264.3(*PTCH1*):c.1628G>A (p.Arg543His) | Het | 0.000016 | VUS (PM2, PP2, PP3, BP6) |
| 5473 | M |  | NM_175737.3(*KLB*):c.2329_2331delTTC (p.Phe777del) | Het | 0.002451 | VUS (PM2, BS2, PM4) |
| 5477 | F |  | None |  |  |  |
| 5480 | M |  | NM_003612.3(*SEMA7A*):c.379G>A (p.Glu127Lys) | Het | 0.000053 | VUS (PM2, BP4) |
| 5485 | F |  | NM_178822.4(*IGSF10*):c.1420A>G (p.Met474Val) | Het | 0.000028 | VUS (PM2, BP4) |
|  |  |  | NM_018848.3(*MKKS*):c.1318C>G (p.Gln440Glu) | Het | 0.000064 | VUS (PM2) |
| 5491 | M |  | None |  |  |  |
| 5496 | M |  | NM_004787.3(*SLIT2*):c.1850G>A (p.Arg617Gln) | Het | 0.000080 | VUS (PM2, PP2, BP4) |
| 5497 | M |  | NM_000479.3(*AMH*):c.16C>G (p.Leu6Val) | Het | 0.000005 | VUS (PM2, BP4) |
| 5540 |  |  | NM_207359.3(*GADL1*):c.8G>A (p.Ser3Asn) | Het | 0.000071 | VUS (PM2, BP4) |
|  | F |  | NM_002256.3(*KISS1*):c.244C>A (p.Gln82Lys) | Het | 0.000161 | VUS (PM2, BP4) |
| 5544 | F |  | NM_000439.4(*PCSK1*):c.680T>C (p.Val227Ala) | Het | - | VUS (PM2, PP3) |
| 5546 | M |  | NM_005076.3(*CNTN2*):c.2050C>T (p.Arg684Trp) | Het | 0.000004 | VUS (PM2, PP2) |
|  |  |  | NM_004439.5(*EPHA5*):c.1480C>T (p.Arg494Cys) | Het | 0.000199 | VUS (PM2) |
| 5547 | M |  | NM_000601.4(*HGF*):c.1787C>T (p.Thr596Met) | Het | 0.00006 | VUS (PM2) |
|  |  |  | NM_005045.3(*RELN*):c.6770C>T (p.Ser2257Leu) | Het | 0.00004 | VUS (PP2, PM2) |
| 5554 | F |  | NM_017563.4(*IL17RD*):c.665C>T (p.Pro222Leu) | Het | 0.000028 | VUS (PM2) |
|  |  |  | NM_022370.3(*ROBO3*):c.323G>T (p.Arg108Leu) | Het | 0.000008 | VUS (PM2) |
| 5555 | M |  | NM_005076.3(*CNTN2*):c.689C>T (p.Ala230Val) | Het | 0.000040 | VUS (PM2, PP2, BP4) |
|  |  |  | NM_018117.11(*WDR11*):c.2305A>G (p.Met769Val) | Het | 0.000562 | VUS (BS1) |
| 5557 | M |  | NM_005228.3(*EGFR*):c.635A>G (p.Lys212Arg) | Het | - | VUS (PM2, BP4) |
|  |  |  | NM_004439.5(*EPHA5*):c.1480C>T (p.Arg494Cys) | Het | 0.000199 | VUS (PM2) |
|  |  |  | NM_004807.2(*HS6ST1*):c.917G>A (p.Arg306Gln) | Het | 0.000694 | VUS (PM2, PM5, PP3) |
| 5559 | F |  | NM_178822.4(*IGSF10*):c.5278G>A (p.Gly1760Arg) | Het | 0.000032 | VUS (PM2, PP3) |
|  |  |  | NM_207111.4(*RNF216*):c.1865C>T (p.Thr622Met) | Het | 0.000014 | VUS (PM2, BP4) |
| 5570 | M |  | None |  |  |  |
| 5575 | F |  | NM_178822.4(*IGSF10*):c.3941C>T (p.Thr1314Met) | Het | 0.000138 | VUS (PM2, BP4) |
| 5579 | M |  | NM_014875.2(*KIF14*):c.1009G>A (p.Glu337Lys) | Het | 0.000134 | VUS (PM2, BP4) |
|  |  |  | NM_005912.2(*MC4R*):c.94G>A (p.Gly32Arg) | Het | - | VUS (PM2, PP2, BP4) |
|  |  |  | NM_017514.4(*PLXNA3*):c.85A>G (p.Thr29Ala) | Hemi | 0.000006 | VUS (PM2, BP4) |
|  |  |  | NM_198309.3(*TTC8*):c.1219C>T (p.His407Tyr) | Het | - | VUS (PM2) |
| 5725 | M |  | NM_000479.3(*AMH*):c.1505G>T (p.Arg502Leu) | Het | 0.000034 | VUS (PM2) |
| 5793 | M |  | NM_033343.3(*LHX4*):c.256G>A (p.Gly86Ser) | Het | 0.000021 | VUS (PM2, PP3) |
|  |  |  | NM_018082.5(*POLR3B*):c.1244T>C (p.Met415Thr) | Het | 0.000566 | LP (PS4, PP2, PP3, BS1) |
| 6036 | F |  | NM_002309.3(*LIF*):c.481G>A (p.Val161Met) | Het | 0.000014 | VUS (PM2) |
| 6040 | F |  | NM_032242.3(*PLXNA1*):c.2690G>A (p.Arg897His) | Het | 0.000975 | VUS (PM2, PP2, BP4) |
|  |  |  | NM_017514.4(*PLXNA3*):c.4861G>A (p.Asp1621Asn) | Het | 0.000016 | VUS (PM2, BS2) |
|  |  |  | NM_005861.4(*STUB1*):c.44C>T (p.Ala15Val) | Het | 0.000078 | VUS (PM2, PP2, BP4) |
| 6045 | F |  | NM_005076.3(*CNTN2*):c.191G>A (p.Arg64Gln) | Het | 0.000036 | VUS (PM2, PP2) |
|  |  |  | NM_015100.4(*POGZ*):c.3979A>G (p.Ser1327Gly) | Het | 0.000018 | VUS (PM2, PP2, BP6) |
| 6048 | M |  | NM_000168.5(*GLI3*):c.2395C>A (p.Pro799Thr) | Het | - | VUS (PM2, BP4) |
| 6051 | M |  | NM_174975.5(*SEC14L3*):c.1120G>A (p.Ala374Thr) | Het | 0.000272 | VUS (PM2, BP4) |
|  |  |  | NM_018344.5(*SLC29A3*):c.1285C>A (p.Leu429Ile) | Het | 0.000011 | VUS (PM2) |
| 6052 | M |  | NM_017780.3(*CHD7*):c.4972G>C (p.Glu1658Gln) | Het | - | VUS (PM2, PP2) |
|  |  |  | NM_017780.3(*CHD7*):c.5533G>A (p.Gly1845Arg) | Het | 0.00004 | VUS (PM2, PP2, PP3) |
|  |  |  | NM_005045.3(*RELN*):c.5822T>C (p.Val1941Ala) | Het | 0.000358 | VUS (PP2, BP4) |
|  |  |  | NM_006378.3(*SEMA4D*):c.1528G>A (p.Glu510Lys) | Het | 0.000004 | VUS (PM2, BP4) |
|  |  |  | NM_198309.3(*TTC8*):c.1219C>T (p.His407Tyr) | Het | - | VUS (PM2) |
| 6055 | M |  | NM_000163.4(*GHR*):c.1156C>T (p.Arg386Cys) | Het | 0.000336 | VUS |
|  |  |  | NM_014564.4(*LHX3*):c.748G>A (p.Val250Ile) | Het | - | VUS (PM2) |
|  |  |  | NM_001037293.3(*PALM2*):c.464C>G (p.Ser155Cys) | Het | 0.000004 | VUS (PM2) |
| 6056 | F |  | NM_000163.4(*GHR*):c.1156C>T (p.Arg386Cys) | Het | 0.000336 | VUS (BS4) |
| 6058 | M |  | None |  |  |  |
| 6077 | F |  | NM_004491.5(*ARHGAP35*):c.1190T>A (p.Met397Lys) | Het | - | VUS (PM2, PP2, BP4) |
|  |  |  | NM_015681.3(*B9D1*):c.278A>G (p.Asp93Gly) | Het | - | VUS (PM2, PP3) |
|  |  |  | NM_002303.5(*LEPR*):c.1246C>T (p.His416Tyr) | Het | 0.000295 | VUS |
|  |  |  | NM_000620.4(*NOS1*):c.142C>T (p.Arg48Cys) | Het | 0.000064 | VUS (PM2, PP2) |
|  |  |  | NM_000620.4(*NOS1*):c.234C>G (p.Asp78Glu) | Het | - | VUS (PM2, PP2, BP4) |
| 6086 | F |  | NM_004958.3(*MTOR*):c.4885A>C (p.Ile1629Leu) | Het | 0.000004 | VUS (PM2, PP2) |
|  |  |  | NM_183059.2(*RD3*):c.202C>T (p.Arg68Trp) | Het | 0.000265 | VUS (BP4) |
| 6091 | F |  | None |  |  |  |
| 6119 | F |  | None |  |  |  |
| 6392 | M |  | NM_006080.2(*SEMA3A*):c.271A>G (p.Ile91Val) | Het | 0.000020 | VUS (PM2) |
|  |  |  | NM_018344.5(*SLC29A3*):c.40A>G (p.Asn14Asp) | Het | 0.000036 | VUS (PM2, BP4) |
| 6472 | F |  | None |  |  |  |
| 6545 | F |  | NM_015295.2(*SMCHD1*):c.23G>A (p.Gly8Glu) | Het | - | VUS (PM2, PP2, BP4) |
| 6559 | F |  | NM_000921.4(*PDE3A*):c.1219T>G (p.Cys407Gly) | Het | - | VUS (PM2) |
|  |  |  | NM_018117.11(*WDR11*):c.3571G>A (p.Gly1191Ser) | Het | 0.000141 | VUS (PM2, PP3) |
| 6667 | F |  | NM_001143821.3(*PLEKHA5*):c.2200G>C (p.Glu734Gln) | Het | 0.000217 | VUS (PM2) |
|  |  |  | NM_007055.3(*POLR3A*):c.3388G>C (p.Val1130Leu) | Het | - | VUS (PM2, PP2) |
| 6905 | M |  | NM_002673.4(*PLXNB1*):c.655G>A (p.Val219Met) | Het | 0.001526 | VUS (PM2, BS2, PP2) |
| 6906 | F |  | NM_000168.5(*GLI3*):c.2887G>A (p.Ala963Thr) | Het | 0.000024 | VUS (PM2, BP4) |
|  |  |  | NM_007252.4(*POU6F2*):c.552_553insAG (p.Gln185Serfs*118) | Het | 0.000703 | LP (PVS1, PM2) |
| 6907 | M |  | NM_006702.4(*PNPLA6*):c.3632G>A (p.Arg1211His) | Het | 0.000016 | VUS (PM2, PP2) |
| 6908 | M |  | NM_001080414.4(*CCDC88C*):c.3596A>G (p.His1199Arg) | Het | 0.000095 | VUS (PM2) |
|  |  |  | NM_001946.2(*DUSP6*):c.11C>G (p.Thr4Arg) | Het | 0.000022 | VUS (PM2, BP4) |
| 7029 | F |  | NM_024574.3(*NDNF*):c.239C>T (p.Thr80Met) | Het | 0.001357 | VUS |
| 7111 | M |  | NM_016952.4(*CDON*):c.2177G>T (p.Arg726Leu) | Het | - | VUS (PM2) |
|  |  |  | NM_006614.2(*CHL1*):c.2488G>A (p.Val830Met) | Het | 0.000024 | VUS (PM2, BP4) |
|  |  |  | NM_006702.4(*PNPLA6*):c.3067C>T (p.His1023Tyr) | Het | 0.000081 | VUS (PM2, PP2) |
|  |  |  | NM_000264.3(*PTCH1)*:c.2689A>G (p.Ile897Val) | Het | 0.000025 | VUS (PP2, PM2, BP6) |
| 7116 | F |  | NM_024685.4(*BBS10*):c.765G>A (p.Met255Ile) | Het | 0.000595 | VUS (PM1, PP2, PM2, BP6) |
|  |  |  | NM_015662.2(*IFT172*):c.1513C>T (p.Arg505Trp) | Het | 0.000074 | VUS (BS2) |
|  |  |  | NM_014564.4(*LHX3*):c.949G>A (p.Gly317Ser) | Het | 0.000014 | VUS (PM2, BP4) |
| 7329 | M |  | NM_004958.3(*MTOR*):c.1688A>G (p.His563Arg) | Het | 0.000004 | VUS (PM2, PP2) |
| 7630 | M |  | NM_015910.7(*WDPCP*):c.1606C>G (p.Leu536Val) | Het | 0.000008 | VUS (PM2) |
| 7631 | F |  | NM_020116.5(*FSTL5*):c.1358T>C (p.Met453Thr) | Het | 0.001177 | VUS (PM2) |
|  |  |  | NM_004787.3(*SLIT2*):c.3724A>G (p.Ile1242Val) | Het | 0.000025 | VUS (PM2, PP2, BP4) |
| 7632 | F |  | NM_005270.4(*GLI2*):c.8C>T (p.Thr3Met) | Het | 0.000032 | VUS (PM2, BP4) |
| 7633 | F |  | NM_213653.3(*HJV*):c.46A>C (p.Ser16Arg) | Het | - | VUS (PM2, PP2) |
| 7634 | M |  | NM_000479.3(*AMH*):c.790G>C (p.Gly264Arg) | Het | 0.000129 | VUS (PP3, BS1) |
|  |  |  | NM_174975.5(*SEC14L3*):c.81delT (p.Pro28Leufs*52) | Het | 0.000219 | VUS (PM2) |
| 7635 | M |  | NM_000479.3(*AMH*):c.1668G>C (p.Glu556Asp) | Het | - | VUS (PM2) |
|  |  |  | NM_000620.4(*NOS1*):c.1517T>C (p.Phe506Ser) | Het | - | VUS (PM2, PP2) |
|  |  |  | NM_004787.3(*SLIT2*):c.688G>T (p.Val230Phe) | Het | 0.000040 | VUS (PM2, PP2) |
| 7636 | F |  | NM_005270.4(*GLI2*):c.592G>A (p.Gly198Arg) | Het | 0.000050 | VUS (PM2) |
| 7637 | F |  | NM_000479.3(*AMH)*:c.790G>C (p.Gly264Arg) | Het | 0.000129 | VUS (PP3, BS1) |
|  |  |  | NM_017780.3(*CHD7*):c.2209_2211delCCT (p.Pro737del) | Het | 0.000086 | VUS (PM4, BP6) |
|  |  |  | NM_019066.5(*MAGEL2*):c.263C>T (p.Pro88Leu) | Het | 0.000202 | VUS (PM2, BP6) |
|  |  |  | NM_000439.4(*PCSK1*):c.170T>C (p.Leu57Pro) | Het | - | VUS (PM2) |
|  |  |  | NM_006378.3(*SEMA4D*):c.1957G>A (p.Val653Ile) | Het | 0.000028 | VUS (PM2, BP4) |
|  |  |  | NM_018117.11(*WDR11*):c.2584C>G (p.Gln862Glu) | Het | - | VUS (PM2, PP3) |
| 7641 | F |  | NM_002673.4(*PLXNB1*):c.378G>T (p.Gln126His) | Het | - | VUS (PM2, PP2, BP4) |
|  |  |  | NM_007055.3(*POLR3A*):c.3840G>A (p.Met1280Ile) | Het | 0.000014 | VUS (PM2, PP2) |
|  |  |  | NM_005045.3(*RELN*):c.5467G>A (p.Ala1823Thr) | Homo | - | VUS (PM2, PP2) |
| 7642 | M |  | None |  |  |  |
| 7644 | M |  | NM_000479.3(*AMH*):c.1232G>A (p.Cys411Tyr) | Het | - | VUS (PM2) |
|  |  |  | NM_004004.6(*GJB2*):c.101T>C (p.Met34Thr) | Het | 0.008996 | LP (PM1, PM2, PM5, PP2, PP3, PP5) |
|  |  |  | NM_213653.3(*HJV*):c.1246T>C (p.Ser416Pro) | Het | 0.000057 | VUS (PP2) |
|  |  |  | NM_030964.5(*SPRY4*):c.722C>A (p.Ser241Tyr) | Het | 0.004530 | VUS (PM2, BP6) |
| 7648 | M |  | NM_006261.4(*PROP1*):c.301_302delAG (p.Leu102Cysfs*8) | Het | 0.000181 | P (PVS1, PS3, PM2, PM3, PP1) |
| 7656 | M |  | NM_016952.4(*CDON*):c.2383G>A (p.Val795Ile) | Het | 0.000074 | VUS (PM2, BP6) |
|  |  |  | NM_004004.6(*GJB2)*:c.663G>C (p.Lys221Asn) | Het | 0.000043 | VUS (PM2, PP2) |
| 7663 | M |  | NM_014564.4(*LHX3*):c.400G>A (p.Asp134Asn) | Het | 0.000050 | VUS (PM2, PP3) |
| 7667 | F |  | None |  |  |  |
| 7674 | F |  | NM_001030055.2(*ARHGAP5*):c.3290C>A (p.Thr1097Lys) | Het | 0.000096 | VUS (PM2, PP2) |
|  |  |  | NM_017617.4(*NOTCH1*):c.6644C>T (p.Ser2215Leu) | Het | - | VUS (PM2, PP2, PP3) |
|  |  |  | NM_006702.4(*PNPLA6*):c.3598C>G (p.Gln1200Glu) | Het | 0.000166 | VUS (PM2, PP2) |
| 7675 | M |  | NM_005215.3(*DCC*):c.4027C>T (p.Arg1343Cys) | Het | 0.000141 | VUS (PM2) |
|  |  |  | NM_017563.4(*IL17RD*):c.1900C>T (p.Arg634Trp) | Het | 0.000012 | VUS (PM2, BP4) |
|  |  |  | NM_002673.4(*PLXNB1*):c.655G>A (p.Val219Met) | Het | 0.001526 | VUS (PM2, BS2, PP2) |
| 7676 | M |  | NM_001080414.4(*CCDC88C*):c.1885C>T (p.Arg629Trp) | Het | 0.000075 | VUS (PM2) |
|  |  |  | NM_021620.4(*PRDM13*):c.934T>C (p.Tyr312His) | Het | 0.000261 | VUS (PM2, BP4) |
|  |  |  | NM_207036.1(*TCF12*):c.1409G>A (p.Ser470Asn) | Het | 0.000011 | VUS (PM2, BP4) |
| 7678 | F |  | NM_015681.3(*B9D1*):c.380C>T (p.Thr127Met) | Het | 0.000028 | VUS (PM2) |
| 7680 | F |  | NM_001174116.3(*DMXL2*):c.4670C>T (p.Ser1557Leu) | Het | 0.000016 | VUS (PM2, PP2) |
|  |  |  | NM_178822.4(*IGSF10*):c.2204G>A (p.Arg735Gln) | Het | 0.000050 | VUS (PM2, BP4) |
| 7681 | M |  | None |  |  |  |
| 7682 | F |  | NM_000168.5(*GLI3*):c.1471T>C (p.Phe491Leu) | Het | 0.000018 | VUS (PM2, PP3, BP6) |
|  |  |  | NM_178822.4(*IGSF10*):c.6006T>G (p.Phe2002Leu) | Het | 0.000280 | VUS (PM2, BP4) |
|  |  |  | NM_017617.4(*NOTCH1*):c.1543G>C (p.Glu515Gln) | Het | 0.000061 | VUS (PM2, PP2) |
|  |  |  | NM_001059.2(*TACR3*):c.918G>A (p.Met306Ile) | Het | 0.000128 | VUS (PM2, BP4) |
| 7684 | F |  | NM_173648.4(*CCDC141*):c.103-1G>T | Het | 0.000018 | VUS (PM2) |
|  |  |  | NM_017563.4(*ILR17D*):c.964C>T (p.Arg322Cys) | Het | 0.000049 | VUS (PM2) |
|  |  |  | NM_004822.2(*NTN1*):c.34_42delCTGGCGGCG (p.Leu12_Ala14del) | Het | 0.000090 | VUS (PM2, PM4) |
|  |  |  | NM_000264.3(*PTCH1*):c.67G>A (p.Ala23Thr) | Het | - | VUS (PM2, PP2) |
| 7685 | F |  | NM_005045.3(*RELN*):c.5345G>A (p.Arg1782His) | Het | 0.000011 | VUS (PM2, PP2, BP4) |
| 7686 | M |  | NM_022659.4(*EBF2*):c.1513G>A (p.Gly505Ser) | Het | 0.000071 | VUS (PM2, BP4) |
|  |  |  | NM_178822.4(*IGSF10*):c.7334_7336dupTCA (p.Ile2445dup) | Het | 0.000173 | VUS (PM2, PM4) |
| 7687 | F |  | NM_000388.3(*CASR*):c.2563A>C (p.Asn855His) | Het | - | VUS (PM2, PP2) |
|  |  |  | NM_000515.3(*GH1*):c.134G>A (p.Arg45His) | Het | 0.000187 | VUS (BS2) |
| 7688 | F |  | NM_000439.4(*PCSK1*):c.1654G>T (p.Asp552Tyr) | Het | - | VUS (PM2, PP3) |
| 7689 | F |  | NM_001273.3(*CHD4*):c.304C>T (p.Arg102Cys) | Het | 0.000039 | VUS (PM2, PP2, PP3) |
|  |  |  | NM_019066.5(*MAGEL2*):c.3464A>C (p.Lys1155Thr) | Het | 0.000032 | VUS (PM2) |
|  |  |  | NM_015537.4(*NSMF*):c.1091G>A (p.Arg364His) | Het | 0.000004 | VUS (PM2) |
|  |  |  | NM_144773.3(*PROKR2*):c.1044G>A (p.Met348Ile) | Het | - | VUS (PM2, BP4) |
| 7694 | F |  | NM_000163.4(*GHR*):c.679G>C (p.Glu227Gln) | Het | - | VUS (PM2, PP3) |
| 7703 | M |  | NM_000620.4(*NOS1*):c.1082C>T (p.Pro361Leu) | Het | 0.000056 | VUS (PM2, PP2, BP4) |
|  |  |  | NM_012233.3(*RAB3GAP1*):c.244G>A (p.Glu82Lys) | Het | 0.000004 | VUS (PM2, BP4) |
| 7712 | M |  | NM_004439.5(*EPHA5*):c.1480C>T (p.Arg494Cys) | Het | 0.000199 | VUS (PM2) |
|  |  |  | NM_032551.4(*KISS1R*):c.305T>C (p.Leu102Pro) | Het | 0.000027 | LP (PS4, PS3, PM2) |
| 7715 | F |  | NM_000076.2(*CDKN1C*):c.355C>T (p.Leu119Phe) | Het | 0.000011 | VUS (PM2) |
|  |  |  | NM_017514.4(*PLXNA3*):c.719C>T (p.Thr240Met) | Het | 0.000127 | VUS (PM2, BS2) |
|  |  |  | NM_207111.4(*RNF216*):c.230A>G (p.Asn77Ser) | Het | 0.000089 | VUS (PM2, BP4) |
| 7722 | F |  | NM_000479.3(*AMH*):c.1505G>T (p.Arg502Leu) | Het | 0.000034 | VUS (PM2) |
|  |  |  | NM_003873.7(*NRP1*):c.1610C>T (p.Ala537Val) | Het | 0.000014 | VUS (PM2) |
| 7728 | F |  | NM_004439.5(*EPHA5*):c.2726A>G (p.Gln909Arg) | Het | - | VUS (PM2) |
|  |  |  | NM_002673.4(*PLNB1*):c.479T>G (p.Leu160Trp) | Het | - | VUS (PM2, PP2, BP4) |
| 7729 | F |  | NM_007055.3(*POLR3A*):c.1177C>T (p.Pro393Ser) | Het | 0.000004 | VUS (PM2, PP2, PP3) |
| 7736 | F |  | NM_004004.6(*GJB2*):c.101T>C (p.Met34Thr) | Het | 0.008996 | LP (PM1, PM2, PM5, PP2, PP3, PP5) |
|  |  |  | NM_012414.3(*RAB3GAP2*):c.1657G>A (p.Asp553Asn) | Het | 0.000106 | VUS (PM2) |
|  |  |  | NM_031466.5(*TRAPPC9*):c.2671A>C (p.Thr891Pro) | Het | 0.000191 | VUS (BS1) |
| 7742 | M |  | NM_020116.5(*FSTL5*):c.447A>T (p.Lys149Asn) | Het | 0.000260 | VUS (PM2) |
| 7743 | M |  | NM_018344.5(*SLC29A3*):c.269C>T (p.Thr90Ile) | Het | 0.000085 | VUS (PM2, BP4) |
|  |  |  | NM_207036.1(*TCF12*):c.454C>T (p.Pro152Ser) | Het | 0.000329 | VUS (BP4) |
| 7744 | F |  | NM_000730.3(*CCKAR*):c.685T>C (p.Tyr229His) | Het | 0.000025 | VUS (PM2, PP3) |
| 7745 | F |  | NM_016952.4(*CDON*):c.1495A>G (p.Ile499Val) | Het | 0.000004 | VUS (PM2, BP4) |
|  |  |  | NM_003467.2(*CXCR4*):c.50G>A (p.Gly17Asp) | Het | 0.000008 | VUS (PM2, BP4) |
|  |  |  | NM_032844.5(*MASTL*):c.496A>T (p.Asn166Tyr) | Het | 0.000032 | VUS (PM2, PP3) |
|  |  |  | NM_001143821.3(*PLEKHA5*):c.2530A>G (p.Met844Val) | Het | 0.000100 | VUS (PM2, BP4) |
|  |  |  | NM_006293.3(*TYRO3*):c.2264C>T (p.Pro755Leu) | Het | 0.000057 | VUS (PM2) |
| 7763 | F |  | NM_173648.4(*CCDC141*):c.3321delA (p.Leu1107Phefs*12) | Het | 0.000292 | VUS (PM2) |
|  |  |  | NM_004822.2(*NTN1*):c.1693C>G (p.Pro565Ala) | Het | - | VUS (PM2) |
| 7764 | F |  | NM_178822.4(*IGSF10*):c.2359C>T (p.Pro787Ser) | Het | - | VUS (PM2, BP4) |
|  |  |  | NM_017563.4(*IL17RD*):c.392A>C (p.Lys131Thr) | Het | 0.000856 | VUS (PM2) |
|  |  |  | NM_018117.11(*WDR11*):c.1449G>C (p.Met483Ile) | Het | 0.000305 | VUS |
| 7769 | M |  | NM_173648.4(*CCDC141*):c.1394G>C (p.Gly465Ala) | Het | 0.000188 | VUS (PM2, BP4) |
|  |  |  | NM_173648.4(*CCDC141*):c.1396delT (p.Tyr466Thrfs*33) | Het | 0.000188 | VUS (PM2) |
|  |  |  | NM_012414.3(*RAB3GAP2*):c.1580C>T (p.Pro527Leu) | Het | 0.000308 | VUS (PP3, BS1) |
| 7770 | M |  | NM_173648.4(*CCDC141*):c.4565C>T (p.Ser1522Phe) | Het | 0.000029 | VUS (PM2, BP4) |
|  |  |  | NM_018082.5(*POLR3B*):c.1502A>G (p.His501Arg) | Het | - | VUS (PM2, PP2, PP3) |
| 7778 | M |  | NM_001080414.4(*CCDC88C*):c.3628G>A (p.Gly1210Arg) | Het | 0.000049 | VUS (PM2, BP4) |
|  |  |  | NM_015537.4(*NSMF*):c.1019G>C (p.Gly340Ala) | Het | 0.000011 | VUS (PM2, BP4) |
|  |  |  | NM_004787.3(*SLIT2*):c.287G>A (p.Arg96Lys) | Het | 0.000020 | VUS (PM2, PP2, BP4) |
| 7789 | M |  | NM_017617.4(*NOTCH1*):c.7397C>G (p.Thr2466Arg) | Het | - | VUS (PM2, PP2) |
| 7791 | M |  | NM_000730.3(*CCKAR*):c.329G>A (p.Gly110Glu) | Het | 0.000011 | VUS (PM2, PP3) |
|  |  |  | NM_000168.5(*GLI3*):c.245G>A (p.Arg82Lys) | Het | 0.000014 | VUS (PM2, BP6) |
|  |  |  | NM_004822.2(*NTN1*):c.1466A>G (p.Lys489Arg) | Het | 0.000145 | VUS (PM2) |
| 7792 | M |  | NM_177976.1(*ARL6*):c.538C>T (p.Gln180*) | Het | 0.000008 | VUS (PM2, PVS1) |
|  |  |  | NM_002673.4(*PLXNB1*):c.2653C>A (p.Leu885Ile) | Het | 0.000004 | VUS (PM2, PP2, BP4) |
|  |  |  | NM_005045.3(*RELN*):c.7C>G (p.Arg3Gly) | Het | - | VUS (PM2, PP2, BP4) |
| 7793 | F |  | NM_201266.1(*NRP2*):c.1198G>A (p.Ala400Thr) | Het | 0.000088 | VUS (PM2) |
| 7804 | M |  | NM_015537.4(*NSMF*):c.1453G>A (p.Val485Ile) | Het | 0.000039 | VUS (PM2, BP4) |
|  |  |  | NM_000921.4(*PDE3A*):c.761A>G (p.Tyr254Cys) | Het | 0.000591 | VUS (PM2) |
| 7805 | F |  | NM_000620.4(*NOS1*):c.3092A>T (p.Gln1031Leu) | Het | 0.000004 | VUS (PM2, PP2) |
| 7806 | M |  | NM_213653.3(*HJV*):c.1003C>T (p.Arg335Trp) | Het | 0.000011 | VUS (PM2, PP2) |
|  |  |  | NM_015662.2(*IFT172*):c.4147G>A (p.Glu1383Lys) | Het | 0.000213 | VUS |
|  |  |  | NM_019066.5(*MAGEL2*):c.2028G>T (p.Glu676Asp) | Het | 0.000016 | VUS (PM2) |
|  |  |  | NM_024574.3(*NDNF*):c.239C>T (p.Thr80Met) | Het | 0.001357 | VUS |
|  |  |  | NM_012414.3(*RAB3GAP2*):c.3355G>A (p.Glu1119Lys) | Het | - | VUS (PM2, BP4) |
| 7807 | F |  | None |  |  |  |
| 7813 | F |  | NM_005215.3(*DCC*):c.3404C>T (p.Thr1135Ile) | Het | 0.000012 | VUS (PM2, BP4) |
|  |  |  | NM_032844.5(*MASTL*):c.1012A>G (p.Met338Val) | Het | 0.000007 | VUS (PM2, BP4) |
| 7814 | F |  | NM_001024613.3(*FEZF1*):c.727T>C (p.Phe243Leu) | Het | - | VUS (PM2) |
|  |  |  | NM_178822.4(*IGSF10*):c.620A>G (p.Tyr207Cys) | Het | 0.000110 | VUS (PM2) |
| 7820 | F |  | NM_001080414.4(*CCDC88C*):c.5332T>C (p.Ser1778Pro) | Het | 0.000012 | VUS (PM2, BP4) |
|  |  |  | NM_001127500.1(*MET*):c.305G>A (p.Ser102Asn) | Het | 0.000008 | VUS (PM2, BP4) |
|  |  |  | NM_201266.1(*NRP2*):c.292G>T (p.Ala98Ser) | Het | 0.000012 | VUS (PM2) |
|  |  |  | NM_032242.3(*PLXNA1*):c.2908C>T (p.Arg970Cys) | Het | 0.000004 | VUS (PM2, PP2) |
|  |  |  | NM_017514.4(*PLXNA3*):c.1249G>A (p.Val417Met) | Het | 0.000162 | VUS (PM2, BS2) |
|  |  |  | NM_002673.4(*PLXNB1*):c.2986C>T (p.Arg996Cys) | Het | 0.000141 | VUS (PM2, PP2, BP4) |
| 7821 | M |  | NM_033163.3(*FGF8*):c.652C>A (p.Leu218Met) | Het | - | VUS (PM2, PP2) |
|  |  |  | NM_020116.5(*FSTL5*):c.871A>T (p.Asn291Tyr) | Het | 0.002126 | VUS (PM2, BS2) |
|  |  |  | NM_178822.4(*IGS10*):c.823G>A (p.Ala275Thr) | Het | 0.000131 | VUS (PM2, BP4) |
|  |  |  | NM_017563.4(*IL17RD*):c.1900C>T (p.Arg634Trp) | Het | 0.000012 | VUS (PM2, BP4) |
|  |  |  | NM_001102653.1(*OTUD4*):c.1042C>T (p.Arg348Trp) | Het | 0.000036 | VUS (PM2) |
|  |  |  | NM_031466.5(*TRAPPC9*):c.140G>A (p.Arg47Gln) | Het | 0.000315 | VUS (PM2, BP4) |
| 7826 | M |  | None |  |  |  |
| 7827 | M |  | NM_004004.6(*GJB2*):c.101T>C (p.Met34Thr) | Het | 0.008996 | LP (PM1, PM2, PM5, PP2, PP3, PP5) |
| 7828 | M |  | NM_178822.4(*IGSF10*):c.4187C>T (p.Ser1396Phe) | Het | 0.000233 | VUS (PM2, BP4) |
| 7833 | M |  | NM_015662.2(*IFT172*):c.4363C>T (p.Arg1455Trp) | Het | 0.000322 | VUS |
| 7835 | F |  | NM_023110.2(*FGFR1*):c.2464C>T (p.Arg822Cys) | Het | 0.000256 | VUS (PP2, BP6) |
|  |  |  | NM_015662.2(*IFT172*):c.1426G>A (p.Gly476Ser) | Het | 0.000209 | VUS (PM2) |
|  |  |  | NM_017563.4(*IL17RD*):c.359C>T (p.Ser120Leu) | Het | 0.000021 | VUS (PM2) |
|  |  |  | NM_004787.3(*SLIT2*):c.4400G>T (p.Gly1467Val) | Het | 0.000266 | VUS (PM2, PP2) |
| 7836 | F |  | NM_033163.3(*FGF8*):c.693C>A (p.Ser231Arg) | Het | - | VUS (PM2, PP2, BP4) |
|  |  |  | NM_000510.2(*FSHB*):c.327C>A (p.Ser109Arg) | Het | 0.000769 | VUS (PM2, BS2) |
|  |  |  | NM_002673.4(*PLXNB1*):c.2450A>G (p.Asp817Gly) | Het | 0.000111 | VUS (PM2, PP2, BP4) |
| 7837 | M |  | NM_019066.5(*MAGEL2*):c.1720C>G (p.Leu574Val) | Het | 0.000091 | VUS (BP6) |
| 7839 | M |  | NM_021913.3(*AXL*):c.2399G>A (p.Arg800Gln) | Het | 0.000085 | VUS (PM2) |
|  |  |  | NM_032242.3(*PLXNA1*):c.1114C>T (p.Arg372Cys) | Het | 0.000008 | VUS (PM2, PP2) |
|  |  |  | NM_015910.7(*WDPCP*):c.1606C>G (p.Leu536Val) | Het | 0.000008 | VUS (PM2) |
| 7840 | M |  | NM_000388.3(*CASR*):c.2563A>C (p.Asn855His) | Het | - | VUS (PM2, PP2) |
|  |  |  | NM_018117.11(*WDR11*):c.3571G>A (p.Gly1191Ser) | Het | 0.000141 | VUS (PM2, PP3) |
| 7844 | M |  | NM_014875.2(*KIF14*):c.3181A>G (p.Ile1061Val) | Het | 0.000078 | VUS (PM2, BP4) |
| 7845 | F |  | NM_000406.2(*GNRHR*):c.317A>G (p.Gln106Arg) | Het | 0.002749 | P (PM1, PP2, PM2, PP5) |
|  |  |  | NM_178822.4(*IGSF10*):c.2210_2211delTT (p.Phe737*) | Het | 0.000736 | VUS (PM2, BP6) |
| 7850 | M |  | NM_005235.2(*ERBB4*):c.890T>G (p.Phe297Cys) | Het | - | VUS (PM2, PP3) |
|  |  |  | NM_000921.4(*PDE3A*):c.3284G>A (p.Arg1095Gln) | Het | 0.000262 | VUS (PM2, BP4) |
|  |  |  | NM_001143821.3(*PLEKHA5*):c.3389A>C (p.Glu1130Ala) | Het | 0.000298 | VUS (PM2) |
| 7851 | F |  | NM_000216.4(*ANOS1*):c.383T>C (p.Leu128Ser) | Het | 0.000024 | VUS (PM2, BP4) |
| 7852 | M |  | NM_032551.4(*KISS1R*):c.872C>T (p.Ala291Val) | Het | 0.000013 | VUS (PM2, BP4) |
|  |  |  | NM_003873.7(*NRP1*):c.1531T>C (p.Phe511Leu) | Het | 0.000025 | VUS (PM2, PP3, BS2) |
|  |  |  | NM_005045.3(*RELN*):c.2782A>G (p.Ile928Val) | Het | 0.000004 | VUS (PM2, PP2, BP4) |
| 7853 | F |  | NM_001080414.4(*CCDC88C*):c.6053C>T (p.Pro2018Leu) | Het | 0.000045 | VUS (PM2, BP4) |
|  |  |  | NM_000214.2(*JAG1*):c.5G>T (p.Arg2Leu) | Het | 0.000045 | VUS (PM2, PP2, BP4) |
|  |  |  | NM_014875.2(*KIF14*):c.1009G>A (p.Glu337Lys) | Het | 0.000134 | VUS (PM2, BP4) |
| 7860 | M |  | NM_019066.5(*MAGEL2*):c.2343G>T (p.Glu781Asp) | Het | 0.000008 | VUS (PM2) |
|  |  |  | NM_012431.2(*SEMA3E*):c.1296C>A (p.Asn432Lys) | Het | 0.000004 | VUS (PM2, BP4) |
| 7861 | M |  | NM_000163.4(*GHR*):c.1463C>T (p.Ala488Val) | Het | 0.000103 | VUS (PM2) |
|  |  |  | NM_014875.2(*KIF14*):c.2030C>T (p.Thr677Met) | Het | 0.00004 | VUS (PM2, PP3) |
| 7862 | F |  | NM_004004.6(*GJB2*):c.101T>C (p.Met34Thr) | Het | 0.008996 | LP (PM1, PM2, PM5, PP2, PP3, PP5) |
|  |  |  | NM_021252.3(*RAB18*):c.394G>T (p.Asp132Tyr) | Het | 0.000085 | VUS (PM2, PP3) |
| 7863 | F |  | NM_004491.5(*ARHGAP35*):c.2885G>C (p.Ser962Thr) | Het | 0.000759 | VUS (PM2, PP2, BP4) |
|  |  |  | NM_000168.5(*GLI3*):c.538C>T (p.Arg180Trp) | Het | 0.000008 | VUS (PM2, PM5) |
| 7868 | F |  | NM_022659.4(*EBF2*):c.1537_1539delTCA (p.Ser513del) | Het | 0.001882 | VUS (PM2, BS2, PM4) |
| 7870 | M |  | NM_000388.3(*CASR*):c.2265G>T (p.Glu755Asp) | Het | 0.000025 | VUS (PM1, PM2, PP2, BP6) |
|  |  |  | NM_004075.5(*CRY1*):c.1718C>T (p.Thr573Ile) | Het | 0.000004 | VUS (PM2, BP4) |
|  |  |  | NM_001143821.3(*PLEKHA5*):c.890G>C (p.Arg297Thr) | Het | - | VUS (PM2) |
|  |  |  | NM_001145358.2(*SIN3A*):c.17A>G (p.Asp6Gly) | Het | - | VUS (PM2, PP2, BP4) |
|  |  |  | NM_003193.3(*TBCE*):c.146A>C (p.His49Pro) | Het | 0.000032 | VUS (PM2, PP3) |
| 7873 | F |  | NM_005076.3(*CNTN2*):c.3014-2A>G | Het | - | VUS (PVS1, PM2) |
| 7878 | M |  | NM_023110.2(*FGRF1*):c.1368G>T (p.Met456Ile) | Het | 0.000409 | VUS (PP2, PP3, BP6) |
|  |  |  | NM_201266.1(*NRP2*):c.1412G>A (p.Arg471His) | Het | 0.000174 | VUS (PM2, PP3) |
|  |  |  | NM_006702.4(*PNPLA6*):c.3577C>G (p.Gln1193Glu) | Het | 0.000149 | VUS (PM2, PP2) |
|  |  |  | NM_018077.2(*RBM28*):c.1433A>G (p.Lys478Arg) | Het | - | VUS (PM2, BP4) |
| 7879 | M |  | NM_001273.3(*CHD4*):c.1064G>A (p.Gly355Asp) | Het | 0.000315 | VUS (PM2, PP2, PP3, BP6) |
|  |  |  | NM_001382323.2(*PKNOX2*):c.1141G>A (p.Ala381Thr) | Het | 0.000046 | VUS (PM2) |
|  |  |  | NM_001143821.3(*PLEKHA5*):c.2339A>G (p.Tyr780Cys) | Het | - | VUS (PM2) |
| 7880 | F |  | NM_003612.3(*SEMA7A*):c.1996C>T (p.His666Tyr) | Het | - | VUS (PM2, BP4) |
| 7883 | F |  | NM_152384.2(*BBS5*):c.751A>G (p.Asn251Asp) | Het | 0.001036 | VUS (BS1) |
|  |  |  | NM_017617.4(*NOTCH1*):c.5508C>A (p.Asp1836Glu) | Het | - | VUS (PM2, PP2) |
| 7884 | F |  | NM_005215.3(*DCC*):c.1904A>G (p.Asn635Ser) | Het | 0.000134 | VUS (PM2, BP4) |
|  |  |  | NM_000264.3(*PTCH1*):c.2015C>T (p.Thr672Met) | Het | 0.00002 | VUS (PM2, PP2, PP3, BP6) |
| 7885 | F |  | NM_001035235.2(*SRA1*):c.184C>A (p.Pro62Thr) | Het | 0.000004 | VUS (PM2, BP4) |
| 7887 | F |  | NM_178822.4(*IGSF10*):c.7334_7336dupTCA (p.Ile2445dup) | Het | 0.000173 | VUS (PM2, PM4) |
|  |  |  | NM_018117.11(*WDR11*):c.2305A>G (p.Met769Val) | Het | 0.000562 | VUS (BS1) |
| 7889 | M |  | NM_016952.4(*CDON*):c.3689T>A (p.Ile1230Asn) | Het | 0.000011 | VUS (PM2, BS2) |
|  |  |  | NM_003865.2(*HESX1*):c.102C>G (p.Asp34Glu) | Het | 0.000012 | VUS (PM2, PP2) |
|  |  |  | NM_003865.2(*HESX1*):c.134G>T (p.Trp45Leu) | Het | 0.000012 | VUS (PM2, PP2, PP3) |
| 7892 | M |  | NM_000479.3(*AMH*):c.1054C>T (p.Pro352Ser) | Het | 0.000476 | VUS |
|  |  |  | NM_017617.4(*NOTCH1*):c.2482G>A (p.Val828Met) | Het | 0.000018 | VUS (PM2, PP2) |
| 7893 | F |  | NM_021620.4(*PRDM13*):c.1883C>G (p.Thr628Arg) | Het | - | VUS (PM2) |
| 7894 | F |  | NM_003873.7(*NRP1*):c.2471T>C (p.Ile824Thr) | Het | 0.000213 | VUS (PM2) |
|  |  |  | NM_000439.4(*PCSK1*):c.1549C>T (p.Arg517*) | Het | 0.000012 | P (PVS1, PM2, PM3) |
| 7895 | M |  | NM_017617.4(*NOTCH1*):c.5476G>A (p.Glu1826Lys) | Het | 0.000028 | VUS (PM2, PP2) |
|  |  |  | NM_033661.4(*WDR4*):c.1148T>C (p.Leu383Pro) | Het | 0.000024 | VUS (PM2) |
|  |  |  | NM_033661.4(*WRD4*):c.1150G>A (p.Glu384Lys) | Het | 0.000024 | VUS (PM2, BP4) |
| 7896 | F |  | NM_001143821.3(*PLEKHA5*):c.2404G>A (p.Gly802Ser) | Het | 0.000214 | VUS (PM2) |
|  |  |  | NM_032242.3(*PLXNA1*):c.1474C>T (p.Leu492Phe) | Het | 0.000011 | VUS (PM2, PP2, BP4) |
|  |  |  | NM_007055.3(*POLR3A*):c.2993G>A (p.Arg998His) | Het | 0.000120 | VUS (PM2, PP2) |
| 7904 | F |  | NM_005270.4(*GLI2*):c.1445A>C (p.Lys482Thr) | Het | 0.000032 | VUS (PM2, PP3) |
|  |  |  | NM_178822.4(*IGSF10*):c.7334_7336dupTCA (p.Ile2445dup) | Het | 0.000173 | VUS (PM2, PM4) |
|  |  |  | NM_032551.4(*KISS1R*):c.872C>T (p.Ala291Val) | Het | 0.000013 | VUS (PM2, BP4) |
|  |  |  | NM_002303.5(*LEPR*):c.296G>A (p.Cys99Tyr) | Het | 0.000100 | VUS (PM2, BP6) |
| 7906 | M |  | NM_005270.4(*GLI2*):c.188C>T (p.Pro63Leu) | Het | 0.000042 | VUS (PM2, PP3) |
|  |  |  | NM_018848.3(*MKKS*):c.1474G>A (p.Asp492Asn) | Het | 0.000318 | VUS |
|  |  |  | NM_004958.3(*MTOR*):c.2323A>G (p.Ile775Val) | Het | - | VUS (PM2, PP2) |
| 7911 | M |  | NM_178822.4(*IGSF10*):c.1420A>G (p.Met474Val) | Het | 0.000028 | VUS (PM2, BP4) |
|  |  |  | NM_000439.4(*PCSK1*):c.2236G>A (p.Val746Met) | Het | 0.000035 | VUS (PM2, BP4) |
| 7914 | M |  | None |  |  |  |
| 7916 | M |  | NM_000115.4(*EDNRB*):c.167A>C (p.Lys56Thr) | Het | 0.000029 | VUS (PM2) |
|  |  |  | NM_005270.4(*GLI2*):c.3721A>C (p.Met1241Leu) | Het | 0.000111 | VUS (PM2, BP4) |
| 7920 | M |  | NM_000479.3(*AMH*):c.1303C>T (p.Arg435Cys) | Het | - | VUS (PM2) |
|  |  |  | NM_001080414.4(*CCDC88C*):c.41A>G (p.Gln14Arg) | Het | - | VUS (PM2, BP4) |
|  |  |  | NM_032242.3(*PLXNA1*):c.3679G>A (p.Glu1227Lys) | Het | 0.000017 | VUS (PM2, PP2) |
| 7923 | F |  | NM_002256.3(*KISS1*):c.154_156dupCCG (p.Pro52dup) | Het | 0.000030 | VUS (PM2, PM4) |
|  |  |  | NM_002673.4(*PLXNB1*):c.655G>A (p.Val219Met) | Het | 0.001526 | VUS (PM2, BS2, PP2) |
|  |  |  | NM_015910.7(*WDPCP*):c.1799delG (p.Arg600Leufs*12) | Het | 0.000012 | P (PVS1, PM2, PP5) |
| 7924 | F |  | NM_005634.2(*SOX3*):c.1059_1076delCACCGCCGCGGCCGCAGC (p.Thr354_Ala359del) | Het | - | VUS (PM2, BP3) |
| 7929 | M |  | NM_017780.3(*CHD7*):c.1056_1070delATTCCCATCAAACAG (p.Phe353_Ser357del) | Het | - | VUS (PM2, PM4) |
|  |  |  | NM_001174116.3(*DMXL2*):c.4987G>A (p.Ala1663Thr) | Het | 0.000078 | VUS (PM2, PP2) |
|  |  |  | NM_017617.4(*NOTCH1*):c.3190G>A (p.Asp1064Asn) | Het | 0.000060 | VUS (PM2, PP2, BP6) |
|  |  |  | NM_021620.4(*PRDM13*):c.1102C>G (p.Leu368Val) | Het | - | VUS (PM2, BP4) |
| 7934 | F |  | NM_000921.4(*PDE3A*):c.1375C>T (p.Arg459Trp) | Het | 0.000011 | VUS (PM1, PM2) |
|  |  |  | NM_018117.11(*WDR11*):c.2305A>G (p.Met769Val) | Het | 0.000562 | VUS (BS1) |
| 7935 | M |  | NM_001030055.2(*ARHGAP5*):c.3508A>G (p.Arg1170Gly) | Het | 0.000181 | VUS (PM2, PP2) |
|  |  |  | NM_024574.3(*NDNF*):c.653A>G (p.Glu218Gly) | Het | 0.000320 | VUS (PM2) |
|  |  |  | NM_003612.3(*SEMA7A*):c.709G>A (p.Asp237Asn) | Het | 0.000230 | VUS (PM2, BP4) |
|  |  |  | NM_003612.3(*SEMA7A*):c.1865G>A (p.Arg622His) | Het | 0.000404 | VUS (PM2, BP4) |
| 7943 | M |  | NM_004491.5(*ARHGAP35*):c.2230C>T (p.Arg744Cys) | Het | 0.000036 | VUS (PM2, PP2) |
|  |  |  | NM_173648.4(*CCDC141*):c.3321delA (p.Leu1107Phefs*12) | Het | 0.000292 | VUS (PM2) |
|  |  |  | NM_002633.2(*PGM1*):c.107C>A (p.Ala36Glu) | Het | 0.000105 | VUS |
|  |  |  | NM_018117.11(*WDR11*):c.3571G>A (p.Gly1191Ser) | Het | 0.000141 | VUS (PM2, PP3) |
| 7945 | F |  | NM_024649.5(*BBS1*):c.1318C>T (p.Arg440*) | Het | 0.000008 | P (PVS1, PM2, PM3) |
|  |  |  | NM_005215.3(*DCC*):c.744T>G (p.Asn248Lys) | Het | 0.000028 | VUS (PM2) |
|  |  |  | NM_004807.2(*HS6ST1*):c.1124G>A (p.Arg375His) | Het | 0.000212 | VUS (PM2) |
|  |  |  | NM_014875.2(*KIF14*):c.3192A>T (p.Glu1064Asp) | Het | - | VUS (PM2, BP4) |
|  |  |  | NM_000351.4(*STS*):c.1717G>A (p.Asp573Asn) | Het | - | VUS (PM2) |
|  |  |  | NM_006293.3(*TYRO3*):c.1790C>G (p.Pro597Arg) | Het | 0.000092 | VUS |
| 7949 | M |  | NM_016952.4(*CDON*):c.848C>T (p.Ala283Val) | Het | 0.000014 | VUS (PM2, BP4) |
|  |  |  | NM_000410.3(*HFE*):c.18G>C (p.Arg6Ser) | Het | 0.000684 | VUS |
|  |  |  | NM_004822.2(*NTN1*):c.1289C>T (p.Thr430Met) | Het | 0.000067 | VUS (PM2) |
| 7952 | F |  | NM_001273.3(*CHD4*):c.3088A>G (p.Met1030Val) | Het | 0.000099 | VUS (PP2) |
|  |  |  | NM_001174116.3(*DMXL2*):c.1318C>T (p.Arg440Trp) | Het | 0.000043 | VUS (PM2, PP2) |
|  |  |  | NM_207359.3(*GADL1*):c.1234G>T (p.Ala412Ser) | Het | 0.000004 | VUS (PM2) |
|  |  |  | NM_005599.3(*NHLH2*):c.283T>A (p.Leu95Met) | Het | - | VUS (PM2) |
| 7956 | F |  | NM_014875.2(*KIF14*):c.397T>C (p.Trp133Arg) | Het | - | VUS (PM2, BP4) |
| 7958 | M |  | NM_005996.3(*TBX3*):c.1242C>G (p.Asp414Glu) | Het | 0.000008 | VUS (PM2) |
|  |  |  | NM_004787.3(*SLIT2*):c.2875C>G (p.His959Asp) | Het | - | VUS (PM2, PP2) |
| 7959 | M |  | NM_000439.4(*PCSK1*):c.1460T>A (p.Ile487Asn) | Het | - | VUS (PM2) |
|  |  |  | NM_203290.2(*POLR1C*):c.34A>G (p.Ser12Gly) | Het | 0.000020 | VUS (PM2, PP2) |
| 7960 | M |  | NM_017514.4(*PLXNA3*):c.1204G>A (p.Glu402Lys) | hemi | 0.000067 | VUS (PM2, BS2) |
| 7964 | M |  | NM_002673.4(*PLXNB1*):c.1544C>T (p.Ser515Leu) | Het | 0.000171 | VUS (PM2, PP2, BP4) |
|  |  |  | NM_002673.4(*PLXNB1*):c.3911G>A (p.Arg1304His) | Het | 0.000053 | VUS (PM2, PP2, BP4) |
|  |  |  | NM_005045.3(*RELN*):c.7634C>T (p.Ala2545Val) | Het | 0.000216 | VUS (PP2, BP6) |
| 7968 | M |  | None |  |  |  |
| 7970 | F |  | NM_016952.4(*CDON*):c.458G>A (p.Arg153His) | Het | 0.000008 | VUS (PM2) |
|  |  |  | NM_001174116.3(*DMXL2*):c.1235G>A (p.Arg412Gln) | Het | 0.000024 | VUS (PM2, PP2, BP4) |
|  |  |  | NM_004958.3(*MTOR*):c.889G>A (p.Asp297Asn) | Het | 0.000078 | VUS (PM2, PP2, BP6) |
|  |  |  | NM_201266.1(*NRP2*):c.487C>T (p.Pro163Ser) | Het | 0.000004 | VUS (PM2) |
| 7971 | F |  | None |  |  |  |
| 7985 | M |  | NM_014875.2(*KIF14*):c.2648G>A (p.Arg883His) | Het | 0.000064 | VUS (PM2, BP4) |
|  |  |  | NM_032844.5(*MASTL*):c.2051C>T (p.Ser684Leu) | Het | 0.000020 | VUS (PM2) |
|  |  |  | NM_022370.3(*ROBO3*):c.3271T>C (p.Ser1091Pro) | Het | 0.000068 | VUS |
| 7986 | M |  | NM_000730.3(*CCKAR*):c.590G>A (p.Arg197His) | Het | 0.000014 | VUS (PM2) |
| 7992 | M |  | NM_015662.2(*IFT172*):c.2691G>T (p.Lys897Asn) | Het | - | VUS (PM2, BP4) |
|  |  |  | NM_002633.2(*PGM1*):c.1103C>T (p.Ala368Val) | Het | 0.000474 | VUS (BS1) |
|  |  |  | NM_017617.4(*NOTCH1*):c.5273G>A (p.Arg1758His) | Het | 0.000140 | VUS (PP2, BP6) |
| 7993 | F |  | NM_178822.4(*ISF10*):c.4187C>T (p.Ser1396Phe) | Het | 0.000233 | VUS (PM2, BP4) |
|  |  |  | NM_017617.4(*NOTCH1*):c.7498C>G (p.His2500Asp) | Het | 0.000033 | VUS (PM2, PP2, PP3) |
|  |  |  | NM_004959.4(*NR5A1*):c.754A>T (p.Thr252Ser) | Het | 0.000012 | VUS (PM1, PM2, PP2, BP4) |
| 7995 | F |  | NM_005045.3(*RELN*):c.7634C>T (p.Ala2545Val) | Het | 0.000216 | VUS (PP2, BP6) |
| 8002 | M |  | NM_005228.3(*EGFR*):c.3629C>T (p.Ala1210Val) | Het | 0.000304 | VUS (PM2) |
| 8003 | M |  | NM_001174116.3(*DMXL2*):c.8428G>C (p.Val2810Leu) | Het | - | VUS (PM2, PP2) |
|  |  |  | NM_015537.4(*NSMF*):c.241G>A (p.Gly81Ser) | Het | 0.000157 | VUS (PM2, BP4) |
| 8005 | F |  | NM_015662.2(*IFT172*):c.649C>T (p.Arg217Trp) | Het | 0.000032 | VUS (PM2) |
|  |  |  | NM_032242.3(*PLXNA1*):c.3629C>A (p.Ala1210Glu) | Het | - | VUS (PM2, PP2) |
|  |  |  | NM_006261.4(*PROP1*):c.301_302delAG (p.Leu102Cysfs*8) | Het | 0.000181 | P (PVS1, PS3, PM2, PM3, PP1) |
|  |  |  | NM_022370.3(*ROBO3*):c.2300C>A (p.Ala767Asp) | Het | 0.000047 | VUS (PM2, BP4) |
|  |  |  | NM_015910.7(*WDPCP*):c.1079C>T (p.Ser360Leu) | Het | 0.000171 | VUS (BS1) |
| 8014 | F |  | NM_001024613.3(*FEZF1*):c.752G>T (p.Gly251Val) | Het | - | VUS (PM2, BP4) |
| 8018 | F |  | NM_000216.4(*ANOS1*):c.727A>G (p.Thr243Ala) | Het | 0.000005 | VUS (PM2) |
|  |  |  | NM_017780.3(*CHD7*):c.1554G>T (p.Gln518His) | Het | 0.000016 | VUS (PM2, PP2, BP6) |
|  |  |  | NM_005215.3(*DCC*):c.527A>G (p.Asn176Ser) | Het | 0.000124 | VUS |
|  |  |  | NM_178822.4(*IGSF10*):c.467G>T (p.Arg156Leu) | Het | 0.000354 | VUS (PM2, PP5) |
|  |  |  | NM_000214.2(*JAG1*):c.494G>A (p.Arg165Gln) | Het | 0.000025 | VUS (PM2, PP2, BP6) |
|  |  |  | NM_030964.5(*SPRY4*):c.722C>A (p.Ser241Tyr) | Het | 0.004530 | VUS (PM2, BP6) |
|  |  |  | NM_018117.11(*WDR11*):c.2305A>G (p.Met769Val) | Het | 0.000562 | VUS (BS1) |
| 8021 | M |  | NM_031226.2(*CYP19A1*):c.578G>A (p.Arg193His) | Het | 0.000048 | VUS (PM2, BP4) |
| 8022 | M |  | NM_000479.3(*AMH*):c.1232G>A (p.Cys411Tyr) | Het | - | VUS (PM2) |
|  |  |  | NM_005076.3(*CNTN2*):c.2307G>C (p.Gln769His) | Het | - | VUS (PM2, PP2, BP4) |
|  |  |  | NM_018848.3(*MKKS*):c.1456T>C (p.Cys486Arg) | Het | - | VUS (PM2) |
| 8023 | M |  | NM_178822.4(*IGSF10*):c.6499G>A (p.Gly2167Arg) | Het | - | VUS (PM2, PP3) |
|  |  |  | NM_017617.4(*NOTCH1*):c.452A>G (p.Asn151Ser) | Het | 0.000017 | VUS (PM2, PP2, PP3) |
| 8024 | F |  | NM_015295.2(*SMCHD1*):c.5729A>G (p.Gln1910Arg) | Het | 0.000004 | VUS (PM2, PP2, BP4) |
|  |  |  | NM_001282748.2(*TLE4*):c.440C>T (p.Pro147Leu) | Het | - | VUS (PM2, PP2) |
| 8025 | F |  | NM_024649.5(*BBS1*):c.158dupA (p.Leu54Alafs*45) | Het | 0.000004 | P (PVS1, PM2, PP5) |
|  |  |  | NM_017563.4(*IL17RD*):c.1646T>G (p.Met549Arg) | Het | 0.000142 | VUS (PM2) |
|  |  |  | NM_174975.5(*SEC14L3*):c.1120G>A (p.Ala374Thr) | Het | 0.000272 | VUS (PM2, BP4) |
| 8026 | M |  | NM_015681.3(*B9D1*):c.376T>A (p.Ser126Thr) | Het | 0.000272 | VUS (PM2, BP6) |
|  |  |  | NM_000163.4(*GHR*):c.25A>G (p.Thr9Ala) | Het | 0.000032 | VUS (PM2) |
|  |  |  | NM_015537.4(*NSMF*):c.1330C>T (p.Arg444Cys) | Het | 0.000008 | VUS (PM2) |
|  |  |  | NM_016937.3(*POLA1*):c.1345A>G (p.Lys449Glu) | Hemi | - | VUS (PM2, BP4) |
| 8030 | F |  | NM_005235.2(*ERBB4*):c.1395C>A (p.Asn465Lys) | Het | 0.000085 | VUS (PM2) |
|  |  |  | NM_014875.2(*KIF14*):c.4057C>G (p.Gln1353Glu) | Het | 0.000065 | VUS (PM2) |
|  |  |  | NM_175737.3(*KLB*):c.2443A>G (p.Lys815Glu) | Het | 0.000978 | VUS (PM2, BP4) |
| 8041 | F |  | NM_007252.4(*POU6F2*):c.1885A>C (p.Asn629His) | Het | 0.000440 | VUS (PM2, BS2, PP3) |
| 8042 | F |  | NM_024574.3(*NDNF*):c.239C>T (p.Thr80Met) | Het | 0.001357 | VUS |
| 8044 | F |  | NM_001080414.4(*CCDC88C*):c.1669A>G (p.Lys557Glu) | Het | - | VUS (PM2, BP4) |
|  |  |  | NM_001102653.1(*OTUD4*):c.2755A>G (p.Thr919Ala) | Het | 0.000004 | VUS (PM2, BP4) |
| 8045 | F |  | NM_000479.3(*AMH*):c.802A>G (p.Thr268Ala) | Het | 0.000231 | VUS (PM2) |
|  |  |  | NM_207359.3(*GADL1*):c.1114G>T (p.Asp372Tyr) | Het | 0.001477 | VUS (BS2) |
|  |  |  | NM_022370.3(*ROBO3*):c.2461C>G (p.Arg821Gly) | Het | 0.000013 | VUS (PM2, BP4) |
| 8047 | F |  | NM_001170961.1(*IGSF1*):c.2807C>T (p.Thr936Ile) | Het | 0.000006 | VUS (PM2, BP4) |
|  |  |  | NM_015295.2(*SMCHD1*):c.424C>T (p.Pro142Ser) | Het | 0.000011 | VUS (PM1, PM2, PP2, PP3) |
|  |  |  | NM_015910.7(*WDPCP*):c.1429A>G (p.Lys477Glu) | Het | - | VUS (PM2) |
| 8051 | M |  | NM_203290.2(*POLR1C*):c.193A>G (p.Met65Val) | Het | 0.000916 | VUS (PP2, PP5, BS1) |
|  |  |  | NM_183059.2(*RD3*):c.223C>T (p.Pro75Ser) | Het | 0.000004 | VUS (PM2) |
|  |  |  | NM_006080.2(*SEMA3A*):c.196C>T (p.Arg66Trp) | Het | 0.000513 | VUS (PM2) |
| 8054 | F |  | NM_017780.3(*CHD7*):c.7253G>A (p.Arg2418Gln) | Het | 0.000068 | VUS (PM2, PM5, PP2, BP6) |
|  |  |  | NM_003612.3(*SEMA7A*):c.290T>A (p.Leu97His) | Het | 0.001274 | VUS (PM2, BP4) |
|  |  |  | NM_030964.5(*SPRY4*):c.841G>A (p.Val281Met) | Het | 0.000280 | VUS (PM2) |
| 8057 | F |  | NM_016952.4(*CDON*):c.791C>A (p.Pro264Gln) | Het | 0.000103 | VUS (PM2, BP6) |
|  |  |  | NM_020116.5(*FSTL5*):c.2029G>A (p.Val677Ile) | Het | - | VUS(PM2, BP4) |
|  |  |  | NM_020999.4(*NEUROG3*):c.17C>T (p.Ser6Leu) | Het | - | VUS (PM2) |
|  |  |  | NM_002673.4(*PLXNB1*):c.1186C>T (p.Pro396Ser) | Het | 0.001234 | VUS (PP2) |
|  |  |  | NM_004787.3(*SLIT2*):c.3095T>C (p.Leu1032Ser) | Het | 0.000018 | VUS (PM2, PP2, PP3) |
|  |  |  | NM_015910.7(*WDPCP*):c.160G>A (p.Asp54Asn) | Het | 0.000129 | VUS (PM2, PP3, PP5) |
| 8072 | M |  | NM_000730.3(*CCKAR*):c.799G>A (p.Asp267Asn) | Het | 0.000032 | VUS (PM2) |
|  |  |  | NM_005076.3(*CNTN2*):c.1276C>T (p.Arg426Cys) | Het | 0.000020 | VUS (PM2, PP2) |
|  |  |  | NM_178822.4(*IGSF10*):c.2161T>G (p.Tyr721Asp) | Het | - | VUS (PM2, BP4) |
|  |  |  | NM_018082.5(*POLR3B*):c.1931C>T (p.Ala644Val) | Het | 0.000012 | VUS (PM2, PP2, PP3) |
| 8075 | F |  | NM_015662.2(*IFT172*):c.178A>G (p.Met60Val) | Het | 0.000012 | VUS (PM2, BP4) |
|  |  |  | NM_000894.2(*LHB*):c.239G>A (p.Arg80His) | Het | 0.000032 | VUS (PM2) |
|  |  |  | NM_006378.3(*SEMA4D*):c.2393G>A (p.Ser798Asn) | Het | 0.000941 | VUS (PM2, BP4) |
| 8078 | M |  | NM_023110.2(*FGFR1*):c.584A>G (p.Lys195Arg) | Het | 0.000016 | VUS (PM2, PP2, BP4) |
| 8079 | F |  | NM_005076.3(*CNTN2*):c.821G>A (p.Arg274His) | Het | 0.000121 | VUS (PM2, PP2) |
|  |  |  | NM_022370.3(*ROBO3*):c.2021G>A (p.Arg674His) | Het | 0.000068 | VUS (PM2) |
|  |  |  | NM_031466.5(*TRAPPC9*):c.853C>T (p.Arg285Trp) | Het | 0.000453 | VUS |
| 8092 | F |  | NM_000479.3(*AMH*):c.553C>G (p.Gln185Glu) | Het | 0.000353 | VUS (PM2, BP6) |
|  |  |  | NM_201266.1(*NRP2*):c.1198G>A (p.Ala400Thr) | Het | 0.000088 | VUS (PM2) |
| 8093 | F |  | NM_000168.5(*GLI3*):c.314G>A (p.Arg105His) | Het | 0.000020 | VUS (PM2) |
|  |  |  | NM_015662.2(*IFT172*):c.2026G>A (p.Gly676Arg) | Het | 0.000024 | VUS (PM2) |
| 8094 | F |  | NM_001037293.3(*PALM2*):c.979G>A (p.Val327Met) | Het | 0.000025 | VUS (PM2, BP4) |
| 8095 | F |  | NM_024649.5(*BBS1*):c.725T>C (p.Met242Thr) | Het | 0.000036 | VUS (PM2) |
|  |  |  | NM_006614.2(*CHL1*):c.3437A>C (p.Asp1146Ala) | Het | - | VUS (PM2, PP3) |
|  |  |  | NM_000168.5(*GLI3*):c.557C>A (p.Ser186Tyr) | Het | - | VUS (PM2) |
| 8097 | F |  | NM_016937.3(*POLA1*):c.823A>G (p.Lys275Glu) | Het | - | VUS (PM2, BP4) |
|  |  |  | NM_007055.3(*POLR3A*):c.1744C>T (p.Arg582Cys) | Het | 0.000173 | VUS (PM2, PP2) |
| 8105 | M |  | NM_002673.4(*PLXNB1*):c.5707C>T (p.Leu1903Phe) | Het | 0.000040 | VUS (PM2, PP2) |
|  |  |  | NM_001059.2(*TACR3*):c.745A>G (p.Ile249Val) | Het | 0.000142 | VUS (PM2, BP4) |
|  |  |  | NM_031466.5(*TRAPPC9*):c.29C>G (p.Ala10Gly) | Het | - | VUS (PM2) |
| 8111 | F |  | NM_005228.3(*EGFR*):c.671G>A (p.Arg224His) | Het | 0.000032 | VUS (PM2) |
|  |  |  | NM_030964.5(*SPRY4*):c.722C>A (p.Ser241Tyr) | Het | 0.004530 | VUS (PM2, BP6) |
| 8112 | F |  | NM_001946.2(*DUSP6*):c.1037C>T (p.Thr346Met) | Het | 0.000286 | VUS (PM2, BP6) |
|  |  |  | NM_000515.3(*GH1*):c.7A>C (p.Thr3Pro) | Het | 0.000238 | VUS (PM2, BS2) |
|  |  |  | NM_007055.3(*POLR3A*):c.3301G>C (p.Val1101Leu) | Het | 0.000032 | VUS (PM2, PP2) |
| 8115 | F |  | NM_001174116.3(*DMXL2*):c.4379C>G (p.Thr1460Arg) | Het | 0.000014 | VUS (PM2, PP2, BP4) |
| 8118 | F |  | NM_004822.2(*NTN1*):c.116A>G (p.Asp39Gly) | Het | 0.000015 | VUS (PM2) |
|  |  |  | NM_032242.3(*PLXNA1*):c.1832A>G (p.Glu611Gly) | Het | 0.000012 | VUS (PM2, PP2, BP4) |
| 8121 | M |  | None |  |  |  |
| 8126 | F |  | NM_005076.3(*CNTN2*):c.3011G>A (p.Gly1004Glu) | Het | - | VUS (PM2, PP2, PP3) |
|  |  |  | NM_017617.4(*NOTCH1*):c.1682C>T (p.Thr561Met) | Het | 0.000102 | VUS (PM2, PP2, BP6) |
|  |  |  | NM_017617.4(*NOTCH1*):c.5273G>A (p.Arg1758His) | Het | 0.000140 | VUS (PP2, BP6) |
| 8128 | M |  | None |  |  |  |
| 8130 | F |  | NM_004004.6(*GJB2*):c.88A>G (p.Ile30Val) | Het | 0.000044 | LP (PM1, PM2, PM5, PP2, PP3) |
|  |  |  | NM_015537.4(*NSMF*):c.241G>A (p.Gly81Ser) | Het | 0.000157 | VUS (PM2, BP4) |
|  |  |  | NM_005045.3(*RELN*):c.5822T>C (p.Val1941Ala) | Het | 0.000358 | VUS (PP2, BP4) |
|  |  |  | NM_013251.3(*TAC3*):c.153C>G (p.Phe51Leu) | Het | 0.000004 | VUS (PM2, BP4) |
| 8131 | F |  | NM_005270.4(*GLI2*):c.3281C>T (p.Ser1094Phe) | Het | - | VUS (PM2, BP4) |
|  |  |  | NM_003873.7(*NRP1*):c.1775C>T (p.Pro592Leu) | Het | 0.000016 | VUS (PM2, PP3) |
| 8141 | F |  | NM_017617.4(*NOTCH1*):c.5273G>A (p.Arg1758His) | Het | 0.000140 | VUS (PP2, BP6) |
| 8149 | F |  | NM_207111.4(*RNF216*):c.91C>G (p.Pro31Ala) | Het | - | VUS (PM2, BP4) |
| 8152 | F |  | NM_015662.2(*IFT172*):c.1056C>G (p.His352Gln) | Het | 0.000042 | VUS (PM2, BP4) |
| 8163 | M |  | None |  |  |  |
| 8164 | F |  | NM_031466.5(*TRAPPC9*):c.2632C>G (p.Leu878Val) | Het | 0.000012 | VUS (PM2) |
| 8167 | F |  | NM_020999.4(*NEUROG3*):c.44G>A (p.Arg15His) | Het | 0.000036 | VUS (PM2, BP6) |
|  |  |  | NM_022370.3(*ROBO3*):c.2899C>T (p.Pro967Ser) | Het | 0.000128 | VUS (PM2) |
| 8168 | F |  | NM_213653.3(*HJV*):c.143C>G (p.Ser48Trp) | Het | - | VUS (PM2, PP2, BP4) |
| 8174 | F |  | NM_004491.5(*ARHGAP35*):c.3362A>G (p.Asn1121Ser) | Het | 0.000004 | VUS (PM2, PP2) |
|  |  |  | NM_000894.2(*LHB*):c.239G>A (p.Arg80His) | Het | 0.000032 | VUS (PM2) |
|  |  |  | NM_017514.5(*PLXNA3*):c.3034A>T (p.Ser1012Cys) | Het | 0.000097 | VUS (PM2, BS2) |
| 8177 | M |  | NM_198391.2(*FLRT3*):c.596C>G (p.Thr199Ser) | Het | 0.000014 | VUS (PM2, BP4) |
|  |  |  | NM_032242.3(*PLXNA1*):c.2261G>A (p.Arg754His) | Het | 0.000046 | VUS (PM2, PP2, BP4) |
|  |  |  | NM_174975.5(*SEC14L3*):c.1120G>A (p.Ala374Thr) | Het | 0.000272 | VUS (PM2, BP4) |
| 8178 | F |  | NM_001174116.3(*DMXL2*):c.4670C>T (p.Ser1557Leu) | Het | 0.000016 | VUS (PM2, PP2) |
|  |  |  | NM_004822.2(*NTN1*):c.1121A>G (p.Asn374Ser) | Het | 0.000021 | VUS (PM2) |
|  |  |  | NM_032242.3(*PLXNA1*):c.649G>A (p.Gly217Ser) | Het | 0.000035 | VUS (PM2, PP2, BP4) |
|  |  |  | NM_017514.5(*PLXNA3*):c.1357A>T (p.Thr453Ser) | Het | - | VUS (PM2, BP4) |
|  |  |  | NM_000264.3(*PTCH1*):c.2689A>G (p.Ile897Val) | Het | 0.000025 | VUS (PP2, PM2, BP6) |
|  |  |  | NM_031466.5(*TRAPPC9*):c.140G>A (p.Arg47Gln) | Het | 0.000315 | VUS (PM2, BP4) |
| 8179 | M |  | NM_018082.5(*POLR3B*):c.1931C>T (p.Ala644Val) | Het | 0.000012 | VUS (PM2, PP2, PP3) |
| 8180 | F |  | NM_173648.4(*CCDC141*):c.665G>A (p.Arg222His) | Het | 0.001087 | VUS (PM2, BP4) |
| 8181 | F |  | NM_024685.4(*BBS10*):c.137T>G (p.Leu46Arg) | Het | - | VUS (PM2, PP2, PP3) |
|  |  |  | NM_207359.3(*GADL1*):c.473C>T (p.Ala158Val) | Het | 0.000053 | VUS (PM2, BP4) |
|  |  |  | NM_017563.4(*IL17RD*):c.481G>A (p.Val161Ile) | Het | 0.000021 | VUS (PM2) |
|  |  |  | NM_005996.3(*TBX3*):c.1052G>T (p.Ser351Ile) | Het | 0.000023 | VUS (PM2) |
| 8182 | F |  | NM_001142699.1(*DLG2*):c.1001A>G (p.Asp334Gly) | Het | 0.000071 | VUS (PM2) |
|  |  |  | NM_014875.2(*KIF14*):c.1009G>A (p.Glu337Lys) | Het | 0.000134 | VUS (PM2, BP4) |
| 8184 | F |  | NM_173648.4(*CCDC141*):c.3091A>G (p.Thr1031Ala) | Het | 0.000016 | VUS (PM2) |
|  |  |  | NM_001174116.3(*DMXL2*):c.8065G>A (p.Asp2689Asn) | Het | - | VUS (PM2, PP2) |
| 8189 | M |  | NM_005221.5(*DLX5*):c.712C>A (p.His238Asn) | Het | 0.000195 | VUS (PM2) |
|  |  |  | NM_000515.3(*GH1*):c.493T>C (p.Phe165Leu) | Het | 0.000103 | VUS |
| 8195 | F |  | NM_173648.4(*CCDC141*):c.1600G>A (p.Val534Ile) | Het | 0.000545 | VUS (PM2, BP4) |
|  |  |  | NM_000168.5(*GLI3*):c.1354C>A (p.Gln452Lys) | Het | - | VUS (PM2, PP3) |
|  |  |  | NM_178822.4(*IGSF10*):c.5983G>A (p.Val1995Ile) | Het | 0.000772 | VUS (PM2, BS2) |
|  |  |  | NM_201266.1(*NRP2*):c.2552C>T (p.Ser851Leu) | Het | 0.000056 | VUS (PM2, BP4) |
|  |  |  | NM_017514.5(*PLXNA3*):c.3705C>G (p.Ile1235Met) | Het | - | VUS (PM2) |
| 8198 | M |  | NM_015662.2(*IFT172):c.704G>A (*p.Ser235Asn) | Het | - | VUS (PM2) |
|  |  |  | NM_019066.5(*MAGEL2*):c.1266_1286dupCATCCGCCCTGGCCCACCACC (p.Pro429_Val430insIleArgProGlyProProPro) | Het | 0.000067 | VUS (PM2, PM4) |
|  |  |  | NM_018344.5(*SLC29A3*):c.385G>A (p.Val129Ile) | Het | 0.000068 | VUS (PM2, BP4) |
| 8199 | F |  | None |  |  |  |
| 8203 | F |  | NM_004075.5(*CRY1*):c.199C>T (p.Arg67*) | Het | 0.000020 | VUS (PM2) |
|  |  |  | NM_198391.2(*FLRT3*):c.1255A>T (p.Thr419Ser) | Het | 0.000110 | VUS (PM2, BP4) |
|  |  |  | NM_004958.3(*MTOR*):c.3671A>G (p.Asp1224Gly) | Het | 0.000004 | VUS (PM2, PP2) |
| 8204 | M |  | NM_017563.4(*IL17RD*):c.604C>A (p.Pro202Thr) | Het | - | VUS (PM2) |
| 8206 | M |  | NM_021620.4(*PRDM13*):c.758A>T (p.Glu253Val) | Het | 0.000011 | VUS (PM2) |
| 8208 | M |  | NM_017563.4(*IL17RD*):c.1570C>G (p.His524Asp) | Het | 0.000025 | VUS (PM2, BP4) |
|  |  |  | NM_002256.3(*KISS1*):c.244C>A (p.Gln82Lys) | Het | 0.000161 | VUS (PM2, BP4) |
| 8212 | F |  | NM_004004.6(*GJB2*):c.670A>C (p.Lys224Gln) | Het | 0.000129 | VUS (PM2, PP2) |
|  |  |  | NM_017617.4(*NOTCH1*):c.6910T>G (p.Leu2304Val) | Het | 0.000008 | VUS (PM2, PP2, BP6) |
| 8215 | F |  | NM_001174116.3(*DMXL2*):c.1427C>G (p.Thr476Ser) | Het | 0.000124 | VUS (PM2, PP2, BP4) |
| 8216 | F |  | NM_021620.4(*PRDM13*):c.934T>C (p.Tyr312His) | Het | 0.000261 | VUS (PM2, BP4) |
|  |  |  | NM_003193.3(*TBCE*):c.1465C>A (p.Leu489Ile) | Het | 0.000520 | VUS (BP4) |
| 8218 | M |  | NM_003227.3(*TFR2*):c.1259G>A (p.Arg420His) | Het | 0.000170 | VUS (PM2) |
| 8221 | M |  | NM_001195306.2(*BBIP1*):c.135A>G (p.Ile45Met) | Het | 0.000012 | VUS (PM2, BP4) |
| 8226 | M |  | None |  |  |  |
| 8232 | F |  | NM_000479.3(*AMH*):c.556-2A>G | Het | - | LP (PVS1, PM2) |
|  |  |  | NM_001080414.4(*CCDC88C*):c.2819T>C (p.Leu940Pro) | Het | 0.000127 | VUS (PM2) |
|  |  |  | NM_000730.3(*CCKAR*):c.1006C>T (p.Arg336Trp) | Het | 0.000024 | VUS (PM2) |
|  |  |  | NM_018082.5(*POLR3B*):c.432C>G (p.Asn144Lys) | Het | 0.000007 | VUS (PM2, PP2) |
|  |  |  | NM_144773.3(*PROKR2*):c.253C>T (p.Arg85Cys) | Het | 0.000601 | LP (PM1, PM2, PM5, PP5) |
| 8233 | M |  | NM_017617.4(*NOTCH1*):c.6910T>G (p.Leu2304Val) | Het | 0.000008 | VUS (PM2, PP2, BP6) |
|  |  |  | NM_032242.3(*PLXNA1*):c.329G>A (p.Ser110Asn) | Het | 0.000823 | VUS (PM2, PP2, BP4) |
| 8234 | F |  | NM_018848.3(*MKKS*):c.721G>T (p.Val241Leu) | Het | 0.000012 | VUS (PM2) |
| 8235 | F |  | NM_016952.4(*CDON*):c.3367A>G (p.Lys1123Glu) | Het | 0.000008 | VUS (PM2) |
|  |  |  | NM_003227.3(*TFR2*):c.840C>G (p.Phe280Leu) | Het | 0.000397 | VUS |
| 8237 | M |  | NM_000921.4(*PDE3A*):c.617T>C (p.Leu206Pro) | Het | 0.000366 | VUS (PM2) |
|  |  |  | NM_032242.3(*PLXNA1*):c.3460C>T (p.Pro1154Ser) | Het | 0.00006 | VUS (PM2, PP2, BP4) |
| 8238 | F |  | NM_005270.4(*GLI2*):c.4628G>A (p.Arg1543His) | Het | 0.000534 | VUS (BP6) |
|  |  |  | NM_000406.2(*GNRHR*):c.317A>G (p.Gln106Arg) | Het | 0.002749 | P (PM1, PP2, PM2, PP5) |
|  |  |  | NM_015295.2(*SMCHD1*):c.3527C>T (p.Thr1176Ile) | Het | 0.000304 | VUS (PP2, BP4) |
| 8239 | M |  | NM_203290.2(*POLR1C*):c.193A>G (p.Met65Val) | Het | 0.000916 | VUS (PP2, PP5, BS1) |
| 8245 | F |  | NM_207359.3(*GADL1*):c.1234G>T (p.Ala412Ser) | Het | 0.000004 | VUS (PM2) |
|  |  |  | NM_032242.3(*PLXNA1*):c.1528G>T (p.Val510Leu) | Het | 0.000005 | VUS (PM2, PP2, BP4) |
|  |  |  | NM_006702.4(*PNPLA6*):c.3611C>T (p.Ala1204Val) | Het | 0.000028 | VUS (PM2, PP2, BP4) |
| 8253 | M |  | NM_001080414.4(*CCDC88C*):c.5899G>A (p.Gly1967Arg) | Het | 0.000015 | VUS (PM2, BP4) |
|  |  |  | NM_001873.3(*CPE*):c.1309G>A (p.Val437Ile) | Het | 0.000539 | VUS (PM2, BP4) |
|  |  |  | NM_033163.3(*FGF8*):c.77C>T (p.Pro26Leu) | Het | 0.001149 | VUS (PP2, BS1, BP6) |
|  |  |  | NM_014875.2(*KIF14*):c.1009G>A (p.Glu337Lys) | Het | 0.000134 | VUS (PM2, BP4) |
| 8265 | M |  | NM_017780.3(*CHD7*):c.2787C>G (p.Ile929Met) | Het | 0.000018 | VUS (PM2, PP2, BP6) |
|  |  |  | NM_031466.5(*TRAPPC9*):c.2354T>G (p.Val785Gly) | Het | 0.000007 | VUS (PM2, BP4) |
| 8266 | M |  | None |  |  |  |
| 8278 | M |  | NM_178822.4(*IGSF10*):c.1792A>G (p.Thr598Ala) | Het | 0.000004 | VUS (PM2, BP4) |
|  |  |  | NM_017617.4(*NOTCH1*):c.7223T>C (p.Leu2408Pro) | Het | 0.000096 | VUS (PP2, BP6) |
| 8282 | M |  | NM_005270.4(*GLI2*):c.4018G>C (p.Val1340Leu) | Het | 0.000033 | VUS (PM2, BP4) |
|  |  |  | NM_005045.3(*RELN*):c.6770C>T (p.Ser2257Leu) | Het | 0.00004 | VUS (PP2, PM2) |
| 8294 | F |  | NM_024685.4(*BBS10*):c.1634C>T (p.Ser545Phe) | Het | 0.000004 | VUS (PM2, PP2) |
|  |  |  | NM_001382323.2(*PKNOX2*):c.210C>G (p.Asp70Glu) | Het | 0.000004 | VUS (PM2) |
| 8296 | M |  | NM_001030055.2(*ARHGAP5*):c.3340G>A (p.Asp1114Asn) | Het | - | VUS (PM2, PP2) |
|  |  |  | NM_020999.4(*NEUROG3*):c.367G>C (p.Glu123Gln) | Het | 0.000074 | VUS (PM2, PP3) |
|  |  |  | NM_001382323.2(*PKNOX2*):c.1339G>A (p.Glu447Lys) | Het | - | VUS (PM2) |
| 8298 | M |  | NM_177976.1(*ARL6*):c.30G>C (p.Leu10Phe) | Het | - | VUS (PM2, PP2, BP4) |
|  |  |  | NM_017780.3(*CHD7*):c.2830C>T (p.Arg944Cys) | Het | 0.000028 | LP (PM2, PM5, PP2, PP3) |
| 8304 | M |  | NM_004491.5(*ARHGAP35*):c.1090A>G (p.Ile364Val) | Het | 0.000111 | VUS (PM2, PP2, BP4) |
|  |  |  | NM_001174116.3(*DMXL2*):c.4540C>G (p.Leu1514Val) | Het | - | VUS (PM2, PP2) |
|  |  |  | NM_004439.5(*EPHA5*):c.2134G>A (p.Gly712Ser) | Het | - | VUS (PM2) |
| 8306 | F |  | NM_002633.2(*PGM1*):c.107C>A (p.Ala36Glu) | Het | 0.000105 | VUS |
|  |  |  | NM_016937.3(*POLA1*):c.3731C>T (p.Thr1244Ile) | Het | - | VUS (PM2, BP4) |

id, identification; M, male; F, female; Het, heterozygous; Homo, homozygous; Hemi, hemizygous; GnomAD, Genome Aggregation Database; ACMG, American College of Medical Genetics and Genomics. Variants were classified as Pathogenic (P), Likely Pathogenic (LP), Variants of Uncertain Significance (VUS), Likely Benign (LB), or Benign (B), based on the evidence for pathogenicity [very strong (PVS1), strong (PS1-4), moderate (PM1–6), or supporting (PP1–5)] or benign impact [stand-alone (BA), strong (BS1-4), or supporting (BP1-7)]. PMID, PubMed identifier.
