# Supplementary material for: Genetic architecture of congenital hypogonadotropic hypogonadism: insights from analysis of a Portuguese cohort
Source: Hum Reprod Open. 2024 Sep 11;2024(3):hoae053. doi: 10.1093/hropen/hoae053 (PMC11415827; doi:10.1093/hropen/hoae053)
Supplement: hoae053_Supplementary_Data [file hoae053_supplementary_data.zip › Supplementary Table S1.docx]

**Supplementary Table S1.** Genes implicated in CHH and selected for variant analysis.

| **Gene symbol** | **Reference transcript** | **Phenotype and mode of inheritance in OMIM** | **First report of association with CHH** |
| --- | --- | --- | --- |
| *AMH* | NM_000479.3 | Persistent Mullerian duct syndrome, type I - AR | PMID: 31291191 |
| *AMHR2* | NM_020547.3 | Persistent Mullerian duct syndrome, type II - AR | PMID: 31291191 |
| *AMN1* | NM_001113402.2 | - | PMID: 27502037 |
| *ANOS1* | NM_000216.4 | Hypogonadotropic hypogonadism 1 with or without anosmia (Kallmann syndrome 1) - XLR | PMID: 1913827 |
| *ARHGAP5* | NM_001030055.2 | - | PMID: 36178483 |
| *ARHGAP35* | NM_004491.5 | - | PMID: 36178483 |
| *ARL6* | NM_177976.1 | Bardet-Biedl syndrome 3 - AR; Retinitis pigmentosa 55 - AR; {Bardet-Biedl syndrome 1, modifier of} - AR/DR | PMID: 15258860 |
| *AXL* | NM_021913.3 | - | PMID: 24476074 |
| *B4GAT1* | NM_006876.2 | Muscular dystrophy-dystroglycanopathy (congenital with brain and eye anomalies), type A, 13 - AR | PMID: 30098700 |
| *B9D1* | NM_015681.3 | ?Meckel syndrome 9 - AR; Joubert syndrome 27 - AR | PMID: 29165578 |
| *BBIP1* | NM_001195306.2 | Bardet-Biedl syndrome 18 - AR | PMID:37239474 |
| *BBS1* | NM_024649.5 | Bardet-Biedl syndrome 1 - AR, DR | PMID:37239474 |
| *BBS5* | NM_152384.2 | Bardet-Biedl syndrome 5 - AR | PMID: 24559376 |
| *BBS10* | NM_024685.4 | Bardet-Biedl syndrome 10 - AR | PMID: 23403234 |
| *CASR* | NM_000388.3 | Hyperparathyroidism, neonatal - AD/AR; Hypocalcemia, autosomal dominant - AD; Hypocalcemia, autosomal dominant, with Bartter syndrome - AD; Hypocalciuric hypercalcemia, type I - AD | PMID: 30098700 |
| *CCDC141* | NM_173648.4 | - | PMID: 25192046 |
| *CCDC88C* | NM_001080414.4 | ?Spinocerebellar ataxia 40 - AD; Hydrocephalus, congenital, 1 - AR | PMID: 33208564 |
| *CCKAR* | NM_000730.3 | - | PMID: 30098700 |
| *CCKBR* | NM_176875.2 | - | PMID: 27502037 |
| *CDKN1C* | NM_000076.2 | Beckwith-Wiedemann syndrome - AD; IMAGE syndrome - AD | PMID: 22634751 |
| *CDON* | NM_016952.4 | Holoprosencephaly 11 - AD | PMID: 33208564 |
| *CHD4* | NM_001273.3 | Sifrim-Hitz-Weiss syndrome - AD | PMID: 31388190 |
| *CHD7* | NM_017780.3 | CHARGE syndrome - AD; Hypogonadotropic hypogonadism 5 with or without anosmia - AD | PMID: 18834967 |
| *CHL1* | NM_006614.2 | - | PMID: 33453020 |
| *CNTN2* | NM_005076.3 | ?Epilepsy, myoclonic, familial adult, 5 - AR | PMID: 30098700 |
| *CPE* | NM_001873.3 | BDV syndrome - AR | PMID: 26120850 |
| *CRY1* | NM_004075.5 | Delayed sleep phase disorder, susceptibility to - AD | PMID: 27502037 |
| *CXCR4* | NM_003467.2 | Myelokathexis, isolated - AD; WHIM syndrome 1- AD | PMID: 27502037 |
| *CYP19A1* | NM_031226.2 | Aromatase excess syndrome – AD; Aromatase deficiency – AR | PMID: 12736278 |
| *DCAF17* | NM_025000.3 | Woodhouse-Sakati syndrome - AR | PMID: 20507343 |
| *DCC* | NM_005215.3 | Gaze palsy, familial horizontal, with progressive scoliosis, 2 - AR; Mirror movements 1 and/or agenesis of the corpus callosum - AD | PMID: 29202173 |
| *DLG2* | NM_001142699.1 | - | PMID: 32341572 |
| *DLX5* | NM_005221.5 | Split-hand/foot malformation 1 with sensorineural hearing loss - AR; Split-hand/foot malformation 1 - AD | PMID: 30098700 |
| *DMXL2* | NM_001174116.3 | Deafness, autosomal dominant 71 - AD; Polyendocrine-polyneuropathy syndrome - AR; Developmental and epileptic encephalopathy 81 - AR | PMID: 33208564 |
| *DUSP6* | NM_001946.2 | Hypogonadotropic hypogonadism 19 with or without anosmia -- AD | PMID: 23643382 |
| *EBF2* | NM_022659.4 | - | PMID: 30098700 |
| *EDNRB* | NM_000115.4 | ABCD syndrome - AR; Waardenburg syndrome, type 4A - AD, AR; Hirschsprung disease, susceptibility to, 2 - AD | PMID: 30098700 |
| *EFNA5* | NM_001962.3 | - | PMID: 30098700 |
| *EGF* | NM_001963.5 | Hypomagnesemia 4, renal - AR | PMID: 30098700 |
| *EGFR* | NM_005228.3 | Inflammatory skin and bowel disease, neonatal, 2 - AR; Adenocarcinoma of lung, response to tyrosine kinase inhibitor in - AD; Nonsmall cell lung cancer, response to tyrosine kinase inhibitor in - AD; Nonsmall cell lung cancer, susceptibility to - AD | PMID: 30098700 |
| *EPHA5* | NM_004439.5 | - | PMID: 30098700 |
| *ERBB4* | NM_005235.2 | Amyotrophic lateral sclerosis 19 - AD | PMID: 30098700 |
| *FEZ1* | NM_005103.5 | - | PMID: 30098700 |
| *FEZF1* | NM_001024613.3 | Hypogonadotropic hypogonadism 22, with or without anosmia - AR | PMID: 25192046 |
| *FGF13* | NM_004114.5 | Developmental and epileptic encephalopathy 90 - XLD, XLR; Intellectual developmental disorder, X-linked 110 - XLR | PMID: 27502037 |
| *FGF17* | NM_003867.2 | Hypogonadotropic hypogonadism 20 with or without anosmia - AD | PMID: 23643382 |
| *FGF8* | NM_033163.3 | Hypogonadotropic hypogonadism 6 with or without anosmia - AD | PMID: 18596921 |
| *FGFR1* | NM_023110.2 | Hartsfield syndrome - AD; Hypogonadotropic hypogonadism 2 with or without anosmia - AD; Jackson-Weiss syndrome - AD; Osteoglophonic dysplasia - AD; Pfeiffer syndrome - AD; Trigonocephaly 1 - AD | PMID: 12627230 |
| *FLRT3* | NM_198391.2 | Hypogonadotropic hypogonadism 21 with anosmia - AD | PMID: 23643382 |
| *FSHB* | NM_000510.2 | Hypogonadotropic hypogonadism 24 without anosmia - AR | PMID: 8220432 |
| *FSTL5* | NM_020116.5 | - | PMID: 32763379 |
| *GADL1* | NM_207359.3 | - | PMID: 33208564 |
| *GAP43* | NM_001130064.1 | - | PMID: 27502037 |
| *GH1* | NM_000515.3 | Growth hormone deficiency, isolated, type IA - AR; Growth hormone deficiency, isolated, type II - AD; Kowarski syndrome - AR | PMID: 30098700 |
| *GHR* | NM_000163.4 | Growth hormone insensitivity, partial - AD; Increased responsiveness to growth hormone - AD; Laron dwarfism - AR; Hypercholesterolemia, familial, modifier of - AD, AR | PMID: 30098700 |
| *GJB2* | NM_004004.6 | Bart-Pumphrey syndrome - AD; Deafness, autosomal dominant 3A - AD; Deafness, autosomal recessive 1A - AR, DD (digenic dominant); Hystrix-like ichthyosis with deafness - AD; Keratitis-ichthyosis-deafness syndrome - AD; Keratoderma, palmoplantar, with deafness - AD; Vohwinkel syndrome - AD | PMID: 11874189 |
| *GLI2* | NM_005270.4 | Culler-Jones syndrome - AD; Holoprosencephaly 9 - AD | PMID: 25327282 |
| *GLI3* | NM_000168.5 | Greig cephalopolysyndactyly syndrome - AD; Pallister-Hall syndrome - AD; Polydactyly, postaxial, types A1 and B - AD; Polydactyly, preaxial, type IV - AD | PMID: 27502037 |
| *GNRH1* | NM_000825.3 | Hypogonadotropic hypogonadism 12 with or without anosmia - AR | PMID: 19567835 |
| *GNRHR* | NM_000406.2 | Hypogonadotropic hypogonadism 7 without anosmia - AR | PMID: 9371856 |
| *HDAC8* | NM_018486.2 | Cornelia de Lange syndrome 5 - XLD | PMID: 22889856 |
| *HESX1* | NM_003865.2 | Growth hormone deficiency with pituitary anomalies - AD, AR; Pituitary hormone deficiency, combined, 5 - AD, AR; Septooptic dysplasia - AD, AR | PMID: 23465708 |
| *HFE* | NM_000410.3 | Hemochromatosis - AR; Alzheimer disease, susceptibility to - AD; Porphyria cutanea tarda, susceptibility to - AD, AR; Porphyria variegata, susceptibility to - AD | PMID: 15657376 |
| *HGF* | NM_000601.4 | Deafness, autosomal recessive 39 - AR | PMID: 30098700 |
| *HJV* | NM_213653.3 | Hemochromatosis, type 2A - AR | PMID: 15811010 |
| *HS6ST1* | NM_004807.2 | Hypogonadotropic hypogonadism 15 with or without anosmia - AD | PMID: 21700882 |
| *IFT172* | NM_015662.2 | Bardet-Biedl syndrome 20 - AR; Retinitis pigmentosa 71 - AR; Short-rib thoracic dysplasia 10 with or without polydactyly - AR | PMID: 26763875 |
| *IGF1* | NM_000618.3 | Growth retardation with deafness and mental retardation due to IGF1 deficiency - AR | PMID: 30098700 |
| *IGSF1* | NM_001170961.1 | Hypothyroidism, central, and testicular enlargement - XLR | PMID: 31200363 |
| *IGSF10* | NM_178822.4 | - | PMID: 27137492 |
| *IL17RD* | NM_017563.4 | Hypogonadotropic hypogonadism 18 with or without anosmia - AD, AR, DD | PMID: 23643382 |
| *JAG1* | NM_000214.2 | Deafness, congenital heart defects, and posterior embryotoxon - AD; Alagille syndrome 1 - AD; Charcot-Marie-Tooth disease, axonal, type 2HH - AD; Tetralogy of Fallot - AD | PMID: 27502037 |
| *KIF14* | NM_014875.2 | Meckel syndrome 12 - AR; Microcephaly 20, primary, autosomal recessive - AR | PMID: 29165578 |
| *KISS1* | NM_002256.3 | Hypogonadotropic hypogonadism 13 with or without anosmia - AR | PMID: 22335740 |
| *KISS1R* | NM_032551.4 | Precocious puberty, central, 1 - AD; Hypogonadotropic hypogonadism 8 with or without anosmia - AR | PMID: 12944565 |
| *KLB* | NM_175737.3 | - | PMID: 28754744 |
| *LEP* | NM_000230.2 | Obesity, morbid, due to leptin deficiency - AR | PMID: 9500540 |
| *LEPR* | NM_002303.5 | Obesity, morbid, due to leptin receptor deficiency - AR | PMID: 9537324 |
| *LHB* | NM_000894.2 | Hypogonadotropic hypogonadism 23 with or without anosmia - AR | PMID: 1727547 |
| *LHX3* | NM_014564.4 | Pituitary hormone deficiency, combined, 3 - AR | PMID: 10835633 |
| *LHX4* | NM_033343.3 | Pituitary hormone deficiency, combined, 4 - AD | PMID: 16735499 |
| *LIF* | NM_002309.3 | - | PMID: 30098700 |
| *LZTFL1* | NM_020347.4 | Bardet-Biedl syndrome 17 - AR | PMID:37239474 |
| *MAGEL2* | NM_019066.5 | Schaaf-Yang syndrome - AD | PMID: 24076603 |
| *MASTL* | NM_032844.5 | - | PMID: 27502037 |
| *MC4R* | NM_005912.2 | Obesity - AD, AR; Obesity, resistance to - AD, AR | PMID: 21921657 |
| *MET* | NM_001127500.1 | Arthrogryposis, distal, type 11 - AD; Deafness, autosomal recessive 97 - AR; Osteofibrous dysplasia, susceptibility to - AD | PMID: 30098700 |
| *MKKS* | NM_018848.3 | Bardet-Biedl syndrome 6 -AR; McKusick-Kaufman syndrome - AR | PMID:37239474 |
| *MTOR* | NM_004958.3 | Smith-Kingsmore syndrome - AD | PMID: 30098700 |
| *NDNF* | NM_024574.3 | Hypogonadotropic hypogonadism 25 with anosmia - AD | PMID: 31883645 |
| *NEUROG3* | NM_020999.4 | Diarrhea 4, malabsorptive, congenital - AR | PMID: 27533310 |
| *NHLH2* | NM_005599.3 | Hypogonadotropic hypogonadism 27 without anosmia - AR | PMID: 35066646 |
| *NOS1* | NM_000620.4 | - | PMID: 27502037 |
| *NOTCH1* | NM_017617.4 | Adams-Oliver syndrome 5 - AD; Aortic valve disease 1 - AD | PMID: 27502037 |
| *NR0B1* | NM_000475.4 | 46XY sex reversal 2, dosage-sensitive - XL; Adrenal hypoplasia, congenital - XLR | PMID: 7990958 |
| *NR5A1* | NM_004959.4 | 46XX sex reversal 4 - AD; 46XY sex reversal 3 - AD; Adrenocortical insufficiency - AD; Premature ovarian failure 7 - AD; Spermatogenic failure 8 - AD | PMID: 28326187 |
| *NRP1* | NM_003873.7 | - | PMID: 30098700 |
| *NRP2* | NM_201266.1 | - | PMID: 27502037 |
| *NSMF* | NM_015537.4 | Hypogonadotropic hypogonadism 9 with or without anosmia - AD | PMID: 15362570 |
| *NTN1* | NM_004822.2 | Mirror movements 4 - AD | PMID: 29202173 |
| *OTUD4* | NM_001102653.1 | - | PMID: 23656588 |
| *OTX2* | NM_172337.1 | Microphthalmia, syndromic 5 - AD; Pituitary hormone deficiency, combined, 6 - AD; Retinal dystrophy, early-onset, with or without pituitary dysfunction - AD | PMID: 31200363 |
| *PALM2* | NM_001037293.3 | - | PMID: 27502037 |
| *PAX6* | NM_000280.3 | Coloboma of optic nerve - AD; Coloboma, ocular - AD; Morning glory disc anomaly - AD; Aniridia - AD; Anterior segment dysgenesis 5, multiple subtypes - AD; Cataract with late-onset corneal dystrophy - AD; Foveal hypoplasia 1 - AD; Keratitis - AD; Optic nerve hypoplasia - AD | PMID: 30098700 |
| *PCSK1* | NM_000439.4 | Endocrinopathy due to proprotein convertase 1/3 deficiency - AR | PMID: 9207799 |
| *PDE3A* | NM_000921.4 | Hypertension and brachydactyly syndrome - AD | PMID: 27502037 |
| *PGM1* | NM_002633.2 | Congenital disorder of glycosylation, type It - AR | PMID: 24499211 |
| *PHF6* | NM_032458.3 | Borjeson-Forssman-Lehmann syndrome - XLR | PMID:12415272 |
| *PKNOX2* | NM_001382323.2 | - | PMID: 32763379 |
| *PLEKHA5* | NM_001143821.3 | - | PMID: 27502037 |
| *PLXNA1* | NM_032242.3 | Dworschak-Punetha neurodevelopmental syndrome - AR | PMID: 30098700 |
| *PLXNA3* | NM_017514.5 | - | PMID: 30661757 |
| *PLXNB1* | NM_002673.4 | - | PMID: 30098700 |
| *PNPLA6* | NM_006702.4 | Laurence-Moon syndrome - AR; Boucher-Neuhauser syndrome - AR; Oliver-McFarlane syndrome - AR; Spastic paraplegia 39, autosomal recessive - AR | PMID: 24355708 |
| *POGZ* | NM_015100.4 | White-Sutton syndrome - AD | PMID: 37619992 |
| *POLA1* | NM_016937.3 | Pigmentary disorder, reticulate, with systemic manifestations, X-linked - XLR; Van Esch-O'Driscoll syndrome - XLR | PMID: 31006512 |
| *POLR1C* | NM_203290.2 | Leukodystrophy, hypomyelinating, 11 - AR; Treacher Collins syndrome 3 - AR | PMID: 33005949 |
| *POLR3A* | NM_007055.3 | Leukodystrophy, hypomyelinating, 7, with or without oligodontia and/or hypogonadotropic hypogonadism - AR; Wiedemann-Rautenstrauch syndrome - AR | PMID: 21855841 |
| *POLR3B* | NM_018082.5 | Charcot-Marie-Tooth disease, demyelinating, type 1I - AD; Leukodystrophy, hypomyelinating, 8, with or without oligodontia and/or hypogonadotropic hypogonadism - AR | PMID: 22036172 |
| *POU6F2* | NM_007252.4 | {Wilms tumor susceptibility-5} - AD, SMu | PMID:37600690 |
| *PRDM13* | NM_021620.4 | Cerebellar dysfunction, impaired intellectual development, and hypogonadotropic hypogonadism - AR; Pontocerebellar hypoplasia, type 17 - AR | PMID: 34730112 |
| *PROK2* | NM_001126128.1 | Hypogonadotropic hypogonadism 4 with or without anosmia - AD | PMID: 17054399 |
| *PROKR2* | NM_144773.3 | Hypogonadotropic hypogonadism 3 with or without anosmia - AD | PMID: 17054399 |
| *PROP1* | NM_006261.4 | Pituitary hormone deficiency, combined, 2 - AR | PMID: 9462743 |
| *PTCH1* | NM_000264.3 | Basal cell nevus syndrome 1 - AD; Holoprosencephaly 7 - AD | PMID: 32074614 |
| *RAB18* | NM_021252.3 | Warburg micro syndrome 3 - AR | PMID: 21473985 |
| *RAB3GAP1* | NM_012233.3 | Martsolf syndrome 2 - AR; Warburg micro syndrome 1 - AR | PMID: 15696165 |
| *RAB3GAP2* | NM_012414.3 | Martsolf syndrome 1 - AR; Warburg micro syndrome 2 - AR | PMID: 16532399 |
| *RBM28* | NM_018077.2 | Alopecia, neurologic defects, and endocrinopathy syndrome - AR | PMID: 18439547 |
| *RD3* | NM_183059.2 | Leber congenital amaurosis 12 - AR | PMID: 27502037 |
| *RELN* | NM_005045.3 | Lissencephaly 2 (Norman-Roberts type) - AR; Epilepsy, familial temporal lobe, 7- AD | PMID: 30098700 |
| *RNF216* | NM_207111.4 | Cerebellar ataxia and hypogonadotropic hypogonadism - AR | PMID: 23656588 |
| *ROBO3* | NM_022370.3 | Gaze palsy, familial horizontal, with progressive scoliosis, 1 - AR | PMID: 30098700 |
| *SEC14L3* | NM_001376914.1 | - | PMID: 32763379 |
| *SEMA3A* | NM_006080.2 | Hypogonadotropic hypogonadism 16 with or without anosmia - AD | PMID: 22927827 |
| *SEMA3E* | NM_012431.2 | - | PMID: 25985275 |
| *SEMA4D* | NM_006378.3 | - | PMID: 30098700 |
| *SEMA7A* | NM_003612.3 | Cholestasis, progressive familial intrahepatic, 11 - AR | PMID: 24522099 |
| *SIN3A* | NM_001145358.2 | Witteveen-Kolk syndrome - AD | PMID: 36758531 |
| *SLC29A3* | NM_018344.5 | Histiocytosis-lymphadenopathy plus syndrome - AR | PMID: 20619369 |
| *SLIT2* | NM_004787.3 | - | PMID: 30098700 |
| *SMCHD1* | NM_015295.2 | Bosma arhinia microphthalmia syndrome - AD; Fascioscapulohumeral muscular dystrophy 2, digenic - DD | PMID: 28546579 |
| *SOX10* | NM_006941.3 | PCWH syndrome - AD; Waardenburg syndrome, type 2E, with or without neurologic involvement - AD; Waardenburg syndrome, type 4C - AD | PMID: 23643381 |
| *SOX11* | NM_003108.4 | Intellectual developmental disorder with microcephaly and with or without ocular malformations or hypogonadotropic hypogonadism - AD | PMID: 35341651 |
| *SOX2* | NM_003106.3 | Microphthalmia, syndromic 3 - AD; Optic nerve hypoplasia and abnormalities of the central nervous system - AD | PMID: 16932809 |
| *SOX3* | NM_005634.2 | Panhypopituitarism, X-linked - XL | PMID: 25064402 |
| *SPRED3* | NM_001394336.1 | - | PMID: 33208564 |
| *SPRY4* | NM_030964.5 | Hypogonadotropic hypogonadism 17 with or without anosmia - AD | PMID: 23643382 |
| *SRA1* | NM_001035235.2 | - | PMID: 27086651 |
| *STS* | NM_000351.4 | Ichthyosis, X-linked - XLR | PMID: 25597551 |
| *STUB1* | NM_005861.4 | Spinocerebellar ataxia 48 - AD; Spinocerebellar ataxia, autosomal recessive 16 - AR | PMID: 24113144 |
| *TAC3* | NM_013251.3 | Hypogonadotropic hypogonadism 10 with or without anosmia - AR | PMID: 19079066 |
| *TACR3* | NM_001059.2 | Hypogonadotropic hypogonadism 11 with or without anosmia - AR | PMID: 19079066 |
| *TAX1BP3* | NM_014604.4 | - | PMID: 25645515 |
| *TBC1D20* | NM_144628.4 | Warburg micro syndrome 4 - AR | PMID: 24239381 |
| *TBCE* | NM_003193.3 | Encephalopathy, progressive, with amyotrophy and optic atrophy - AR; Hypoparathyroidism-retardation-dysmorphism syndrome - AR; Kenny-Caffey syndrome, type 1 - AR | PMID: 19491227 |
| *TBX3* | NM_005996.3 | Ulnar-mammary syndrome - AD | PMID: 30550377 |
| *TCF12* | NM_207036.1 | Craniosynostosis 3 - AD; Hypogonadotropic hypogonadism 26 with or without anosmia - AD, AR | PMID: 32620954 |
| *TFR2* | NM_003227.3 | Hemochromatosis, type 3 - AR | PMID: 11313241 |
| *TLE4* | NM_001282748.2 | - | PMID: 30098700 |
| *TRAPPC9* | NM_031466.5 | Intellectual developmental disorder, autosomal recessive 13 - AR | PMID: 27502037 |
| *TRIM32* | NM_012210.3 | Bardet-Biedl syndrome 11 - AR; Muscular dystrophy, limb-girdle, autosomal recessive 8 - AR | PMID: 16606853 |
| *TSPAN11* | NM_001080509.3 | - | PMID: 27502037 |
| *TTC8* | NM_198309.3 | Retinitis pigmentosa 51 - AR; Bardet-Biedl syndrome 8 - AR | PMID: 30886724 |
| *TUBB3* | NM_006086.3 | Cortical dysplasia, complex, with other brain malformations 1 - AD; Fibrosis of extraocular muscles, congenital, 3A - AD | PMID: 23378218 |
| *TYRO3* | NM_006293.3 | - | PMID: 30098700 |
| *WDPCP* | NM_015910.7 | ?Bardet-Biedl syndrome 15 - AR; Congenital heart defects, hamartomas of tongue, and polysyndactyly - AR | PMID:37239474 |
| *WDR4* | NM_033661.4 | Galloway-Mowat syndrome 6 - AR; Microcephaly, growth deficiency, seizures, and brain malformations - AR | PMID: 28617965 |
| *WDR11* | NM_018117.11 | Hypogonadotropic hypogonadism 14 with or without anosmia - AD; Intellectual developmental disorder, autosomal recessive 78 - AR | PMID: 20887964 |

CHH, Congenital Hypogonadotropic Hypogonadism; OMIM, Online Mendelian Inheritance in Man; PMID, PubMed identifier; AR, autosomal recessive; AD, autosomal dominant; XLR, X-linked recessive; DR, digenic recessive; DD, digenic dominant; XLD, X-linked dominant.
